# Expanding the Described Metabolome of the Marine Cyanobacterium *Moorea producens* JHB through Orthogonal Natural Products Workflows

**DOI:** 10.1371/journal.pone.0133297

**Published:** 2015-07-29

**Authors:** Paul D. Boudreau, Emily A. Monroe, Suneet Mehrotra, Shane Desfor, Anton Korobeynikov, David H. Sherman, Thomas F. Murray, Lena Gerwick, Pieter C. Dorrestein, William H. Gerwick

**Affiliations:** 1 Center for Marine Biotechnology and Biomedicine, Scripps Institution of Oceanography, University of California San Diego, La Jolla, California, 92093, United States; 2 Department of Biology, William Paterson University, Wayne, New Jersey, 07470, United States of America; 3 Department of Pharmacology, Creighton University School of Medicine, Omaha, Nebraska, 68178, United States of America; 4 Department of Biology, California State University San Marcos, San Marcos, California, 92078, United States of America; 5 Algorithmic Biology Laboratory, St. Petersburg Academic University, Russian Academy of Sciences, St. Petersburg, 194021, Russia; 6 Department of Mathematics and Mechanics, St. Petersburg State University, St. Petersburg, 194021, Russia; 7 Center for Algorithmic Biotechnology, St. Petersburg State University, St. Petersburg, 194021, Russia; 8 Life Sciences Institute and Department of Medical Chemistry, University of Michigan, Ann Arbor, Michigan, 48109, United States of America; 9 Department of Chemistry and Biochemistry, University of California San Diego, La Jolla, California, 92093, United States of America; 10 Skaggs School of Pharmacy and Pharmaceutical Sciences, University of California San Diego, La Jolla, California, 92093, United States of America; CEA-Saclay, FRANCE

## Abstract

*Moorea producens* JHB, a Jamaican strain of tropical filamentous marine cyanobacteria, has been extensively studied by traditional natural products techniques. These previous bioassay and structure guided isolations led to the discovery of two exciting classes of natural products, hectochlorin (**1**) and jamaicamides A (**2**) and B (**3**). In the current study, mass spectrometry-based ‘molecular networking’ was used to visualize the metabolome of *Moorea producens* JHB, and both guided and enhanced the isolation workflow, revealing additional metabolites in these compound classes. Further, we developed additional insight into the metabolic capabilities of this strain by genome sequencing analysis, which subsequently led to the isolation of a compound unrelated to the jamaicamide and hectochlorin families. Another approach involved stimulation of the biosynthesis of a minor jamaicamide metabolite by cultivation in modified media, and provided insights about the underlying biosynthetic machinery as well as preliminary structure-activity information within this structure class. This study demonstrated that these orthogonal approaches are complementary and enrich secondary metabolomic coverage even in an extensively studied bacterial strain.

## Introduction

**Fig 1:**
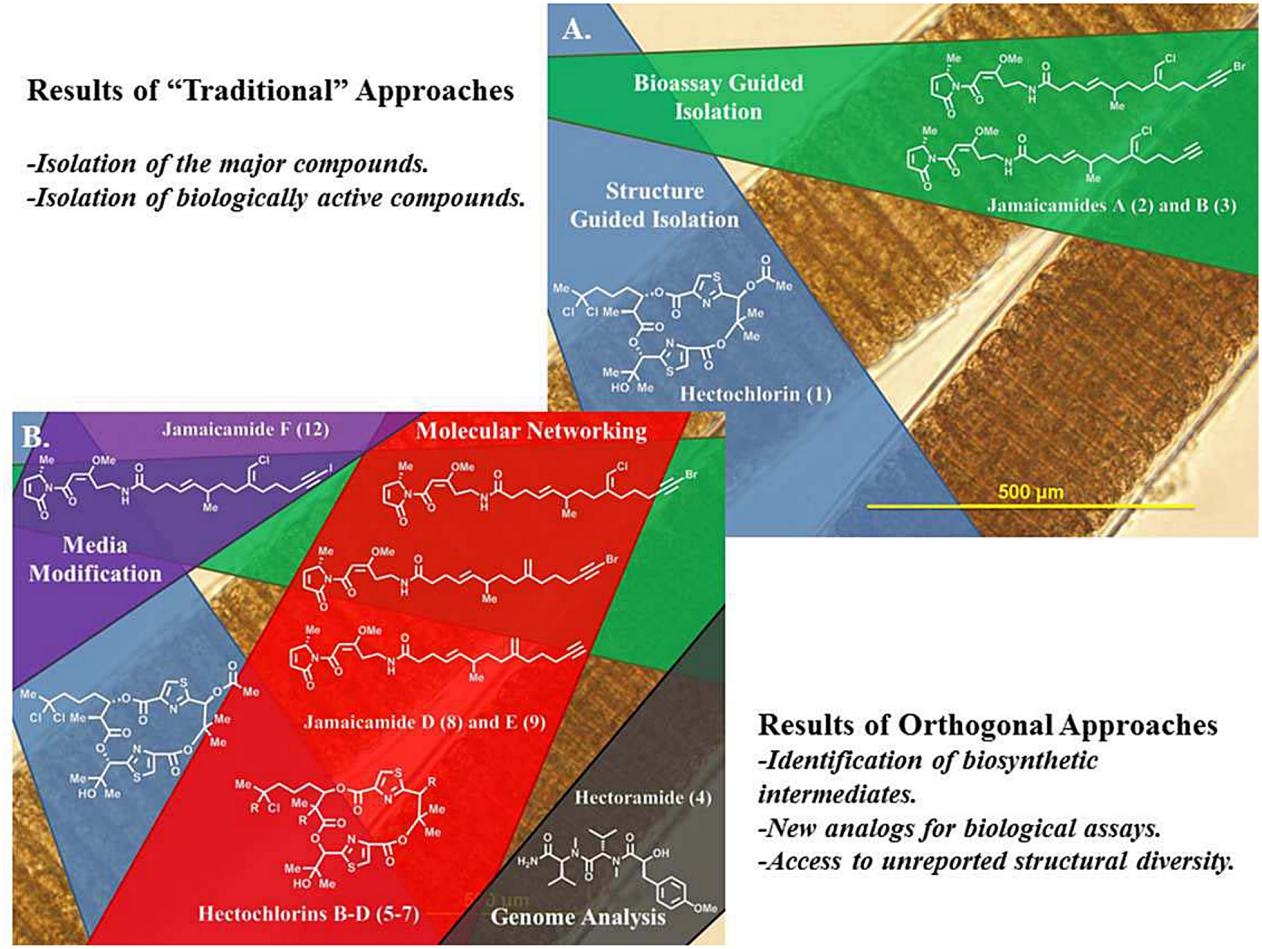
A. The metabolome of *M*. *producens* JHB from prior studies; and B. the expanded metabolome of *M*. *producens* JHB from approaches described in this study.

*Moorea producens* JHB is a strain of tropical filamentous marine cyanobacterium that has been in culture in our laboratory for nearly two decades since its collection from Hector’s Bay, Jamaica in 1996. We have extensively studied its natural products by traditional isolation techniques [1, 2]. An NMR-guided process led to the isolation and structure elucidation of **1**, a highly potent cytotoxin which enhances actin polymerization [1]. In separate work, sodium channel blocking and fish toxicity assays guided the isolation and discovery of **2** and **3** [2]. Since then, this strain has been extensively studied (under its name before reclassification *Lyngbya majuscula* JHB), with cultures from our laboratory being the subject of five more publications [1–7]. In this work we present the application of new methods for natural product discovery being applied to study the metabolome of *M*. *producens* JHB, from which several novel compounds, not observed in previous studies of this strain, were discovered (Fig 1).

Recent developments in the field of natural products have both improved the traditional activity/structure-based isolation workflows, and provided other orthogonal techniques to profile the metabolomes of organisms of interest. Traditional activity/structure-based isolation procedures have been supplemented by the use of solvent extractors [8], improved HPLC systems and columns to improve compound yields [9], new high-throughput screening techniques [10]; or analytical techniques that make it possible to determine the structures of ever smaller quantities of compounds, such as more sensitive cryo-probe NMR systems and new NMR pulse sequences [11, 12]. These improvements have enabled nanomole-scale structure elucidations such as were used to characterize the sanguinamides from the marine nudibranch *Hexabranchus sanguineus* [13]. Concurrently, the field of natural products is exploring novel methods to assess secondary metabolomes that are distinct from traditional isolation workflows, chief among them is genome mining [14]. As genome sequencing has become more rapid and less expensive, and the pipeline for assembling and annotating secondary metabolite pathways from gene sequence information, using programs such as antiSMASH and NaPDos, has become more efficient [15–19]. Consequently, applications of genome mining to the field of natural products have become increasingly diversified [14, 20, 21]. Genome mining has also shown that many strains possess far more biosynthetic gene clusters than previously expected, and indeed, these ‘silent’ pathways in some cases constitute the preponderance of a strain’s biosynthetic capacity [20–22]. Work with the daptomycin producing strain of *Streptomyces roseosporus* showed that it has the capacity to produce three other Non-Ribosomal Peptide Synthetase (NRPS) products, namely arylomycin, napsamycin, and stenothricin, all previously unreported from this organism [22].

Even with these impressive enhancements to our isolation methodologies, significant challenges remain to fully characterize the metabolic potential of an organism. Any single workflow has drawbacks; bioassay-guided isolation methods are inherently biased and overlook metabolites with differing activity. Structure-guided isolation schemes can miss compounds that are produced at low concentration and thus escape detection by normal methods, or if they have unremarkable spectroscopic properties. In addition, it is never known whether an organism is expressing all of its secondary metabolite biosynthetic pathways under a given set of environmental or culture conditions [23]. While a successful genome mining effort might fully characterize the biosynthetic pathways within an organism, connecting and coordinating this with an isolation workflow can be challenging [24]. Without heterologous expression or knockout experiments, links between a biosynthetic pathway and a structure are often only tentative.

In our decision to reexamine the metabolome of *Moorea producens* JHB we were mindful of these challenges, but also of the many examples in the literature where novel techniques found previously unreported compounds from productive well studied strains. Since the first report from *M*. *producens* JHB, our laboratory has gained access to new assays, such as modulation of cathepsin L activity [25], and new facilities on the UCSD campus for high-field NMR and High-Resolution Mass Spectrometry (HRMS) [26]. However, because **1** and **2** are the major secondary metabolites from this strain, we knew that any additional compounds would be minor metabolites, and hence, alternative techniques that could target these compounds were needed.

Mass spectrometry based-molecular networking is well suited to serve as an alternative metabolic profiling platform because it is highly sensitive, amenable to use with compound or culture libraries, and blind to traditional guideposts for isolation projects such as chromatographic retention time or halogen isotope patterns [27, 28]. Mass spectrometry is highly sensitive, and while no ionization technique is universal, electrospray ionization (ESI) effectively ionizes a wide range of structural classes providing good coverage of the metabolome [29].

**Fig 2:**
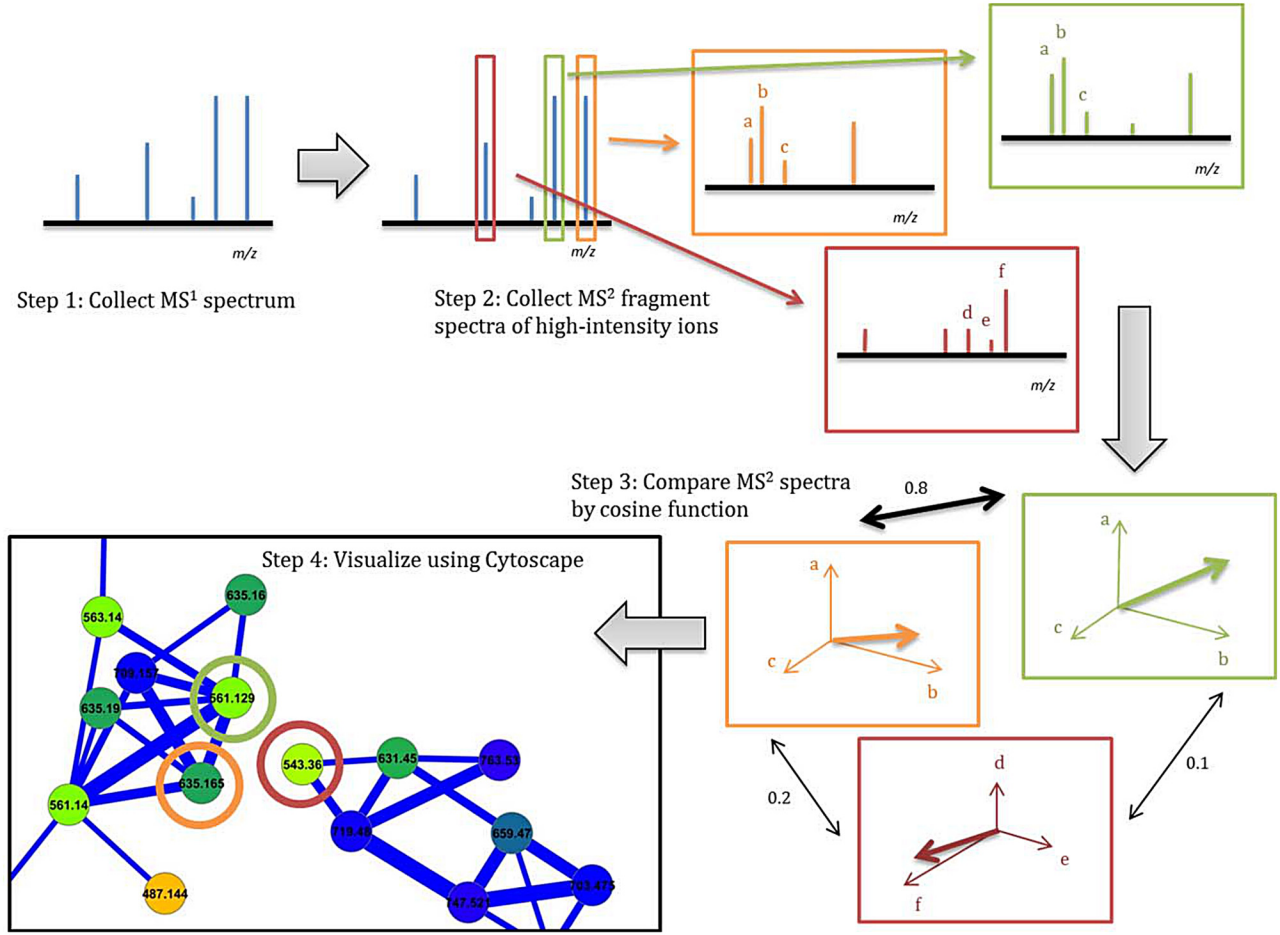
Visualization of MS data with Molecular Networking. The process of forming a molecular network begins with collection of MS^1^ spectra (Step 1), from which parent ions are selected for MS^2^ fragmentation (Step 2). After the data is acquired it is processed by the Spectral Networking algorithm which converts MS^2^ data to vectors. The minimum number of peaks required to construct a vector is six; in this figure it is represented as only three so that the vector could be presented visually. It is important to note that when the multi-dimensional vectors are compared against all others by cosine function the vectors can have hundreds of dimensions (Step 3). This output is then visualized in Cytoscape with each node representing a MS^2^ spectrum labeled and colored based on its parent mass (Step 4).

Molecular networking utilizes MS^2^ data to sort parent ions based on their structural similarity. Secondary ion mass fragmentation data relate directly to molecular structure because chemical bonds break on the basis of bond strength, strain within a molecule, and ability of a fragment to stabilize charge [27]. Fragmentation patterns are thus intimately related to molecular structure, but independent of other bases for assessing compound similarity, such as the parent ion mass, halogen isotope pattern, or LCMS retention time. The Spectral Networking algorithm normalizes the intensity of fragment ions, uses each as an independent axis to construct a multidimensional vector for each spectrum, and finally compares the similarity of these vectors using a cosine function [27]. This cosine score is then used to plot the relationships between different parent ion masses with the open source software Cytoscape (Fig 2) [30].

For *M*. *producens* JHB, a crude extract of the cyanobacterium was profiled by LCMS and data from these runs were constructed into a molecular network as described above. This network identified an array of previously unreported compounds that were related to the major metabolites **1** and **2**, likely produced as intermediates along the biosynthetic pathway, or by promiscuity in the substrate binding sites of these modular-type biosynthetic enzymes. These analogs provide new insights into the biosynthetic machinery and a more thorough understanding of the true secondary metabolic capacity of *M*. *producens* JHB than accomplished in previous work.

Additionally, further insight into the biosynthesis of **2** was gained by growing *M*. *producens* JHB in seawater media supplemented with sodium iodide. Under these conditions a minor metabolite of the jamaicamide family was produced in large quantities, and allowed the isolation and characterization of the iodinated analog of **2**. This also provided insight into the halogenation enzyme involved in this biosynthetic step as well as yielding sufficient compound for biological assays.

The two approaches described above revealed new compounds within the jamaicamide and hectochlorin families, products of the two biosynthetic gene clusters previously identified in this organism [2, 4, 5]. To assess whether *Moorea producens* JHB possessed a broader biosynthetic capacity, we complemented the molecular networking approach with an in-depth bioinformatics interrogation of the sequenced genome. This led to the detection of numerous short NRPS pathways that were predicted by anti-SMASH to encode for small polar peptides, as well as one somewhat longer pathway for a predicted water soluble compound, the structure of which will be presented in a separate publication. An effort to isolate the products of these shorter NRPS pathways led to the isolation and characterization of a new compound, hectoramide (**4**), containing two *N*-methyl valine residues and a modified tyrosine residue. The structure of this new peptide was determined by 2D-NMR and HRMS analysis, and configuration of the residues by hydrolysis and chiral chromatographic analysis.

## Results and Discussion

### Molecular networking of *M*. *producens* JHB

The MS data from the crude extract LCMS chromatograms of *M*. *producens* JHB were analyzed using the Spectral Networking tool to create a molecular network and visualized in Cytoscape (Fig A in S1 Text) [27, 30]. These data were also uploaded into the Global Natural Products Social Molecular Networking database where they are publicly available (Massive ID MSV000078990) [31].

**Fig 3:**
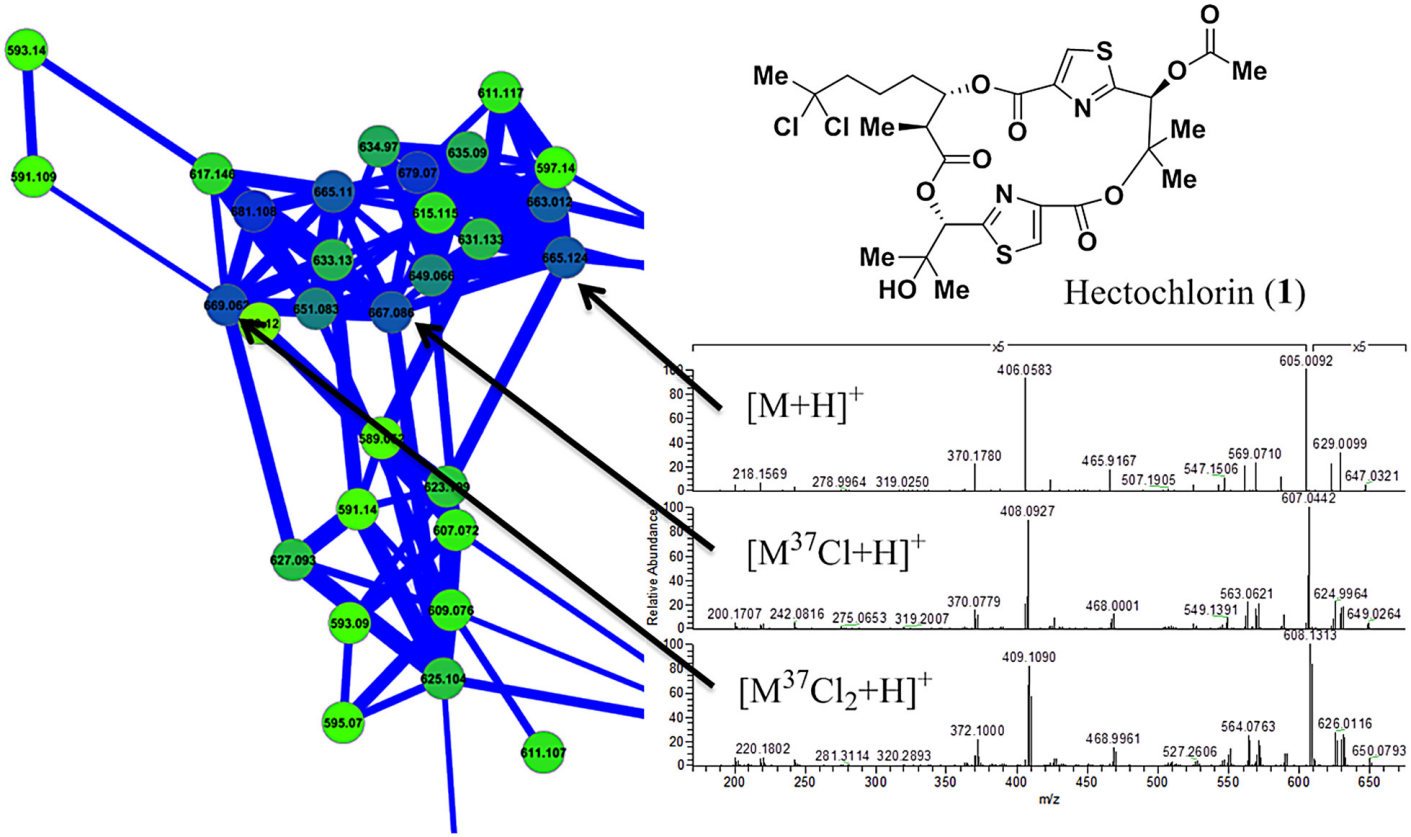
Hectochlorin (1) isotope pattern within the *M*. *producens* JHB network. The presence of ^35^Cl or ^37^Cl within specific hectochlorin molecules yielded different parent masses and fragment spectra. The species with both ^35^Cl atoms has an *m/z* of 665, the species with one ^35^Cl and one ^37^Cl atom has and *m/z* of 667, and the species with ^37^Cl atoms has an *m/z* of 669. Because of this the fragment spectra share only masses from those fragments without chlorine atoms, and those fragments bearing the chlorine atoms show the same mass differences as their parent masses.

**Fig 4:**
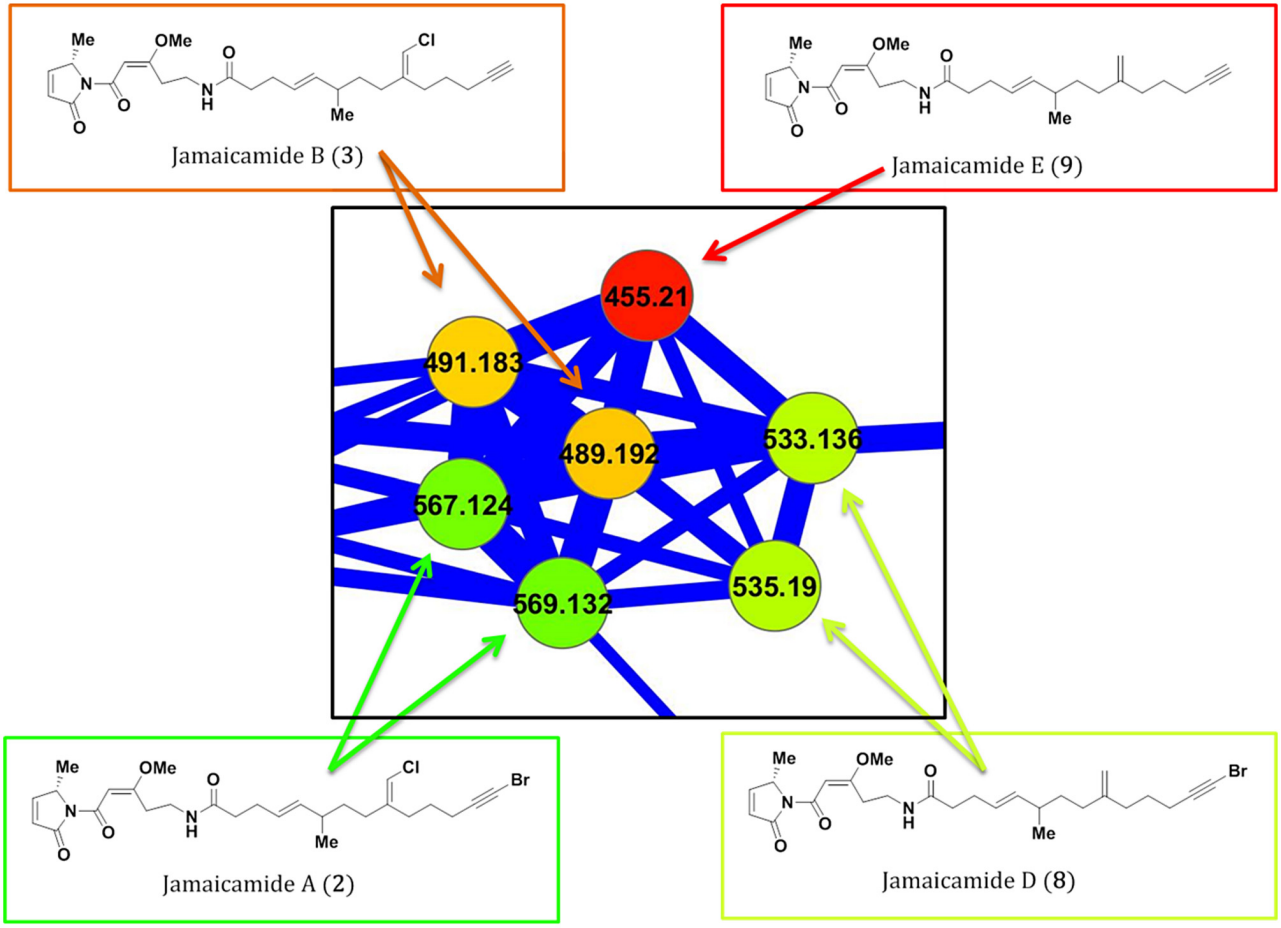
The jamaicamide cluster of nodes within the Molecular Network of *Moorea producens* JHB. As with the hectochlorins, shown in Fig 3, the jamaicamides are present in the network as multiple parent masses from the presence of heavy halogen atoms.

The network was interrogated for the [M+H]^+^ ions from the known major metabolites from this cyanobacterial strain, **1** and **2**. In both cases, this revealed a cluster of parent masses from related analogs in the same family of compounds (Figs 3 and 4). One important caveat of the molecular networking process is that a single compound can give rise to multiple nodes. For example ions deriving from different adducts, from differing isotope composition, as well as source fragments, are all observed in the molecular network when present in sufficient intensity for the spectrometer to collect their MS^2^ spectra. The hectochlorin cluster is clearly an illustration of a single molecule producing multiple nodes from different halogen isotopes. The presence of heavy chlorine atoms in some of the hectochlorin molecules results in three nodes with mass differences of two Daltons within the hectochlorin cluster (Fig 3).

The hectochlorin cluster was also interrogated for new hectochlorin analogs; many of the masses that clustered with the nodes from compound **1** were consistent with hypothetical analogs. The LCMS trace showed these species had distinct retention times from that of **1** (i.e. they were not source fragments), as well as isotope patterns that matched their variable levels of halogenation. Using the structure of **1** as a template, a series of hypothetical compounds could be proposed which were consistent with these masses (Table 1).

**Table 1:** Analogs of Hectochlorin Deduced from the Molecular Network.

Compound Name and Number	Structural Features	Halogenation Pattern	Retention Time	Masses Observed in Network
**Hectochlorin (1)**	Original Compound	Dichloro	23.8 min	665/667/669
**Hectochlorin B (5)**	Deacyl-hectochlorin	Dichloro	20.3 min	623/625
**Hectochlorin C (6)**	Dechloro-hectochlorin	Monochloro	22.6 min	631/633
**Hectochlorin D (7)**	Methyl-hectochlorin	Dichloro	25.8 min	679/681

The network was also examined for new analogs within the jamaicamide cluster. Both **2** and **3** were present as a pair of [M+H]^+^ ions separated by 2 Da, again due to incorporation of heavy halogen atoms (Cl, Br). Another pair of nodes, with parent masses also separated by 2 Da, clustered with high cosine scores to the known jamaicamides. The LCMS^1^ chromatogram showed that this pair of nodes possessed a distinct retention time, and that it had an isotopic composition consistent with a singly brominated but non-chlorinated species. Thus, a dechloro-analog of **2**, jamaicamide D (**8**), was hypothesized to explain this pair of connected nodes and was supported by HRMS data [*m/z* 533.1983, retention time 29.9 min, C_27_H_38_BrN_2_O_4_ [M+H]^+^ calculated 533.2009, -2.6 mamu].

The biosynthetic gene cluster and many of the individual biosynthetic steps for **2** are known, and previous work in our laboratory has suggested that **3** is the precursor for **2** with bromination occurring as the final step in the pathway [2, 3, 5, 6]. In this scenario, **8** would be formed by bromination of a non-chlorinated precursor (Fig AD in S1 Text). Further inspection of the network revealed a mass consistent with such a non-halogenated species, termed jamaicamide E (**9**), also clustered in the jamaicamide family (Fig 4). The LCMS^1^ trace again showed a distinctive retention time for **9**, and an HRMS formula supportive of this structure [*m/z* 455.2877, retention time 26.5 min, C_27_H_39_N_2_O_4_ [M+H]^+^ calculated 455.2904, -2.7 mamu].

### Purification of analogs from the jamaicamide and hectochlorin families

The LCMS analysis of this cyanobacterial extract used a serial set of five scans; one high-resolution, one low-resolution, and then three LTQ-MS^2^ scans of the three most intense ions from the previous scan using dynamic exclusion. Low-resolution LTQ scans were used to generate the MS^2^ data for molecular networking because of the higher sensitivity of this mode. This analysis generated MS^1^ and MS^2^ data consistent with the proposed structures for the jamaicamide and hectochlorin analogs. To rigorously characterize these new compounds, an extract that had been preparatively fractionated by Vacuum Liquid Chromatography (VLC) was interrogated for analogs [32]. By LCMS analysis, fractions eluting with 80% EtOAc/hexanes and 100% EtOAc contained hectochlorin and the jamaicamide metabolites. These two VLC fractions were combined and further purified by Reverse-Phase Solid-Phase Extraction (RP-SPE) and RP-HPLC. The two VLC fractions eluting in 25% methanol to ethyl acetate, and in 100% methanol, were found to contain additional hectochlorin analog masses by LCMS, and were combined and purified in the same fashion as the less polar fractions.

These HPLC purifications afforded pure **1**, **2**, **3**, and several of the minor analogs proposed by molecular networking analysis of the crude extracts. The major metabolites **1** and **2**, along with the new derivatives **8** and **5**, were isolated in sufficient quantity to fully characterize by NMR spectroscopy. However, not all of the species identified from the network were present in isolable amounts, as in the case of **9**. To better characterize these other more minor analogs, they were subjected to HRMS^2^ fragmentation analysis, and then assembled *de novo* using the previously reported cyclic peptide dereplication tool [33].

The observation that **5**, the deacetyl-analog, was the most abundant analog of compound **1**, is consistent with the conjecture that this is an intermediate in the biosynthesis of **1** [4]. The gene cluster encoding for hectochlorin biosynthesis shows no evidence of an acylation enzyme in the NRPS domain responsible for constructing the cyclic core of the molecule [4]. Indeed, in the original report on the biosynthetic gene cluster, it was noted that the *hct* gene cluster lacked an acylation enzyme; it was proposed that acylation occurs as a post-NRPS modification [4]. Moreover, **5** was previously reported along with **1** from the sea hare *Bursatella leachii*. In that report the authors suggested that accumulation of **5** in *B*. *leachii* could be the result of metabolism of **1** by the sea hare [34]. With direct isolation of **5** from the cyanobacterial source, however, it is clear that direct accumulation from its diet is also a possibility.

At the time of its isolation, **5** was found to be a more potent cytotoxin than **1** against the human carcinoma of the nasopharynx (KB) and human small cell lung cancer (NCI-H187) cell lines (LD_50_’s of 0.31 μM and 0.32 μM for **5**, compared to 0.86 μM and 1.20 μM for **1**) [34]. Molecular networking groups compounds based on their similar structural frameworks irrespective of their abundance, polarity, or other characteristics such as halogenation pattern or biological activity. Therefore, metabolites with significantly different polarities within an extract can still be easily recognized as structural analogs by molecular networking. The majority of compound **5** was found in the last two and most polar VLC fractions, which are often complex mixtures with poorly soluble nuisance compounds such as salts and glycolipids, potentially masking compounds of interest. By MS-based profiling and then targeting the isolation process towards the isolation of a suite of structurally similar products, rather than a single compound, information about structure activity relationships can be developed, as well as insights gained into the biosynthetic process.

Besides **5**, the other analogs of **1** detected in this investigation are likely biosynthetic shunt products. They may be produced by errors in loading of the biosynthetic substrates or in the reactions the biosynthetic enzymes catalyze. For example, the monochlorinated analog of **1**, hectochlorin C (**6**), is likely produced by a process wherein the chlorination enzyme fails to catalyze the addition of a second chlorine atom to the substrate, and this modification is transparent to subsequent downstream biosynthetic steps. However, the mono-chloro group in **6** is curious because formation of the trichloromethyl group of barbamide (**10**) in a different strain of *M*. *producens*, which is catalyzed by a highly homologous enzyme [35–37], occurs without going through a discrete monochlorinated intermediate. Rather, in the case of barbamide, the biosynthesis proceeds through an initial dichlorination step catalyzed by the BarB2 halogenase protein, followed by a monochlorination step catalyzed by the BarB1 halogenase protein to yield the final trichlorinated product. The isolation of **6** reveals that the halogenation enzyme in hectochlorin biosynthesis is different from the barbamide halogenases, as neither barbamide enzyme was observed to catalyze the formation of a discrete monochlorinated species in a detectable yield, and as the barbamide proteins act on the less reactive terminal methyl species [35–37].

### Genomic Insights into *M*. *producens* JHB Natural Products

**Fig 5:**
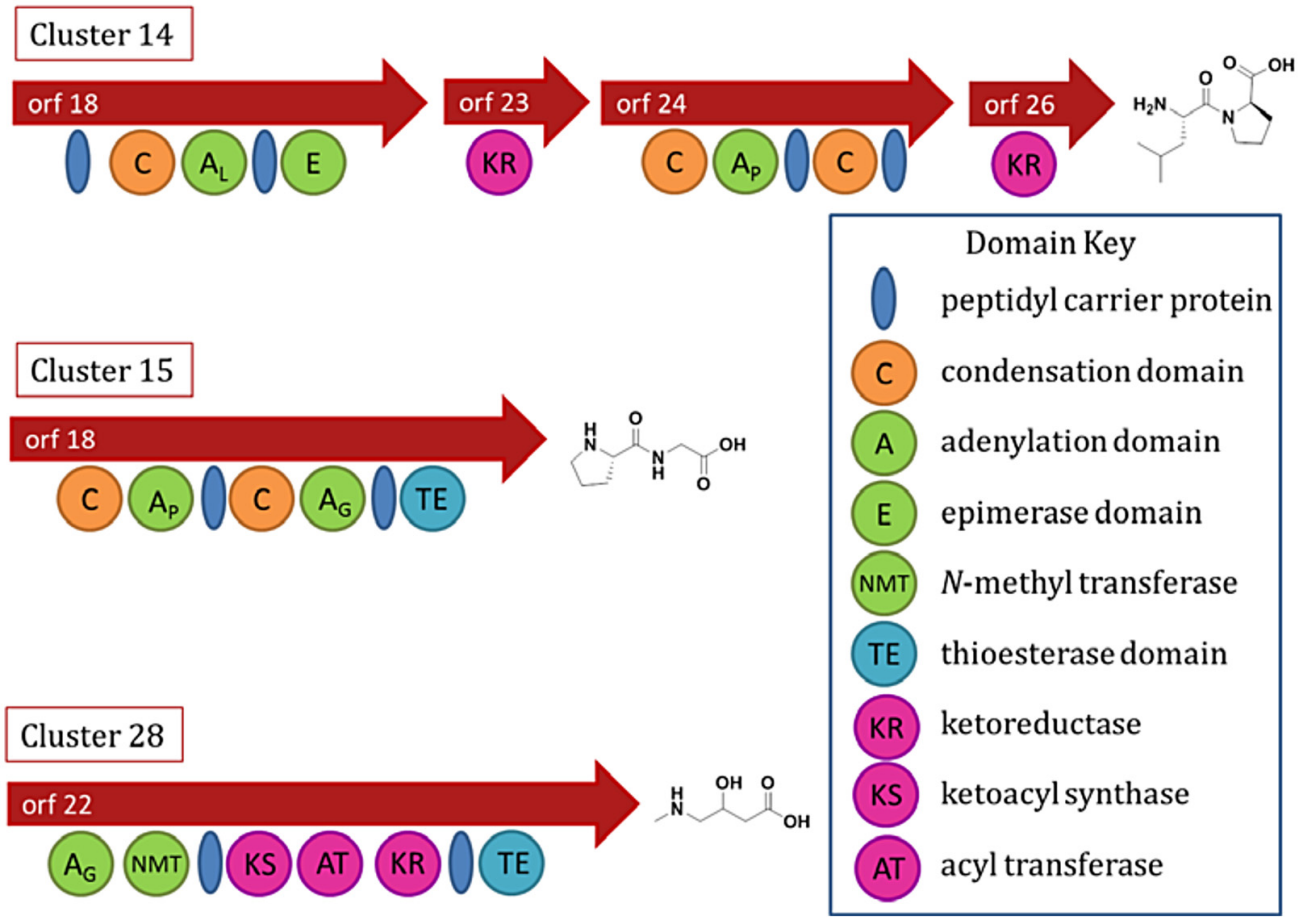
Three NRPS or NRPS/PKS-type biosynthetic gene clusters from the *Moorea producens* JHB genome, annotated by antiSMASH for predicted products.

Genome sequence analysis of *M*. *producens* JHB revealed the known biosynthetic pathways of hectochlorin and the jamaicamides [2, 4, 5]. Other numerous short biosynthetic pathways were annotated by antiSMASH, and many of the predicted products of these pathways were dipeptides (Fig 5). Based on their predicted structures, we posited that if produced these compounds would be present in the more polar VLC fractions. A careful search of the metabolome contained in the polar fractions by LCMS analysis showed the dominant compound in the most polar eluting fractions was the hectochlorin analog **8**. However, during purification by RP-SPE followed by RP-HPLC, not only was **8** isolated, but also a novel compound, named here as hectoramide (**4**), which was thoroughly characterized by NMR, HRMS and chemical degradation to components that could be compared with standards.

Analysis of the TOtal Correlation SpectroscopY (TOCSY) spectrum for compound **4** revealed four spin systems. Two of these were valine residues, seen by correlations of two doublet methyl peaks to their respective β-proton multiplets, and from there to their α-protons. Both of these residues were shown to be *N*-methyl valine residues by reciprocal Heteronuclear Multiple Bond Correlation (HMBC) correlations between the *N*-methyl singlets and the α-carbons. The presence of a para-substituted aromatic ring was revealed by a COSY correlation between two doublet 2H aromatic peaks; one of the substituents was revealed to be an *O*-methyl by HMBC signals between the singlet methyl protons at δ 3.79 and an aromatic ring carbon (C-19). The final COSY spin system connected an α-proton and a β-methylene group; the latter showed HMBC correlations to several of the aromatic ring carbons, and thus explained the other ring substituent. The downfield shift of the α-carbon (δ 69.9) and α-proton (δ 4.52) indicated this position was substituted with a hydroxy group rather than an amino group, revealing the final residue to be a 3-(4-methoxyphenyl)lactic acid (Mpla).

**Fig 6:**
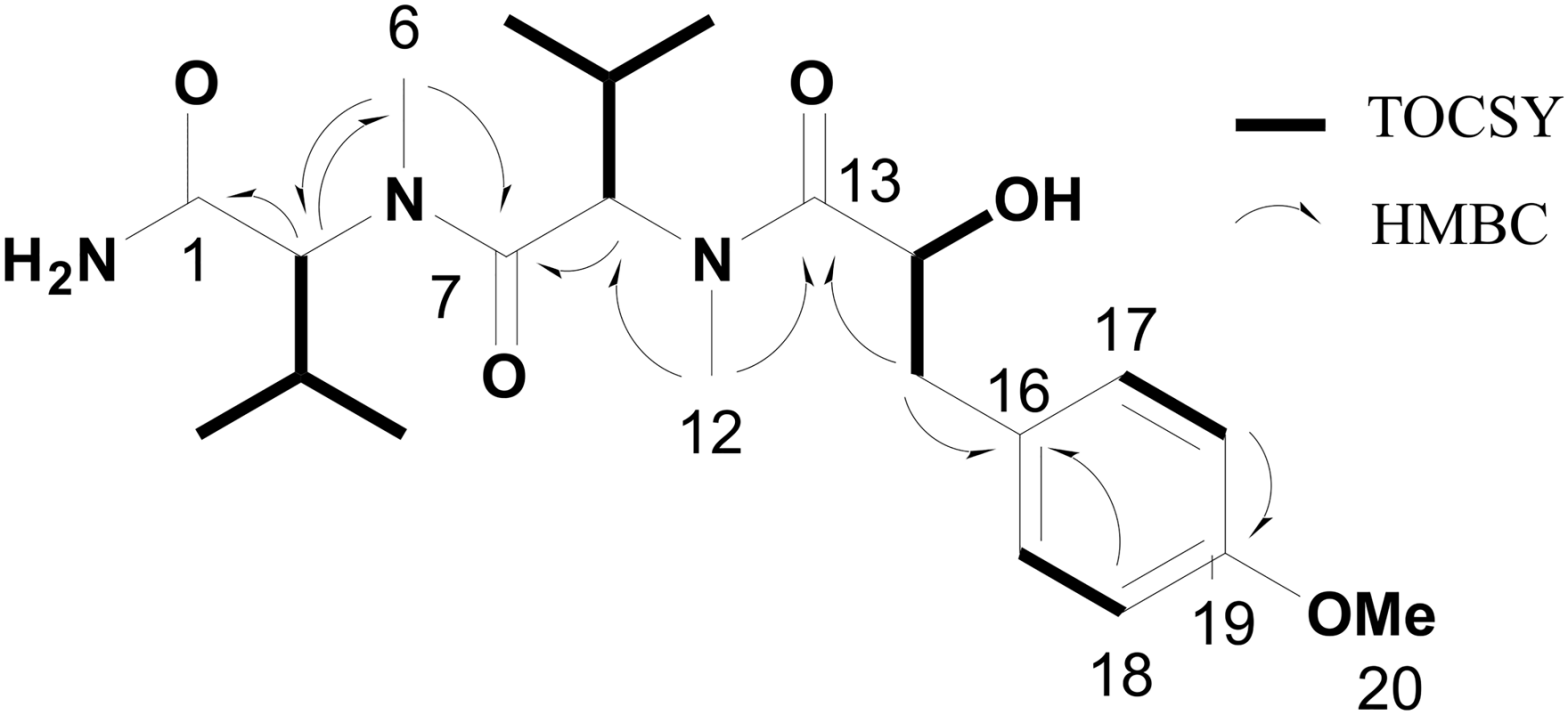
TOCSY and key HMBC correlations in hectoramide (4).

The linkage of these residues was determined by 2D-NMR and MS^2^ fragmentation analysis. The *N*-methyl group of the first valine showed a correlation to the carbonyl of the second valine residue. In turn, this latter valine showed a correlation from its *N*-methyl to the carbonyl of the adjacent Mpla residue. As such, the initial structure proposed for this compound was (HOOC-*N*-Me-Val)-(*N*-Me-Val)-(Mpla-OH) (**11**); however, this was inconsistent with data from HRMS analysis. While the structure **11** would have a predicted molecular formula of C_22_H_34_N_2_O_6_, the MS^1^ [M+H]^+^ peak of this compound was at *m/z* 422.2648 Da, consistent with the molecular formula of C_22_H_35_N_3_O_5_. This was resolved by comparing predicted ^13^C-NMR shifts with various candidate structures, and indicated that a terminal primary amide and hydroxy group were fully consistent with the C-1 and C-14 ^13^C-NMR shifts (Fig AT in S1 Text). In addition, MS^2^ fragmentation showed fragment ions diagnostic for this structure (Fig 6), explicitly (H_2_NOC-*N*-Me-Val)-(*N*-Me-Val)-(Mpla-OH).

The absolute configuration of **4** was elucidated by traditional hydrolysis and derivatization methods. The two *N*-methyl-valine residues were characterized by Marfey’s analysis [38, 39], and the Mpla was derivatized with (2*S*)-2-octanol for chiral GCMS analysis [40]. These analyses established the configuration of both valine residues as L, and the Mpla moiety as *S*.

Based on the structure of **4**, its biosynthetic pathway would be expected to have three adenylation domains, coding for one tyrosine and two valine residues, and three methylation domains, two for amide *N*-methylations and one for the phenol *O*-methylation. In this regard, the planar structure of **4** is different from any of the predicted antiSMASH molecules for the pathways observed in the *M*. *producens* JHB genome (Fig 5). To refine the predicted structures of these pathways, the adenylation domains were submitted to NRPSpredictor2 [41–43]. This improved our confidence in the predictions from antiSMASH, but also strongly suggested that these pathways were not responsible for the production of **4**. The genome was further interrogated specifically for valine and tyrosine adenylation domains and methyltransferases. Searching the genome for these targets found several hits; however, each was inconsistent with the expected biosynthetic pathway of **4**. The bacteriocin pathways annotated by AntiSMASH were also inconsistent with the biosynthesis of **4** because the necessary sequence “YVV” was not found in the leader peptide sequences, and because there were no methylation domains in these clusters.

If **4** was produced by a heterotrophic bacterial strain living in association with *M*. *producens* JHB, this might explain why the pathway was not found in the *Moorea producens* JHB genome. Because *Moorea producens* JHB is not axenic, the raw genome data is actually a rarefied metagenome of DNA from both the cyanobacteria and associated heterotrophic bacterial strains. After the DNA sequence data were assembled, it was binned into groups of high (generally heterotrophic) or low (generally cyanobacterial) G+C content. To continue the search for a **4** biosynthetic pathway, the high-G+C scaffolds were also evaluated with antiSMASH, and revealed three additional biosynthetic gene clusters. However, none of the latter pathways were NRPS pathways; and an additional bacteriocin pathway contained neither a leader sequence consistent with **4**, nor methylation domains. As none of the pathways found in the *M*. *producens* JHB metagenomic data could convincingly be attributed to **4** biosynthesis, this leaves the origin of this compound uncertain; possibilities include that the compound is produced by a fragmented pathway, a pathway that was not annotated by AntiSMASH, or by a heterotrophic bacterium in the culture but for which the genetic material was not captured by this genome sequencing work.

### Jamaicamide Halogenation

Molecular networking analysis of the *M*. *producens* JHB extract revealed that the pathways for jamaicamide and hectochlorin had a greater capacity for analog production than was previously appreciated. To further probe this, specifically the process of halogenation in the jamaicamides, filaments of *Moorea producens* JHB were grown in SW-BG11 media that was supplemented with sodium bromide and sodium iodide in equimolar concentrations (10 mM each) above the natural abundance of bromide (0.84 m*M*) or iodide (0.2–0.5 μ*M*, average of 0.4 μ*M*) in seawater [44]. Profiling of the crude extract of this small scale experiment by LCMS with molecular networking analysis indicated an additional mass and fragmentation pattern consistent with an iodinated analog of **2**, named here as jamaicamide F (**12**) (Figs H and I in S1 Text).

**Fig 7:**
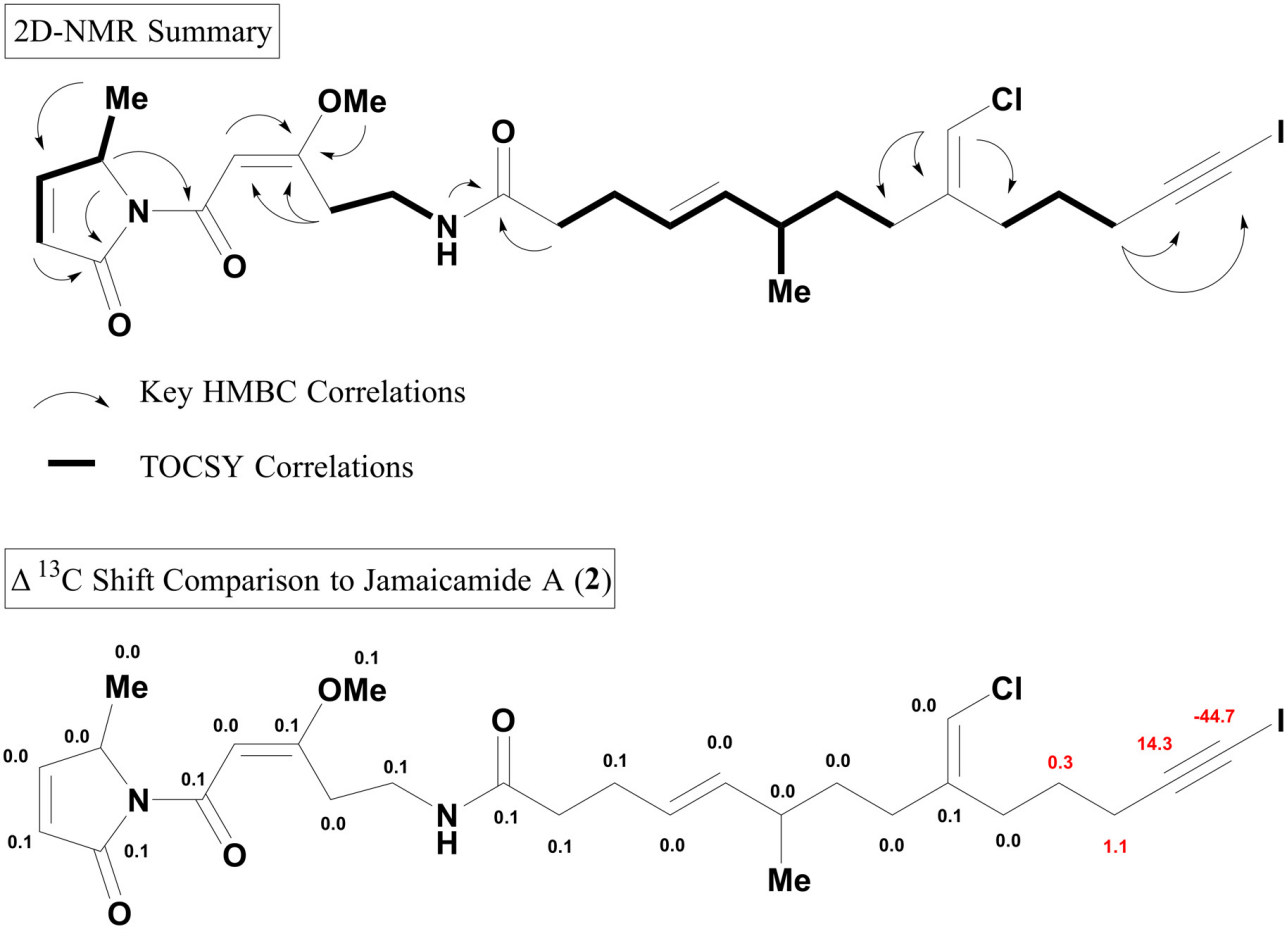
NMR Data for Jamaicamide F (12).

To confirm the structure of compound **12**, multiple larger scale cultures of *M*. *producens* JHB were grown in media supplemented solely with sodium iodide (10 mM). After growth for 8–9 weeks the cultures were extracted and the crude extract purified by sequential RP-SPE and RP-HPLC to afford **12**. Its structure was confirmed by NMR analysis, and generally showed a dataset very similar to that of **2**. All carbon atoms in **12** were within 0.0–0.1 ppm of those in **2** except for those four closest to the terminal alkyne, suggestive of conservation of structure and configuration between these two analogs (Fig 7). The iodo-alkyne functionality possessed distinctive chemical shifts of -6.6 and 94.2 ppm for C-1 and C-2, respectively (Table AI and Fig AK in S1 Text). The original crude extract chromatogram of *M*. *producens* JHB grown in normal SW-BG11 media was subsequently inspected for the presence of **12**. Indeed **12** could be observed in the MS^1^ extracted ion chromatogram at *m/z* 615 with a distinct retention time (29.4 min), and is thus to our knowledge the first report of a naturally occurring iodo-alkyne. However, it was of too low intensity to be selected for fragmentation and observed by the MS^2^-based molecular network. Despite the fact that iodide salts are not specifically included in SW-BG11 media, they are present as trace constituents in sodium chloride (bromide averaging 56 ppm and iodide averaging 0.24 ppm in Instant Ocean brand sea salt [45]), thus explaining the source of iodine to produce compound **12** in cultures prepared in SW-BG11 media.

It was interesting that when *M*. *producens* JHB was grown in media with an equal molar abundance of bromide and iodide ions, the two compounds **2** and **12** were observed in the LCMS chromatogram with equal intensity. While ion intensity can be difficult to quantitate, in this case because the difference in structure is replacement of a bromine atom with an iodine atom, it is likely that compounds **2** and **12** ionize comparably. The production of **2** and **12** in equal amounts in this experiment suggests that the enzyme is not selective for bromine or iodine. Halogenation enzymes can derive their selectivity from differences of halide electronegativity, abundance in nature, ionic radius and reactivity [46, 47]. Fluoride’s paramount electronegativity results in the fact that enzymes incorporating this halogen are both rare, and unusually for halogenases, non-oxidative [47]. In seawater fluorine, bromine, and iodine are relatively rare with F/Cl/Br/I molar ratios of 2,000:1,000,000:2,000:1 [44]. Thus, it appears that the enzyme responsible for bromination of jamaicamide excludes iodine on the basis of its relative scarcity in seawater, yielding **2** as the major product in nature.

Previous work on the jamaicamide biosynthetic pathway showed that hexanoic acid, 5-hexenoic acid, and 5-hexynoic acid but not 6-bromo-5-hexynoic acid were suitable substrates for JamA [3]. By in vivo MALDI-TOF MS analysis following ^15^N labelling, it was found that production of **3** is light dependent, whereas production of **2** continues in the dark, and that **2** can be produced without fresh production of **3** [6]. These data strongly support the proposal that the biosynthesis proceeds through a final bromination step on a pool of free **3** to afford **2**.

**Fig 8:**
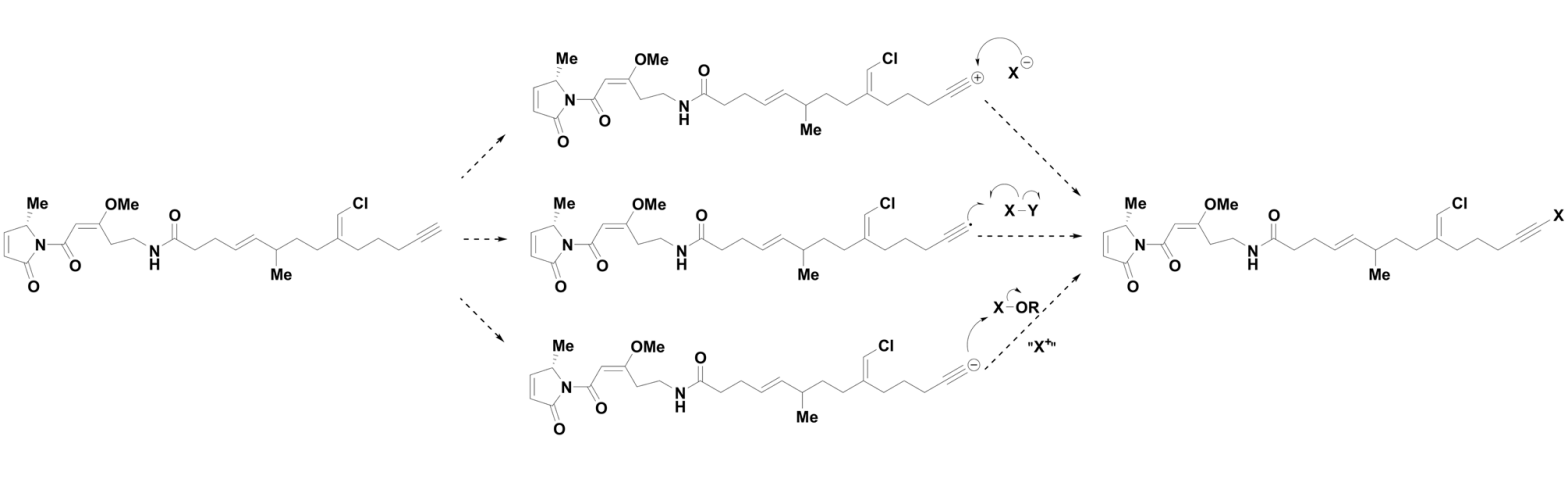
The Potential Mechanisms of Halogenation of the Alkyne in Jamaicamide.

Three mechanisms of bromination of **2** are conceivable using Br^-^ by a nucleophilic halogenase, Br^⦁^ by a non-heme iron O_2_-dependent halogenase, or ‘Br^+^’ by a haloperoxidase or an FADH_2_-dependent halogenase (Fig 8) [47, 48]. Assuming bromination occurs on the terminal alkyne, as discussed above, then halogenation by a nucleophilic halogenase Br^-^ is unlikely because this would putatively proceed through a high energy *sp* hybridized alkynyl carbocation species, which is highly disfavored over the resonance stabilized propargyl cation [49]. Bromination through the alkynyl radical is a conceivable mechanism, but the enzyme activity profile of reacting with both bromine and iodine and being unable to react with abundant chlorine is different from the reactivity profile of the known radical halogenases. Known non-heme iron O_2_-dependent halogenase can catalyze both chlorination and bromination (with chlorination being highly favored), and no reported iodination activity [48]; this may be due to the steric constraint of the larger iodide or because of a difference in fundamental energetics, radical iodination with elemental iodine is an endothermic process [49]. Bromination through a hypohalite ‘Br^+^’ species is compatible with the observation of an iodinated species and no chlorinated species because bromoperoxidases are capable of oxidizing the less electronegative iodine but not the more electronegative fluorine or chlorine [46]. Flavin-dependent halogenases have not been observed to iodinate, hypothesized as a problem of sterics for the larger iodide or hydrolysis of the bound iodine intermediate, so they are a poorer candidate for producing the reactive “X^+^” equivalent [48].

Walsh and coworkers suggested an alternative mechanism for the bromination of jamaicamide via radical bromination of a saturated precursor methyl terminus [50]; however, this seems flawed for two possible reasons. First, as noted above, this terminal halogenation appears to occur with the alkyne rather than the alkane. Additionally, as stated above, with the discovery of **12**, the reactivity profile of acting on bromine and iodine but not chlorine does not match to any known non-heme iron and O_2_-depenedent halogenase. Therefore, electrophilic addition by a haloperoxidase remains as the most likely mechanism for the bromination/iodination event in jamaicamide biosynthesis.

### Ion Channel Pharmacology of the Jamaicamides

The ability of compounds **2**, **3**, and **12** to antagonize veratridine-stimulated Ca^**2+**^ influx was studied in neocortical neurons. All three jamaicamides tested (**2**, **3**, and **12**) produced concentration-dependent antagonism for the increase in neuronal [Ca^**2+**^]_**i**_ induced by veratridine (Fig AP in S1 Text). The concentration-response curves were fit by a three-parameter logistic equation yielding IC_**50**_ values of 1.82 μM (95% CI = 0.9–3.3 μM), 6.88 μM (95% CI = 3.0–15.6 μM) and 4.3 μM (95% CI = 2.2–8.4 μM), respectively for **2**, **3**, and **12**. The results suggest that **2** is approximately 2–3 times more potent than the other two jamaicamides tested.

Given that **2** and **3** have previously been reported to have sodium channel blocking activity at a concentration of 5 μM in Neuro-2a mouse neuroblastoma cell lines [2], we assessed their ability to block veratridine-stimulated sodium influx. We used primary cultures of mammalian neurons as a model system that is more relevant than transformed cell lines to mammalian neurotoxicology [51]. We determined the ability of these compounds to antagonize veratridine-stimulated Na^+^ influx in neocortical neurons. All three jamaicamides (A, B and F) produced concentration-dependent antagonism of the increase in neuronal [Na^+^]_i_ induced by veratridine (Fig AQ in S1 Text). The concentration-response curves were best fit by a three-parameter logistic equation yielding IC_50_ values of 1.1 μM (95% CI = 0.5–2.5 μM), 3.6 μM (95% CI = 1.5–8.5 μM) and 2.3 μM (95% CI = 1.0–5.0 μM), respectively for **2**, **3**, and **12**. Again, our results suggest that **2** is approximately 2-3-times more potent than the other two jamaicamides as a sodium channel blocker in neocortical neurons. Collectively, these data indicate that the structural differences between **2**, **3**, and **12** have only minor roles in the interaction of these compounds with voltage-gated sodium channels.

## Conclusions

The results of using molecular networking, genome analysis, and directed biosynthetic feeding studies with *M*. *producens* JHB illustrated that each approach has the potential to enhance the traditional structure or bioassay-guided natural products workflows. Just as importantly, it revealed that previous studies have overlooked metabolic diversity in the hectochlorin and the jamaicamide structural families. This is not because the previous studies were poorly designed or executed, but because these previous methods were inherently less comprehensive and thus do not fully explore the secondary metabolome of a given organism.

**Fig 9:**
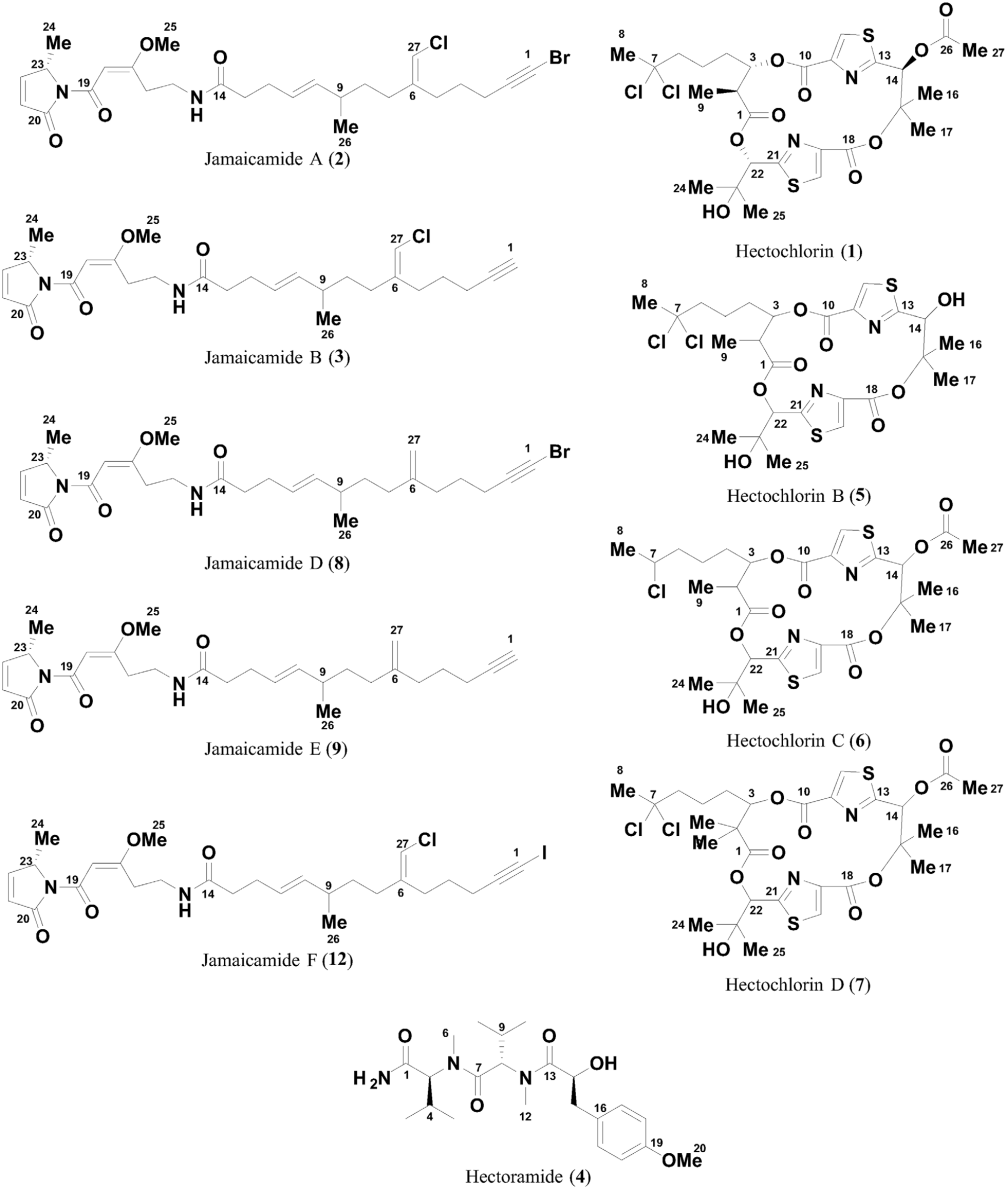
The Expanded Metabolome of *Moorea producens* JHB Described in this Study. The carbon atoms are numbered as they are in the text and supporting information.

The current study demonstrated the power of molecular networking to guide the isolation of new analogs in desired natural product families; however, the greater lesson is that no single method is comprehensive, and that to truly appreciate the secondary metabolome of an organism, it is necessary to employ orthogonal methods (Fig 9). Such an approach reveals minor or overlooked metabolites, which may be intermediates or byproducts of the biosynthetic machinery responsible for the secondary metabolome, and is applicable to complex samples, such as field collections from the environment or, as in this case, non-axenic cultures. Compounds produced in only trace quantities, however, can still be extremely valuable for understanding the biosynthetic process, for providing analogs to gain an initial appreciation of structure-activity relationships, and to provide a holistic view of the secondary metabolome of an organism.

## Materials and Methods

### General Experimental Procedures

NMR spectra were collected on a Varian Unity 500 MHz (500 MHz and 125 MHz for the ^1^H and ^13^C nuclei respectively), a Varian VX 500 MHz with ^13^C-optimized cryoprobe, and a Bruker 600 MHz (600 MHz and 150 MHz for the ^1^H and ^13^C nuclei respectively) with 1.7 mm inverse cryo-probe. NMR experiments were conducted using CDCl_3_ from Cambridge Isotope Laboratories, Inc. 99.8% D, containing 0.03% or 1.0% v/v trimethylsilane (referencing δ_H_ 0.0 as the internal standard from trimethylsilane, and δ_C_ 0.0 or δ_C_ 77.16 as internal standards using trimethylsilane and CDCl_3_, respectively). Microwave-heated reactions were run in a Biotage Initiator microwave synthesizer. LR-LCMS data were collected on a Thermo Finnigan Surveyor Autosampler/LC-Pump-Plus/PDA-Puls with a Thermo Finnigan Advantage Max mass spectrometer. HRMS data was collected on a Finnigan LTQ-FTICR-MS instrument (Thermo-Electron Corporation, San Jose, CA) fitted with either an Ion-Max ESI source for LCMS runs, or a Biversa Nanomate (Advion Biosystems, Ithaca, NY) electrospray source. HPLC purification was carried out with a Waters 515 HPLC Pump with a Waters 996 Photodiode Array Detector using Empower Pro software. All solvents were HPLC grade except for H_2_O which was purified with a Millipore Milli-Q system before use, CH_3_CN which was LCMS grade from J.T. Baker, and acetone which was distilled before use.

### Crude Extract and LCMS

*M*. *producens* JHB samples were prepared from material cultured in separate technical replicates in artificial seawater media using a 16–8 h standard light-dark cycle at 28°C. The cultures were collected by rapid vacuum filtration from the media at room temperature, combined, and extracted iteratively with 2:1 dichloromethane/methanol to afford 0.81 g crude extract. LC-HRMS data for the creation of the molecular network were collected using samples of the crude cyanobacterial extract prepared from stock solutions of the extract at 100 mg/mL in 1:1 EtOH/isooctane. Of the stock solution, 10 μL (1 mg crude extract) was diluted to 1 mg/mL in MeOH or CH_3_CN, and insoluble solids were filtered with a Celltreat PTFE syringe filter before use. Samples and a blank of CH_3_CN were run on a LC-LTQ-FTICR-MS (Liquid-column Chromatography Linear Triple Quadrupole Fourier Transform Ion Cyclotron Resonance Mass Spectrometry with a Phenomenex Synergi 4 μm Fusion-RP 80 Å 100 x 2.00 mm column with a Security Guard. The elution used a gradient that began with 70% H_2_O (acidified 1% v/v with HCOOH)/CH_3_CN for 5 min, then ramped to 1% H_2_O/CH_3_CN at 40 min, held there for 5 min, brought back 70% at 47 min, and re-equilibrated for 3 min. The flow was diverted to waste for the first 5.25 min. The MS detector used a serial set of five scans; one high-resolution FT-MS^1^ scan was followed by one low-resolution LTQ-MS^1^ scan, and then three LTQ-MS^2^ scans of the three most intense ions from the LTQ-MS^1^ scan.

### HPLC Purification

The VLC fractions containing hectochlorin (80% EtOAc/hexanes and 100% EtOAc) were combined and fractionated with a Grace Pure C18-Max 1 g/6 mL reverse phase solid phase extraction column (RP-SPE). The column was prepared with three column volumes of CH_3_CN and then run with a 30 mL elution each of CH_3_CN, MeOH, and CH_2_Cl_2_. The latter VLC fractions which contained the hectochlorin analogs (25% MeOH/EtOAc and 100% MeOH) were combined and fractionated in the same fashion. From each the CH_3_CN fraction was purified by HPLC, using a Synergi 4μ RP-80 250 x 10.00 mm column with an isocratic elution at 3.0 mL/min. The first fraction was eluted with 1.2:1.8 H_2_O/CH_3_CN for 70 min, the second with 1.25:1.75 H_2_O/CH_3_CN for 30 min. **1** was collected as 6.6 mg of a yellow-green oil, **2** as 8.9 mg of a light green oil, and **3** as 6.7 mg of a yellow-green oil; each of these matched literature reports for the NMR and HRMS spectra of these compounds [1, 2]. Also collected was **4** as 1.3 mg of a yellow oil, **6** as 0.2 mg of a yellow oil, **7** as 2.6 mg of a yellow-brown oil, and **8** as 0.5 mg of a light-green solids. Samples of **5** from the two HPLC purifications were combined and re-purified with a Jupiter 5μ C-18 300 Å 250 x 4.60 mm column eluted with 2:3 CH_3_CN/H_2_O at 1.5 mL/min and the purified **5** was collected as 1.1 mg of colorless oil.

The crude extract from the sodium iodide enrichment experiment was purified similarly with the same RP-SPE column but using an elution of 1:1 CH_3_CN/H_2_O, CH_3_CN, MeOH, and CH_2_Cl_2_. The CH_3_CN eluting fraction was purified by HPLC using a Phenomenex Kinetex 5μ C-18 100 Å 100 x 4.60 mm column, eluted with an isocratic elution of 52% CH_3_CN/H_2_O at 1.0 mL/min. The **3** was collected as 1.2 mg of a yellow oil and **12** as 1.5 mg of a faint-yellow oil.

#### Hectoramide (4)

A yellow oil; [α]^25^_D_ -11.1 (CH_2_Cl_2_, c = 3.5 mM); UV-Vis (MeOH) *λ*_maxima_ (log ε) 204 (2.(2)) nm, 262 (1.(7)) nm; IR (neat) *ν*_max_ 3396.4, 2961.8, 2929.7, 1681.1, 1629.4, 1466.9, 1249.5, 1073.9, 1036.7 cm^-1^; see Table 2 for NMR data; HR-ESI-FT-MS [M + H]^+^ *m/z* 422.2648 (calculated for C_22_H_36_N_3_O_5_ 422.2649, -0.1 mamu).

**Table 2:** NMR Data Table of Hectoramide (4).

Residue	Position	δ_C_, type^a^	δ_H_ (*J* in Hz)	HMBC^b^	TOCSY
*N*-Me-Val-1	1	171.3, C	—		
	2	62.2, CH	4.58 d (11.3)	1, 3, 5, 6	3–5
	3	25.4, CH	2.31 m	2, 5	2, 4,5
	4	18.2(8), CH_3_	0.78 d (6.7)	2, 3, 5	2, 3, 5
	5	19.6, CH_3_	1.00 d (6.4)	2–4	2–4
	6	30.8, CH_3_	3.05 s	2, 7, self	
*N*-Me-Val-2	7	171.7, C	—		
	8	58.6, CH	5.25 d (10.9)	7, 9–13	9–11
	9	27.1, CH	2.40 m	8, 10, 11	8, 10, 11
	10	18.3(4), CH_3_	0.88 d (6.9)	8, 9, 11	8, 9, 11
	11	19.4, CH_3_	0.93 d (6.4)	8–10	8–10
	12	29.7, CH_3_	2.99 s	8, 13, self	
Mpla	13	174.8, C	—		
	14	69.9, CH	4.52 bd (8.6)		OH, 15a,b
	15a	40.5, CH_2_	2.6 dd (8.8, 14.2)	13, 14, 17	14, 15b
	15b	40.5, CH_2_	2.8 dd (3.3, 14.2)	17	14, 15a
	16	129.1, C	—		
	17	130.1, CH	7.16 d (8.6)	15, 19, self	18
	18	114.0, CH	6.84 d (8.5)	16, 19, self	17
	19	158.5, C	—		
	20	55.3, CH_3_	3.79 s	19, self	
	OH	—	3.51 bs		14

^a^ Spectra collected in CDCl_3_ with 1.0% v/v TMS on a 600 MHz instrument, except for the carbon spectrum which was on an inverse 500 MHz cryo-probe.

^b^ Represents cumulative data from two HMBC experiments with differing experimental parameters (Figs AV and AW in S1 Text).

#### Hectochlorin B (5)

A faint-yellow oil; [α]^25^_D_ -0.325 (MeOH, c = 47.2 mM); UV-Vis (MeOH) *λ*_max_ (log ε) 237 (4.(0)) nm; IR (neat) *ν*_max_ 3394.2, 2976.5, 2932.2, 1715.4, 1379.8, 1323.6, 1244.5, 1150.8, 1090.5, 741.1 cm^-1^; see Table R in S1 Text for NMR data; HR-ESI-FT-MS [M + H]^+^ *m/z* 623.1050 (calculated for C_25_H_33_O_8_Cl_2_N_2_S_2_ 623.1050, 0 mamu).

#### Hectochlorin C (6)

A yellow oil; [α]^25^_D_ 4.35 (MeOH); UV-Vis (MeOH) *λ*_max_ (log ε) 232 (3.(9)) nm; IR (neat) *ν*_max_ 2926.6, 2857.7, 1715.6, 1243.7, 1211.7, 1151.1, 1085.1, 1048.3 cm^-1^; HR-ESI-FT-MS [M + H]^+^ *m/z* 631.1545 (calculated for C_27_H_36_O_9_ClN_2_S_2_ 631.1545, 0 mamu).

#### Hectochlorin D (7)

A yellow-brown oil; [α]^24^_D_ -1.53 (MeOH); UV-Vis (MeOH) *λ*_max_ (log ε) 233 (3.(4)) nm; IR (neat) *ν*_max_ 3302.8, 2928.1, 2861.1, 1712.2, 1449.0, 1242.5, 971.0 cm^-1^; HR-ESI-FT-MS [M + H]^+^ *m/z* 679.1306 (calculated for C_28_H_37_O_9_Cl_2_N_2_S_2_ 679.1312, -0.6 mamu).

#### Jamaicamide D (8)

A faint-green, amorphous solids; see Table AE in S1 Text for NMR data; HR-ESI-FT-MS [M + H]^+^ *m/z* 533.2012 (calculated for C_27_H_38_O_4_BrN_2_ 533.2009, 0.3 mamu).

#### Jamaicamide F (12)

A faint-yellow oil; [α]^25^_D_ 83.8 (CH_2_Cl_2_, c = 2.4 mM); UV-Vis (MeOH) *λ*_max_ (log ε) 266 (4.(1)) nm; IR (neat) *ν*_max_ 3318, 3116, 2933, 2863, 1716, 1656, 1599, 1545, 1439, 1396, 1335, 1296, 1204, 1171, 1137, 1082, 973, 823, and 754 cm^-1^; see Table AI in S1 Text for NMR data; HR-ESI-FT-MS [M + H]^+^ *m/z* 615.1471 (calculated for C_27_H_37_O_4_ClIN_2_ 615.1481, -1.0 mamu).

### Pure Compound HR MS/MS

Fractions from the HPLC purification were prepared for HRMS. Samples were diluted 1:10 with 50% H_2_O/MeOH acidified 1% v/v with formic acid and subjected to electrospray ionization with a Nanomate nano-spray source (pressure: 0.3–0.4 psi, spray voltage: 1.3–1.4 kV), and then fragmented and analyzed on a LTQ-FT-MS. The instrument was first auto-tuned to a standard of 100 μM cytochrome C. The [M + H]^+^ ion of each compound was isolated in the linear ion trap and fragmented by collision induced dissociation. Sets of consecutive, high-resolution MS/MS scans were acquired in profile mode and averaged using QualBrowser software by Thermo.

### Molecular Network

MS spectra were converted to Mascot generic format (.mgf) files using MSCovert (from Proteowizard [52]) and then networked with the Spectral Networking algorithm using a minimum cosine of 0.7; the data were then viewed in Cytoscape [30].

#### Preparation of Mpla Ester Standards

Samples of l and D *O*-methyl-tyrosine, 19.6 mg and 19.7 mg respectively, were charged into 8.0 mL vials and magnetic stir bars were added. These amino acids were dissolved in 1.00 mL of 0.9999(7) M NaNO_2_ (*aq*) and cooled to 0°C with stirring and 5.0 mL of 0.200(6) M perchloric acid (*aq*) was added. The reaction mixture was warmed to room temperature slowly over 30 min then sealed and heated to reflux by oil bath for 5 min. Afterwards, it was cooled to room temperature and diluted with 10 mL of 5% citric acid (*aq*) and extracted with 3 x 10 mL of CH_2_Cl_2_. The crude reaction mixture was concentrated to afford a colorless oil. This was placed in a fresh vial with a stir bar, then dissolved in 300 μL of (2*S*)-2-octanol while stirring under Ar. The acyl chloride (150 μL) was added dropwise, the vial sealed and heated to 115°C in an oil bath. After 1.5 h the vials were cooled to room temperature and quenched with 1.0 mL of water and 2.0 mL of dichloromethane. The organic layer was passed over ca. 2 cm of MgSO_4_ in a glass pipette, concentrated to afford 4.8 and 4.1 mg of the two esters as colorless oils for the l and D case, respectively.

### Stereochemical Analysis of Hectoramide (4)

A 300. μL aliquot of a 1 mg/mL solution of **4** in CH_2_Cl_2_ was transferred to a 0.5–2 mL microwave reaction tube with a stir bar, dried under N_2_ (*g*), and taken up in 600 μL of 6 M HCl (*aq*). This mixture was reacted in a microwave reactor at 110°C for 5 min, then dried again under N_2_ (*g*). The sample was redissolved in 600 μL of CH_2_Cl_2_, split into two equal portions, dried again under N_2_ (*g*), charged with a magnetic stir bar, and put under an inert Ar atmosphere.

The first sample was dissolved in 150 μL of 1 M NaHCO_3_ and 300.0 μL of D-FDAA solution (1.00 mg/mL in acetone) was added dropwise with stirring. This reaction mixture was warmed to 45–50°C for 1 h, then cooled to room temperature and neutralized with 75.0 μL of 2 M HCl (*aq*). This material was transferred to a fresh vial with 3 x 0.250 mL of CH_3_CN, concentrated to dryness under N_2_ (*g*). The sample was transferred to an LCMS vial through a syringe filter with 3 x 0.5 mL of CH_3_CN, re-concentrated under N_2_ (*g*) and brought to a final volume of 150 μL CH_3_CN. A 25.0 μL aliquot of the sample was injected on the LR-LCMS and run in comparison with previously prepared authentic standards [53]. The samples were analyzed by LR-LCMS with a Phenomenex Kinetex 5 μ C18 100 Å 100 x 4.60 mm column, and a 75 min gradient elution beginning at 5% CH_3_CN/95% H_2_O acidified with 0.1% v/v with HCOOH (Acros) for 5 min, and then ramped up to 50% CH_3_CN/50% H_2_O acidified with 0.1% v/v with formic acid over 65 min, and then brought back to the starting condition over 1 min and re-equilibrated for 4 min.

The second portion of the hydrolysate was dissolved in 200 μL of (2*S*)-2-octanol and 100 μL of acyl chloride was added dropwise with stirring. The reaction vial was sealed and warmed to 110–120°C and kept for 1 h, then cooled to room temperature and quenched with 0.50 mL of H_2_O. The reaction was extracted with 2.0 mL of CH_2_Cl_2_ and the organic layer was separated and passed through MgSO_4_. The organic layer was dried and reconstituted in a GCMS vial with 3 x 50 μL of CH_2_Cl_2_ and then analyzed by GCMS alone and in comparison with similarly derivatized D and l standards by co-injection.

### Genome sequencing

Genomic DNA from cultured biomass of *M*. *producens* JHB was extracted using a standard phenol:CHCl_3_:isoamyl alcohol extraction protocol. The genome was sequenced at the University of Michigan Microarray Core Facility (http://www.umich.edu/~caparray/), using Illumina HiSeq sequencing. Raw reads were corrected via BayesHammer [54]. Assembly was performed using the SPAdes Genome Assembler version 3.1.1 [55], followed by scaffolding with Opera and binning to obtain cyanobacterial-specific scaffolds [56], with an average G+C content of 44%, which is comparable to the related *Moorea producens* 3L genome G+C content of 44% [23]. The sequences for the clusters detailed in Fig 5 were uploaded to GenBank with the accession numbers KP860346-48.

### Media Experiments

Cultures of *Moorea producens* JHB were grown as previously described [7], in 2 L Erlyenmeyer flasks with 1 L of SWBG-11 media supplanted with equimolar amounts of NaI and NaBr at a concentration of 10 mM (1.5 g and 1.0 g into 1 L SWBG-11 media, respectively), with a control sample grown in unmodified SWBG-11 media. The inoculation was made with only a few short filaments, roughly 2–3 centimeters long. After 7 weeks of growth, the cultures were extracted and analyzed by LCMS as described above.

To provide compound **12** on a larger scale, *Moorea producens* JHB was grown in SWBG-11 media containing 10 mM NaI (15.0 g into 10 L SWBG-11). Multiple cultures were grown in shallow pans containing 5 L of media each and split into new pans with fresh media after 8 or 17 weeks of growth. A total of four pans were grown and the cyanobacteria were extracted and compounds purified as described above.

#### Neocortical Neuron Culture

Primary cultures of neocortical neurons were obtained from embryonic day 16 Swiss-Webster mice as described elsewhere [57, 58]. Briefly, pregnant mice were euthanized by CO_2_ asphyxiation and embryos were removed under sterile conditions. Neocortices were collected, stripped of meninges, minced by trituration with a Pasteur pipette, and treated with trypsin for 25 min at 37°C. The cells were then dissociated by two successive trituration and sedimentation steps in soybean trypsin inhibitor and DNase containing isolation buffer, centrifuged, and resuspended in Eagle’s minimal essential medium with Earle’s salt (MEM) and supplemented with 1 mM l-glutamine, 10% fetal bovine serum, 10% horse serum, 100 IU/mL penicillin, and 0.10 mg/mL streptomycin (pH 7.4). Cells were plated onto poly-l-lysine-coated 96-well (9 mm), clear-bottomed, black-well culture plates (MidSci, St. Louis, USA) at a density of 1.5 × 10^5^ cells/well. Cells were then incubated at 37°C in a 5% CO_2_ and 95% humidity atmosphere. The culture media was changed every other day, starting from day 5 *in-vitro* using a serum-free growth medium containing Neurobasal Medium supplemented with B-27, 100 IU/mL penicillin, 0.10 mg/mL streptomycin, and 0.2 mM l-glutamine. Neocortical cultures were used in experiments between 10–11 days *in-vitro*. All animal use protocols were approved by the Institutional Animal Care and Use Committee (IACUC) of Creighton University.

#### Intracellular Ca^2+^ Concentration Measurement

Cells grown in 96-well plates were used for determination of intracellular Ca^2+^ concentration ([Ca^2+^]_i_). Briefly, the growth medium was removed and replaced with dye-loading medium (100 μL per well) containing 4 μM fluo-3 AM and 0.04% pluronic acid in Locke's buffer. After 1 h of incubation in dye-loading medium, the cells were washed four times in fresh Locke's buffer (8.6 mM HEPES, 5.6 mM KCl, 154 mM NaCl, 5.6 mM glucose, 1.0 mM MgCl_2_, 2.3 mM CaCl_2_, 0.1 mM glycine, pH 7.4) using an automated microplate washer (Bio-Tek Instruments Inc., VT, USA). Various concentrations of **2**, **3**, and **12** were then added to the cells at a rate of 26 μL/s, yielding a final volume of 200 μL/well. The cells were incubated in the presence or absence of jamaicamides for 5–7 minutes at 37°C in a 5% CO_2_ and 95% humidity atmosphere prior to transfer to a FLEX Station II (Molecular Devices) benchtop scanning fluorescence chamber. The fluorescence measurements were performed at 37°C. The cells were excited at 488 nm and Ca^2+^-bound fluo-3 emission was recorded at 538 nm at 2 s intervals. After recording baseline fluorescence for 60 s, either vehicle or veratridine was added to each well at a rate of 26 μL/s; the fluorescence was then monitored for an additional 200 s.

#### Intracellular Na^+^ Concentration Measurement

[Na^+^]_i_ measurement and full *in-situ* calibration of sodium-binding benzofuran isophthalate (SBFI) fluorescence ratio were performed as described previously [58]. Cells grown in 96-well plates were washed four times with Locke's buffer using an automated microplate washer (BioTek Instruments, Winooski, VT). After measuring the background fluorescence of each well, cells were incubated for 1 h at 37°C with dye-loading buffer (100 *μ*L/well) containing 10 *μ*M SBFI-AM (Invitrogen) and 0.02% Pluronic F-127 (Invitrogen). Cells were washed five times with Locke's buffer, leaving a final volume of 150 *μ*L in each well. Jamaicamide addition and preincubation was as described above for Ca^2+^ concentration determination. Plates were then placed in a FlexStation II (Molecular Devices, Sunnyvale, CA) chamber to detect Na^+^-bound SBFI emission at 505 nm (excitation wavelength was 340 and 380 nm). Fluorescence readings were taken once every 5 s for 60 s to establish the baseline, then either vehicle (control) or veratridine was added and fluorescence was monitored for an additional 260 s. After correcting for background fluorescence, SBFI fluorescence ratios (340/380) and concentration-response graphs were generated.

#### Bioassay Data Analysis

The Fluo-3 and SBFI raw fluorescence emission data were exported to an Excel work sheet. The Fluo-3 fluorescence or SBFI fluorescence ratios (340/380) versus time were analyzed and concentration-response graphs generated using GraphPad Prism software (GraphPad Software Inc., San Diego, CA). The IC_**50**_ values of **2**, **3**, and **12** for antagonism of veratridine-stimulated calcium or sodium influx were determined by nonlinear regression analysis using a three-parameter logistic equation.

## Supporting Information

S1 Text Supporting Information Text File. Details of experimental results.
(PDF)
