# Supplementary material for: Expanding the Described Metabolome of the Marine Cyanobacterium Moorea producens JHB through Orthogonal Natural Products Workflows
Source: PLoS One. 2015 Jul 29;10(7):e0133297. doi: 10.1371/journal.pone.0133297 (PMC4519256; doi:10.1371/journal.pone.0133297)
Supplement: S1 Text — Details of experimental results. (PDF) [file pone.0133297.s001.pdf]

Expanding the Described Metabolome of the Marine Cyanobacterium *Moorea producens* JHB through Orthogonal Natural Products Workflows

Paul D. Boudreau[1], Emily A. Monroe[2], Suneet Mehrotra[3], Shane Desfor[1,4], Anton Korobeynikov[5,6,7], David H. Sherman[8], Thomas F. Murray[3], Lena Gerwick[1], Pieter C. Dorrestein[9,10], and William H. Gerwick[1,10]*

[1]Center for Marine Biotechnology and Biomedicine, Scripps Institution of Oceanography, University of California San Diego, La Jolla, California 92093, United States

[2] Department of Biology, William Paterson University, Wayne, New Jersey, 07470, United States of America

[3]Department of Pharmacology, Creighton University School of Medicine, Omaha, Nebraska 68178, United States of America

[4]Department of Biology, California State University, San Marcos, San Marcos, California, 92078, United States of America

[5]Algorithmic Biology Laboratory, St. Petersburg Academic University, Russian Academy of Sciences, St. Petersburg, Russia

[6]Department of Mathematics and Mechanics, St. Petersburg State University, St. Petersburg, Russia

[7]Center for Algorithmic Biotechnology, St. Petersburg State University, St. Petersburg, Russia

[8]Life Sciences Institute and Department of Medical Chemistry, University of Michigan, Ann Arbor, Michigan, United States of America

[9]Department of Chemistry and Biochemistry, University of California San Diego, La Jolla, California 92093, United States of America

[10]Skaggs School of Pharmacy and Pharmaceutical Sciences, University of California San Diego, La Jolla, California 92093, United States of America

* Corresponding author

E-mail: wgerwick@ucsd.edu

The *M. producens* JHB Molecular Network

Figure A Full JHB molecular network from LTQ-FT data.
Figure B Jamaicamides cluster within the JHB molecular network from LTQ-FT data.
Figure C IT-MS^2 fragment spectra for the jamaicamides from LTQ-FT data.
Figure D Hectochlorins cluster within the JHB molecular network from LTQ-FT data.
Figure E IT-MS^2 fragment spectra for the hectochlorins from LTQ-FT data.
Table F $HRMS^1$ Masses of observed for the hectochlorins and jamaicamides from LTQ-FT data.
Figure G Full JHB molecular network, iodide rich media, from LCQ data.
Figure H Jamaicamides cluster within the JHB molecular network, iodide rich media, from LCQ data.
Figure I IT-MS^2 fragment spectra for the jamaicamides, iodide rich media, from LCQ data.

Spectroscopic Data for Hectochlorin (1)

Table J NMR data summary for hectochlorin (**1**).
Figure K ^{1}H-NMR (600 MHz, $CDCl_3$) spectrum of hectochlorin (**1**).
Figure L ^{13}C-NMR (125 MHz, $CDCl_3$) spectrum of hectochlorin (**1**).
Figure M HSQC-NMR (600 MHz, $CDCl_3$) spectrum of hectochlorin (**1**).
Figure N HMBC-NMR (600 MHz, $CDCl_3$) spectrum of hectochlorin (**1**).
Figure O TOCSY-NMR (600 MHz, $CDCl_3$) spectrum of hectochlorin (**1**).
Figure P FT-MS^2 Fragment spectrum of hectochlorin (**1**).
Figure Q FT-MS^2 Fragment summary for hectochlorin (**1**).

Spectroscopic Data for Hectochlorin B (5)

Table R NMR data summary for hectochlorin B (**5**).
Figure S ^{1}H-NMR (600 MHz, $CDCl_3$) spectrum of hectochlorin B (**5**).
Figure T ^{13}C-NMR (125 MHz, $CDCl_3$) spectrum of hectochlorin B (**5**).
Figure U HSQC-NMR (600 MHz, $CDCl_3$) spectrum of hectochlorin B (**5**).
Figure V HMBC-NMR (600 MHz, $CDCl_3$) spectrum of hectochlorin B (**5**).
Figure W TOCSY-NMR (600 MHz, $CDCl_3$) spectrum of hectochlorin B (**5**).
Figure X FT-MS^2 Fragment spectrum of hectochlorin B (**5**).
Figure Y FT-MS^2 Fragment summary for hectochlorin B (**5**).

Spectroscopic Data for Hectochlorin C (6)

Figure Z FT-MS^2 Fragment spectrum of hectochlorin C (**6**).
Figure AA FT-MS^2 Fragment summary for hectochlorin C (**6**).

Spectroscopic Data for Hectochlorin D (7)

Figure AB FT-MS^2 Fragment spectrum of hectochlorin D (**7**).

Figure AC FT-MS^2 Fragment summary for hectochlorin D (**7**).

Biosynthetic Pathway Divergence for Jamaiacmide A and D (2 and 8)

Figure AD Proposed Biosynthetic Pathway Divergence for Jamaicamide A and D (**2** and **8**).

Spectroscopic Data for Jamaicamide D (8)

Table AE NMR data summary for jamaicamide D (**8**).
Figure AF ^{1}H-NMR (500 MHz, $CDCl_3$) spectrum of jamaicamide D (**8**).
Figure AG HMBC-NMR (500 MHz, $CDCl_3$) spectrum of jamaicamide D (**8**).
Figure AH TOCSY-NMR (500 MHz, $CDCl_3$) spectrum of jamaicamide D (**8**).

Spectroscopic Data for Jamaicamide F (12)

Table AI NMR data summary for jamaicamide F (**12**).
Figure AJ ^{1}H-NMR (600 MHz, $CDCl_3$) spectrum of jamaicamide F (**12**).
Figure AK ^{13}C-NMR (125 MHz, $CDCl_3$) spectrum of jamaicamide F (**12**).
Figure AL HSQC-NMR (600 MHz, $CDCl_3$) spectrum of jamaicamide F (**12**).
Figure AM HMBC-NMR (600 MHz, $CDCl_3$) spectrum of jamaicamide F (**12**).
Figure AN TOCSY-NMR (600 MHz, $CDCl_3$) spectrum of jamaicamide F (**12**).
Figure AO MS^2 fragment spectra of jamaicamide F (**12**).

Bioassay Data for Jamaicamides A, B, and F (2,3, and 12)

Figure AP Effect of the jamaicamides on the veratridine-induced Ca^{2+} influx in murine neocortical neurons.
Figure AQ Effect of the jamaicamides on the veratridine-induced Na^+ influx in murine neocortical neurons.

Spectroscopic Data for Hectoramide (4)

Figure AR ^{1}H-NMR (600 MHz, $CDCl_3$) spectrum of hectoramide (**4**).
Figure AS ^{13}C-NMR (600 MHz, $CDCl_3$) spectrum of hectoramide (**4**).
Figure AT Predicted ^{13}C-NMR shifts for potential structures of hectoramide (**4**).
Figure AU HSQC (600 MHz, $CDCl_3$) spectrum of hectoramide (**4**).
Figure AV HMBC (600 MHz, $CDCl_3$) spectrum of hectoramide (**4**), long duration.
Figure AW HMBC (600 MHz, $CDCl_3$) spectrum of hectoramide (**4**), short duration.
Figure AX TOCSY (600 MHz, $CDCl_3$) spectrum of hectoramide (**4**).
Figure AY GC-MS analysis of 2-S-octonol ester standards.
Figure AZ GC-MS analysis of 2-S-octonol ester derivatized hydrolysate of hectoramide (**4**).
Figure AAA Marfey's analysis of D-FDAA derivatized hydrolysate of hectoramide (**4**).

Figure A. Full *Moorea producens* JHB Molecular Network, from LTQ-FT Data.

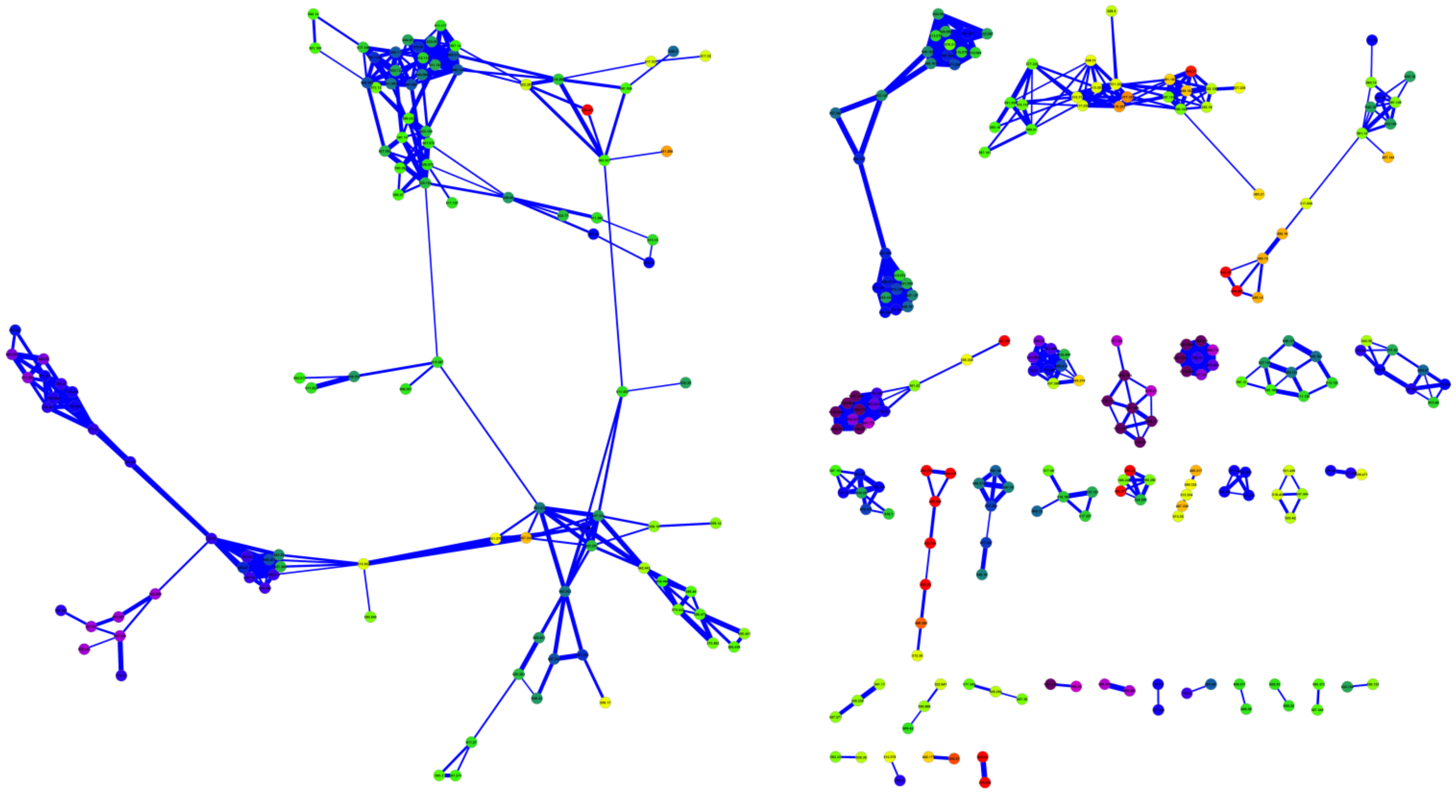

Figure B. Hectochlorin Cluster within the JHB Molecular Network, from LTQ-FT Data.

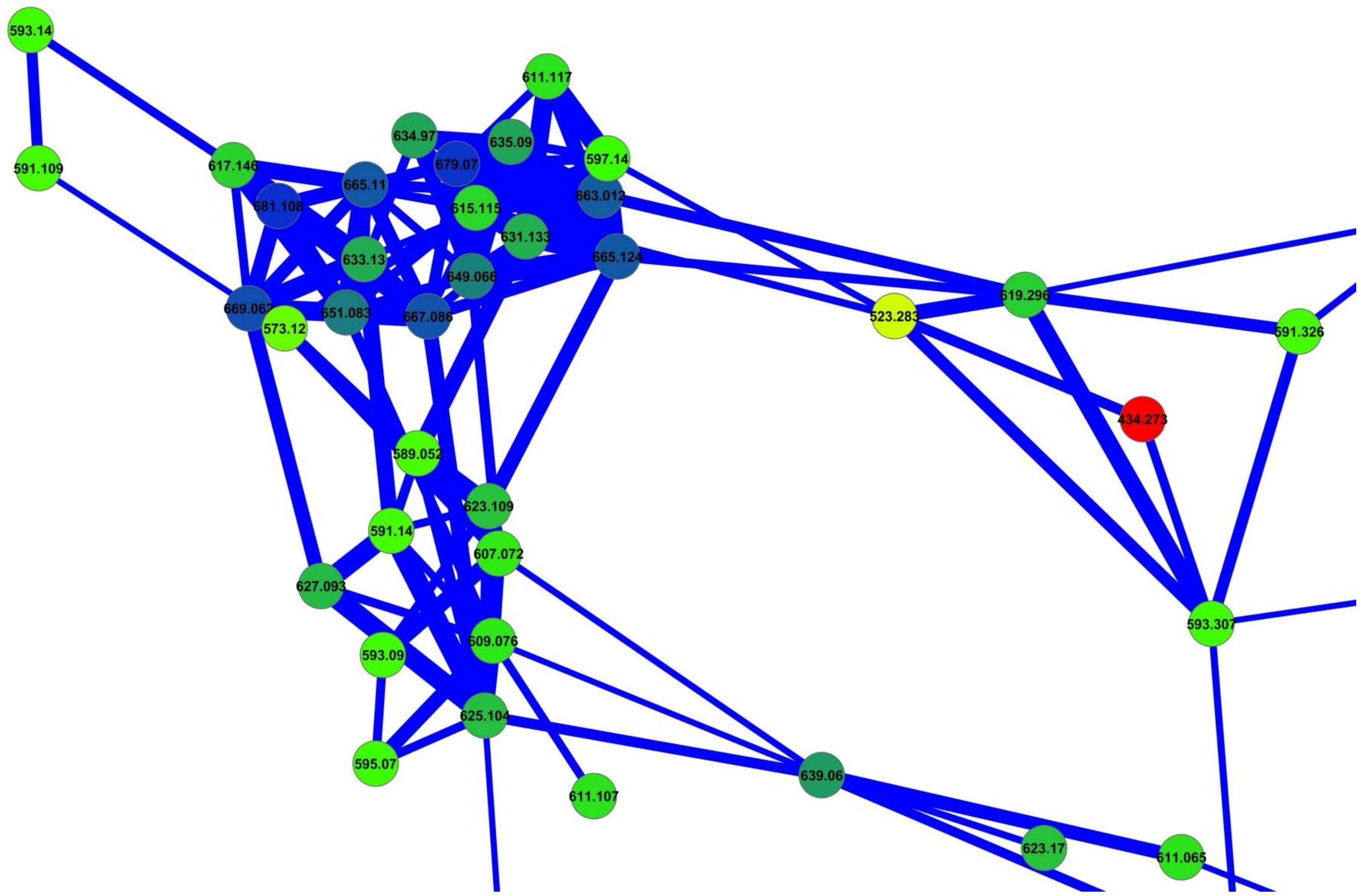

Figure C. IT-MS^2 Fragment Spectra for the Hectochlorins, from LTQ-FT Data.

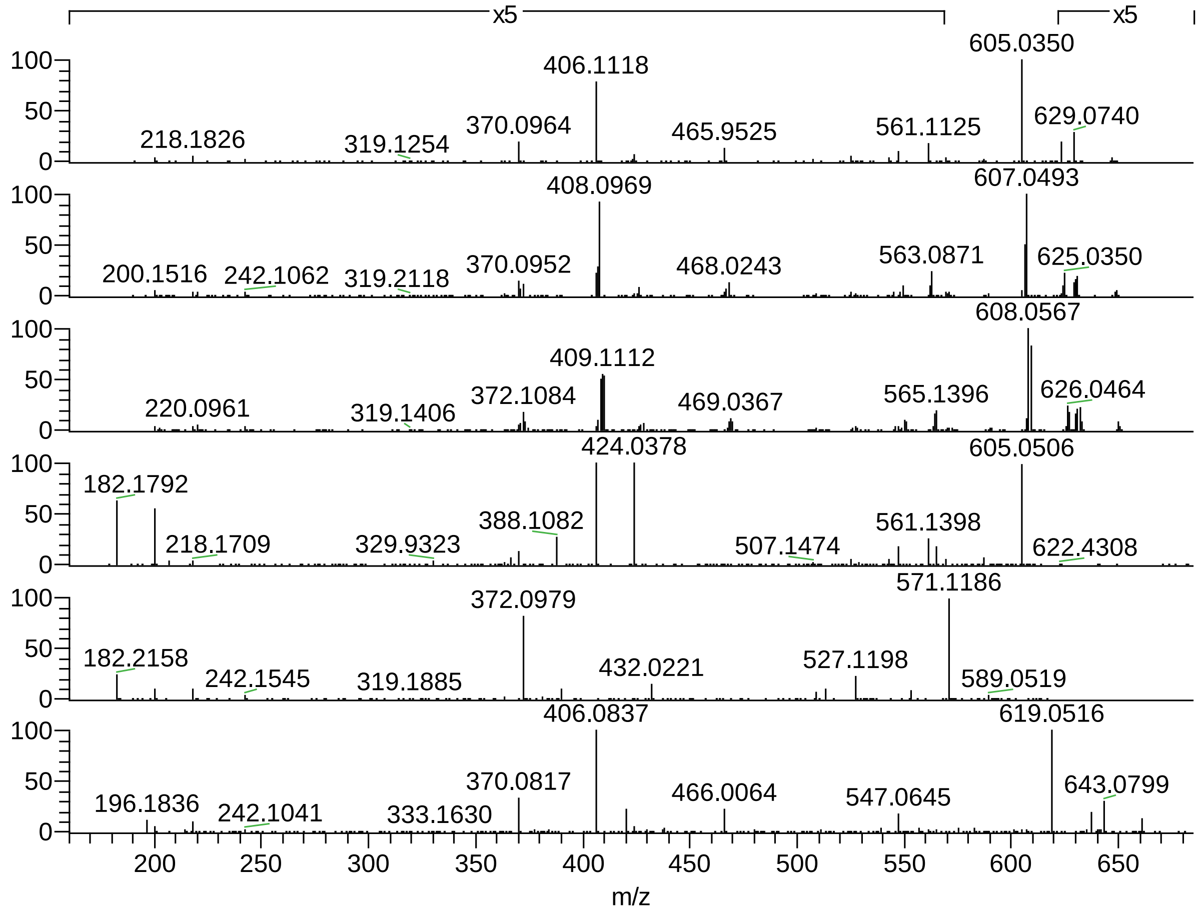

Fragment Spectra:

Hectochlorin (**1**) $[M + H]^+$, Parent Mass 665 *m/z*

Hectochlorin (**1**) $[M^{37}Cl + H]^+$, Parent Mass 667 *m/z*

Hectochlorin (**1**) $[M^{37}Cl_2 + H]^+$, Parent Mass 669 *m/z*

Hectochlorin B (**5**) $[M + H]^+$, Parent Mass 623 *m/z*

Hectochlorin C (**6**) $[M + H]^+$, Parent Mass 631 *m/z*

Hectochlorin D (**7**) $[M + H]^+$, Parent Mass 679 *m/z*

Figure D. Jamaicamide Cluster within the JHB Molecular Network, from LTQ-FT Data.

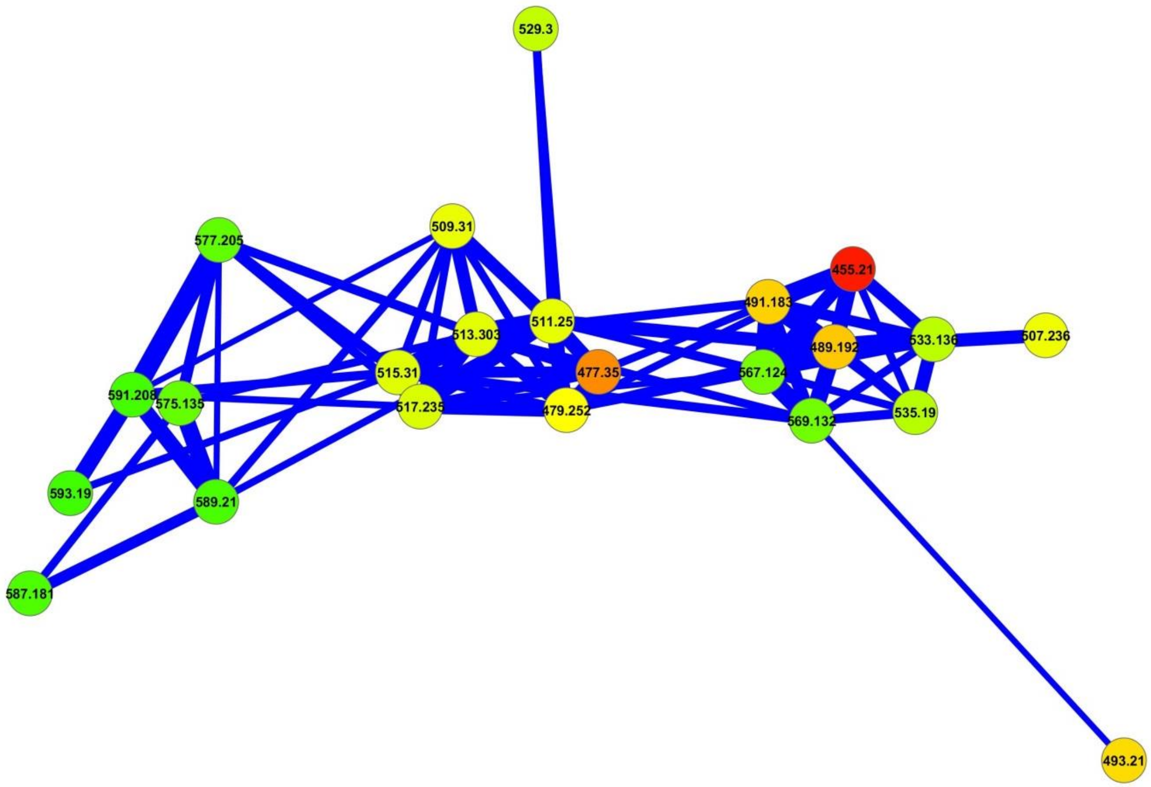

Figure E. IT-MS^2 Fragment Spectra for the Jamaicamides, from LTQ-FT Data.

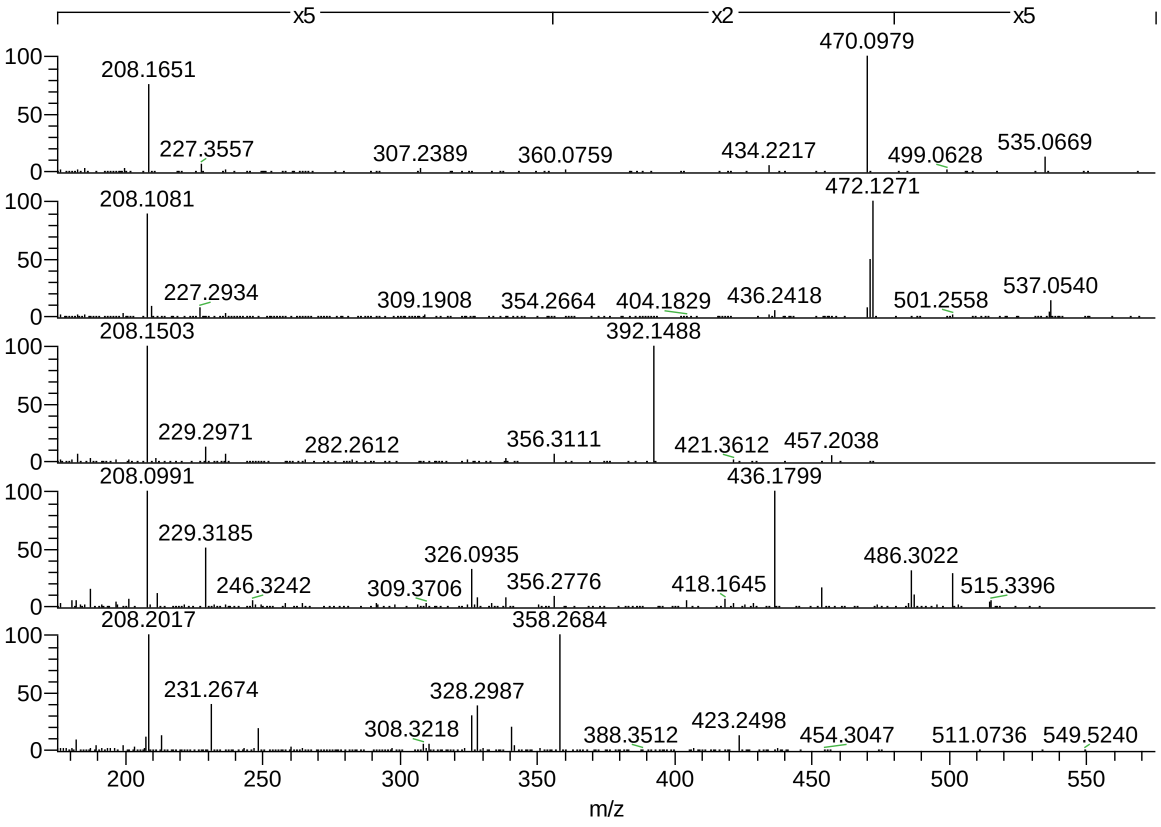

Fragment Spectra:

Jamaicamide A (**2**) $[M + H]^+$, Parent Mass 567 *m/z*

Jamaicamide A (**2**) $[M^{81}Br + H]^+$, Parent Mass 569 *m/z*

Jamaicamide B (**3**) $[M + H]^+$, Parent Mass 489 *m/z*

Jamaicamide D (**8**) $[M + H]^+$, Parent Mass 533 *m/z*

Jamaicamide E (**9**) $[M + H]^+$, Parent Mass 455 *m/z*

Table F. HRMS[1] Masses of Observed for the Hectochlorins and Jamaicamides, from LTQ-FT Data.

Compound	Calculated Formula	LCMS Observed HRMS[1] Mass *m/z*	Retention Time (min)	Isolated Compound HRMS[1] Mass *m/z*
Hectochlorin (**1**)	$C_{27}H_{35}Cl_2N_2O_9S_2$ $[M+H]^+$, 665.1156<br>$C_{27}H_{35}Cl^{37}ClN_2O_9S_2$ $[M+H]^+$, 667.1126<br>$C_{27}H_{34}Cl_2N_2NaO_9S_2$ $[M+Na]^+$, 687.0975<br>$C_{27}H_{34}Cl^{37}ClN_2NaO_9S_2$ $[M+Na]^+$, 689.0945	665.1182, 3.9 ppm, 2.6 mamu<br>667.1153, 4.0 ppm, 2.7 mamu<br>687.1070, 13.8 ppm, 9.5 mamu<br>689.1057, 16.2 ppm, 11.2 mamu	23.8	665.1154, -0.3 ppm, -0.2 mamu<br>667.1126, 0 ppm, 0 mamu<br>687.0976, 0.1 ppm, 0.1 mamu<br>689.0948, 0.4 ppm, 0.3 mamu
Hectochlorin B (**5**)	$C_{25}H_{33}Cl_2N_2O_8S_2$ $[M+H]^+$, 623.1050<br>$C_{25}H_{33}Cl^{37}ClN_2O_8S_2$ $[M+H]^+$, 625.1020<br>$C_{25}H_{33}Cl_2N_2NaO_8S_2$ $[M+Na]^+$, 645.0869<br>$C_{25}H_{33}Cl^{37}ClN_2NaO_8S_2$ $[M+Na]^+$, 647.0840	623.1041, -1.4 ppm, -0.9 mamu<br>625.1019, -0.2 ppm, -0.1 mamu<br>645.0884, 2.3 ppm, 1.5 mamu<br>647.0856, 2.5 ppm, 1.6 mamu	20.3	623.1050, 0 ppm, 0 mamu<br>625.1021, 0.2 ppm, 0.1 mamu<br>645.0870, 0.2 ppm, 0.1 mamu<br>647.0841, 0.2 ppm, 0.1 mamu
Hectochlorin C (**6**)	$C_{27}H_{36}ClN_2O_9S_2$ $[M+H]^+$, 631.1545<br>$C_{27}H_{36}{}^{37}ClN_2O_9S_2$ $[M+H]^+$, 633.1516<br>$C_{27}H_{36}ClN_2NaO_9S_2$ $[M+Na]^+$, 653.1365<br>$C_{27}H_{36}{}^{37}ClN_2NaO_9S_2$ $[M+Na]^+$, 655.1335	631.1516, -4.6 ppm, -2.9 mamu<br>633.1492, -3.8 ppm, -2.4 mamu<br>653.1333, -4.9 ppm, -3.2 mamu<br>655.1312, -3.5 ppm, -2.3 mamu	22.6	631.1545, 0.0 ppm, 0 mamu<br>633.1519, 0.5 ppm, 0.3 mamu<br>653.1360, -0.8 ppm, 0.5 mamu<br>655.1337, 0.3 ppm, 0.2 mamu
Hectochlorin D (**7**)	$C_{28}H_{37}Cl_2N_2O_9S_2$ $[M+H]^+$, 679.1312<br>$C_{28}H_{37}Cl^{37}ClN_2O_9S_2$ $[M+H]^+$, 681.1283<br>$C_{28}H_{37}Cl_2N_2NaO_9S_2$ $[M+Na]^+$, 701.1131<br>$C_{28}H_{37}Cl^{37}ClN_2NaO_9S_2$ $[M+Na]^+$, 703.1102	679.1273, -5.7 ppm, -3.9 mamu<br>681.1245, -5.8 ppm, -3.8 mamu<br>701.1091, -5.7 ppm, -4.0 mamu<br>703.1065, -5.3 ppm, -3.7 mamu	25.8	679.1306, -0.9 ppm, -0.6 mamu<br>681.1274, -1.3 ppm, -0.9 mamu<br>701.1129, -0.3 ppm, -0.2 mamu<br>703.1097, -0.7 ppm, -0.5 mamu
Jamaicamide A (**2**)	$C_{27}H_{37}BrClN_2O_4$ $[M+H]^+$, 567.1620<br>$C_{27}H_{37}{}^{81}BrClN_2O_4$ $[M+H]^+$, 569.1599<br>$C_{27}H_{36}BrClN_2NaO_4$ $[M+Na]^+$, 589.1439<br>$C_{27}H_{36}{}^{81}BrClN_2NaO_4$ $[M+Na]^+$, 591.1419	567.1631, 1.9 ppm, 1.1 mamu<br>569.1601, 0.3 ppm, 0.2 mamu<br>589.1478, 6.6 ppm, 3.9 mamu<br>591.1450, 5.2 ppm, 3.1 mamu	30.5	567.1621, 0.2 ppm, 0.1 mamu<br>569.1597, -0.4 ppm, -0.2 mamu<br>589.1442, 0.5 ppm, 0.3 mamu<br>591.1418, -0.2 ppm, -0.1 mamu
Jamaicamide B (**3**)	$C_{27}H_{38}ClN_2O_4$ $[M+H]^+$, 489.2515<br>$C_{27}H_{38}{}^{37}ClN_2O_4$ $[M+H]^+$, 491.2485<br>$C_{27}H_{37}ClN_2NaO_4$ $[M+Na]^+$, 511.2334<br>$C_{27}H_{37}{}^{37}ClN_2NaO_4$ $[M+Na]^+$, 513.2305	489.2515, 0 ppm, 0 mamu<br>491.2510, 5.1 ppm, 2.5 mamu<br>511.2358, 4.7 ppm, 2.4 mamu<br>513.2336, 6.0 ppm, 3.1 mamu	27.2	489.2513, -0.4 ppm, -0.2 mamu<br>491.2487, 0.4 ppm, 0.2 mamu<br>511.2333, -0.2 ppm, 0.1 mamu<br>513.2306, 0.2 ppm, 0.1 mamu
Jamaicamide D (**8**)	$C_{27}H_{38}BrN_2O_4$ $[M+H]^+$, 533.2009<br>$C_{27}H_{38}{}^{81}BrN_2O_4$ $[M+H]^+$, 535.1989<br>$C_{27}H_{37}BrN_2NaO_4$ $[M+Na]^+$, 555.1829<br>$C_{27}H_{37}{}^{81}BrN_2NaO_4$ $[M+Na]^+$, 557.1808	533.1983, -4.9 ppm, -2.6 mamu<br>535.1962, -5.0 ppm, -2.7 mamu<br>555.1799, -5.4 ppm, -3.0 mamu<br>557.1778, -5.4 ppm, -3.0 mamu	29.9	533.2012, 0.6 ppm, 0.3 mamu<br>535.1991, 0.4 ppm, 0.2 mamu<br>555.1833, 0.7 ppm, 0.4 mamu<br>557.1812, 0.7 ppm, 0.4 mamu
Jamaicamide E (**9**)	$C_{27}H_{39}N_2O_4$ $[M+H]^+$, 455.2904<br>$C_{27}H_{38}N_2NaO_4$ $[M+Na]^+$, 477.2724	455.2877, -5.9 ppm, -2.7 mamu<br>477.2693, -6.5 ppm, -3.1 mamu	26.5	Not Isolated by HPLC
Jamaicamide F (**12**)	$C_{27}H_{37}ClIN_2O_4$ $[M+H]^+$, 615.1481<br>$C_{27}H_{37}{}^{37}ClIN_2O_4$ $[M+H]^+$, 617.1452<br>$C_{27}H_{36}ClIN_2NaO_4$ $[M+Na]^+$, 637.1300<br>$C_{27}H_{36}{}^{37}ClIN_2NaO_4$ $[M+Na]^+$, 639.1271	615.1549, 11.1 ppm, 6.8 mamu<br>617.1516, 10.4 ppm, 6.4 mamu<br>637.1363, 9.9 ppm, 6.3 mamu<br>639.1333, 9.7 ppm, 6.2 mamu	29.4	615.1471, -1.6 ppm, -1.0 mamu<br>617.1441, -1.8 ppm, -1.1 mamu<br>637.1289, -1.7 ppm, -1.1 mamu<br>639.1259, -1.9 ppm, -1.2 mamu

Figure G. Full JHB Molecular Network, Iodide Rich Media, from LCQ Data.

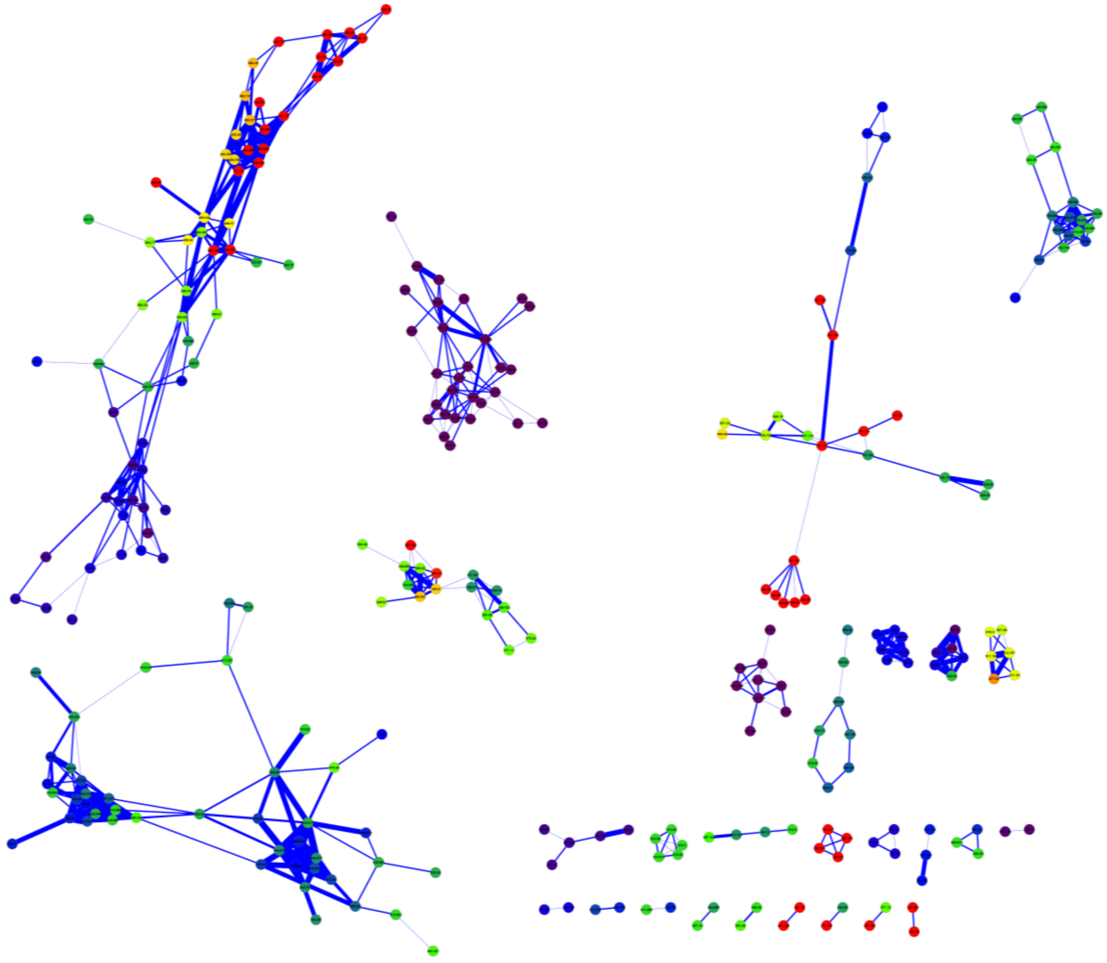

Figure H. Jamaicamides Cluster within the JHB Molecular Network, Iodide Rich Media, from LCQ Data.

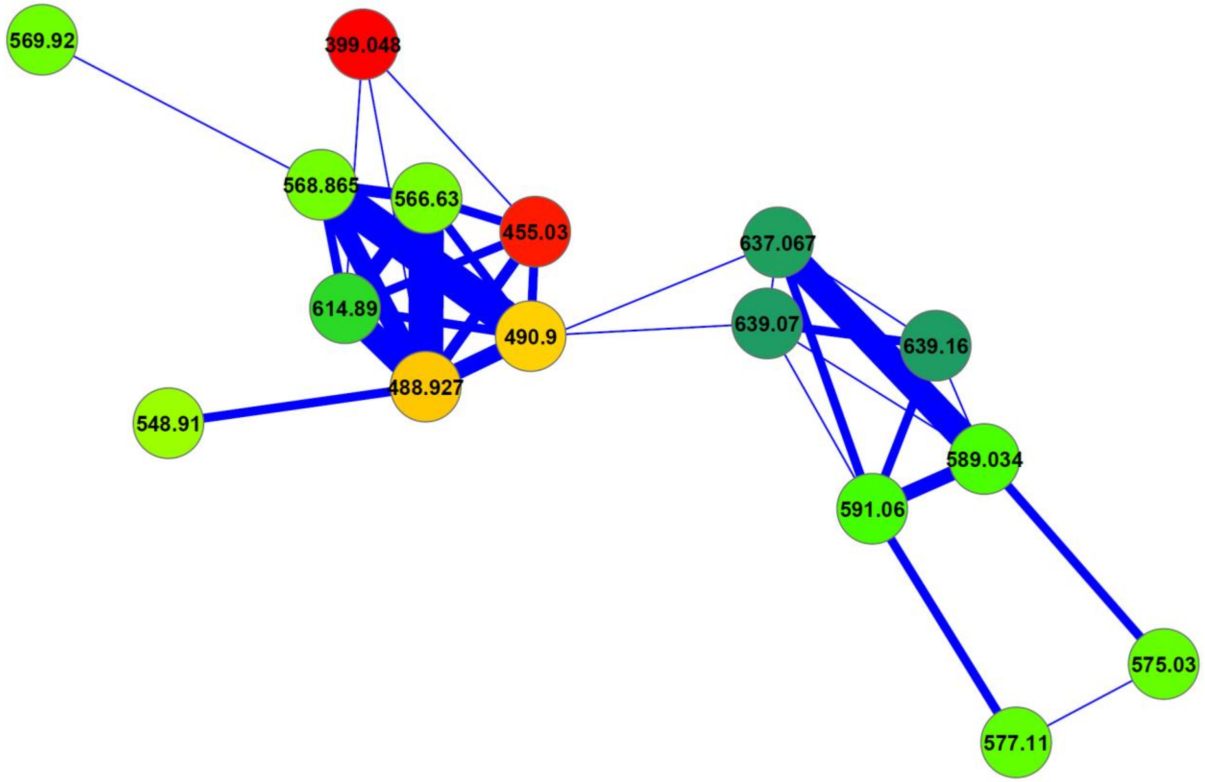

Figure I. IT-MS^2 Fragment Spectra for the Jamaicamides, Iodide Rich Media, from LCQ Data.

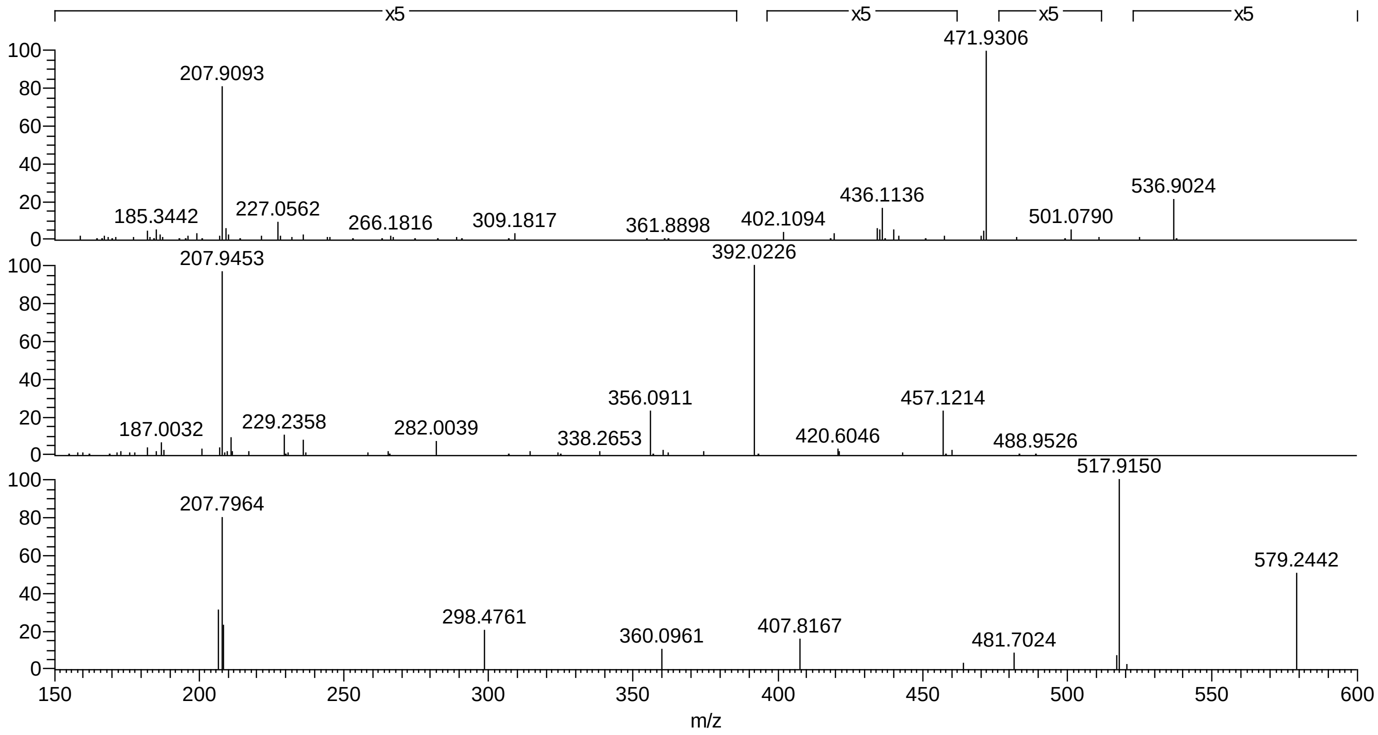

Fragment Spectra:

Jamaicamide A (**2**)
[M^{81}Br + H]$^+$,
Parent Mass 569 *m/z*

Jamaicamide B (**3**)
[M + H]$^+$,
Parent Mass 489 *m/z*

Jamaicamide F (**12**)
[M + H]$^+$,
Parent Mass 615 *m/z*

Table J. NMR Data Summary for Hectochlorin (1).

Position	δ_C, type[a]	δ_H (J in Hz)[b]	HMBC[b]	TOCSY[b]
1	172.9, C	--		
2	42.6, CH	3.16, quintet (7.4)	1, 3, 9	3-6, 9
3	75.2, CH	5.35, m		2, 4-6, 9
4a	30.9, CH_2	1.71, m		2, 3, 4b, 5, 6, 9
4b	30.9, CH_2	1.86, m		2, 3, 4a, 5, 6, 9
5	20.9, CH_2	1.71, m		2-4, 6, 9
6a	49.3, CH_2	2.26, m		2-5, 6b, 9
6b	49.3, CH_2	2.15, m		2-5, 6a, 9
7	90.4, C	--		
8	37.2, CH_3	2.11, s	6,7	
9	15.1, CH_3	1.29, d (7.4)	1-3, self	2-6
10	161.1, C	--		
11	147.0, C	--		
12	128.6, CH	8.17, s	11, 13	14
13	166.3, C[c]	--		
14	74.7, CH	6.84, s	13, 15-17, 26	12
15	82.0, C	--		
16	24.4, CH_3	1.84, s	14, 15, 17, self	
17	21.9, CH_3	1.61, s	14-16, self	
18	160.4, C	--		
19	147.5, C	--		
20	127.7, CH	7.94, s	19, 21	
21	165.1, C	--		
22	77.8, CH	5.67, s	1, 21, 23, 25	
23	71.6, C	--		
24	26.7, CH_3	1.31, s	22, 23, 25	
25	26.0, CH_3	1.37, s	22, 24	
26	168.7, C	--		
27	20.9, CH_3	2.19, s	26, self	

All experiments in $CDCl_3$ with TMS standard, 1% v/v for 13C experiment, and 0.3% v/v for the rest. [a]Specturm collected on a Varian VX 500 MHz with ^{13}C-optimized cryoprobe. [b]Spectra collected on a Bruker 600 MHz (600 MHz and 150 MHz for the ^{1}H and ^{13}C nuclei respectively) with 1.7 mm inverse cryo-probe. [c]Signal by projection from the HMBC experiment.

Figure K. ^{1}H-NMR (600 MHz, $CDCl_3$) Spectrum of Hectochlorin (1).

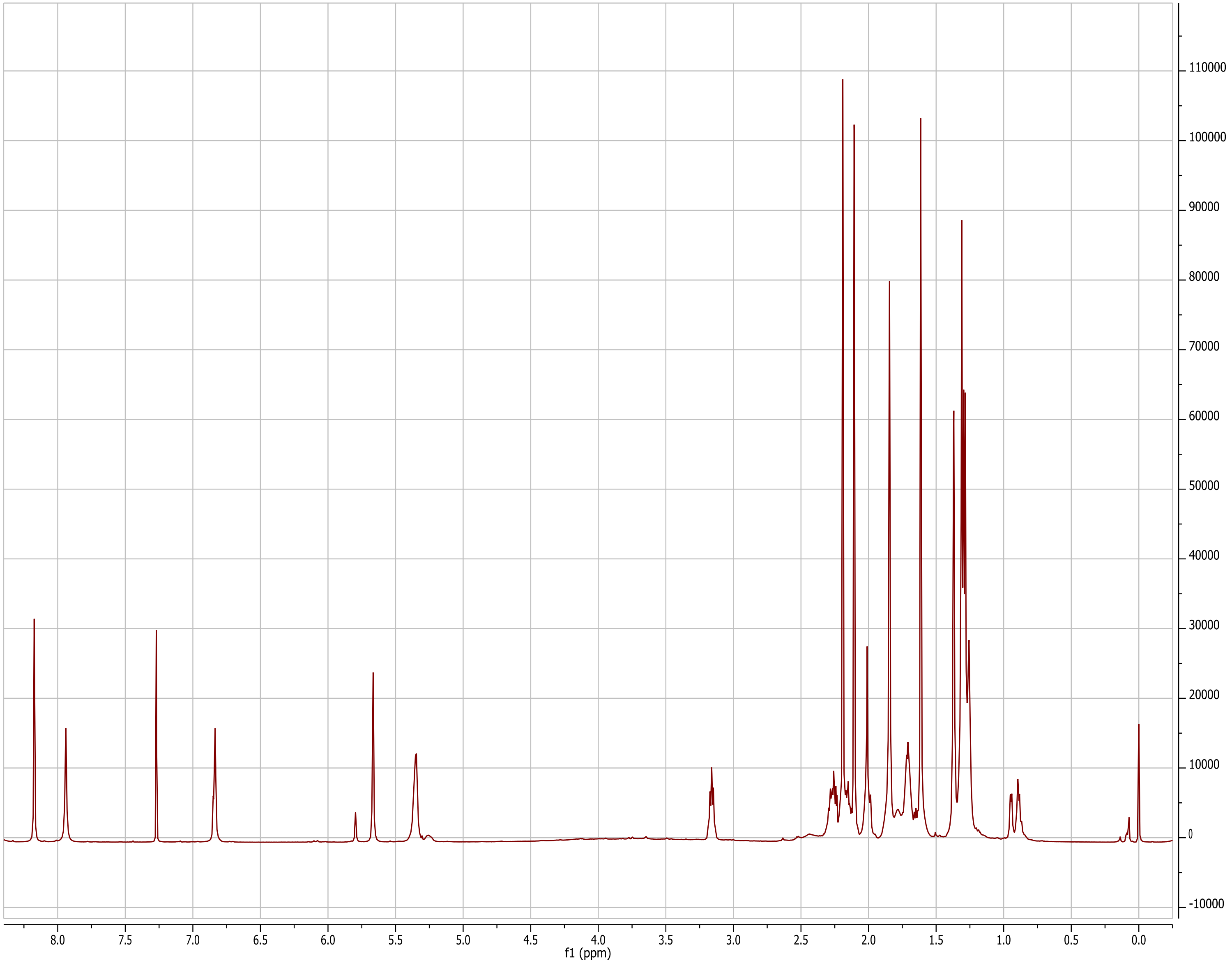

Figure L. ^{13}**C-NMR (125 MHz, $CDCl_3$) Spectrum of Hectochlorin (1).**

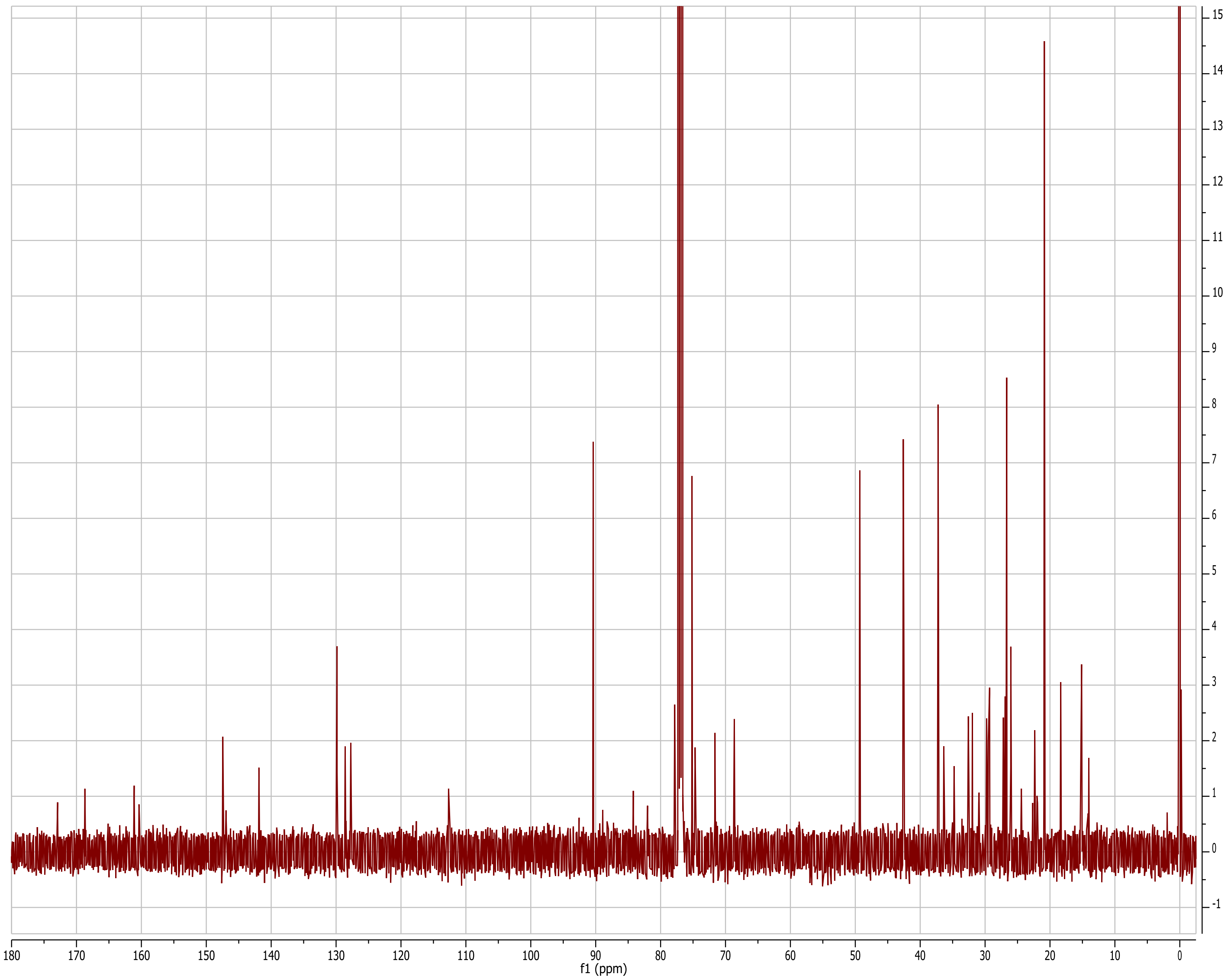

Benzene contamination at 129.8 ppm.

Figure M. HSQC-NMR (600 MHz, $CDCl_3$) Spectrum of Hectochlorin (1).

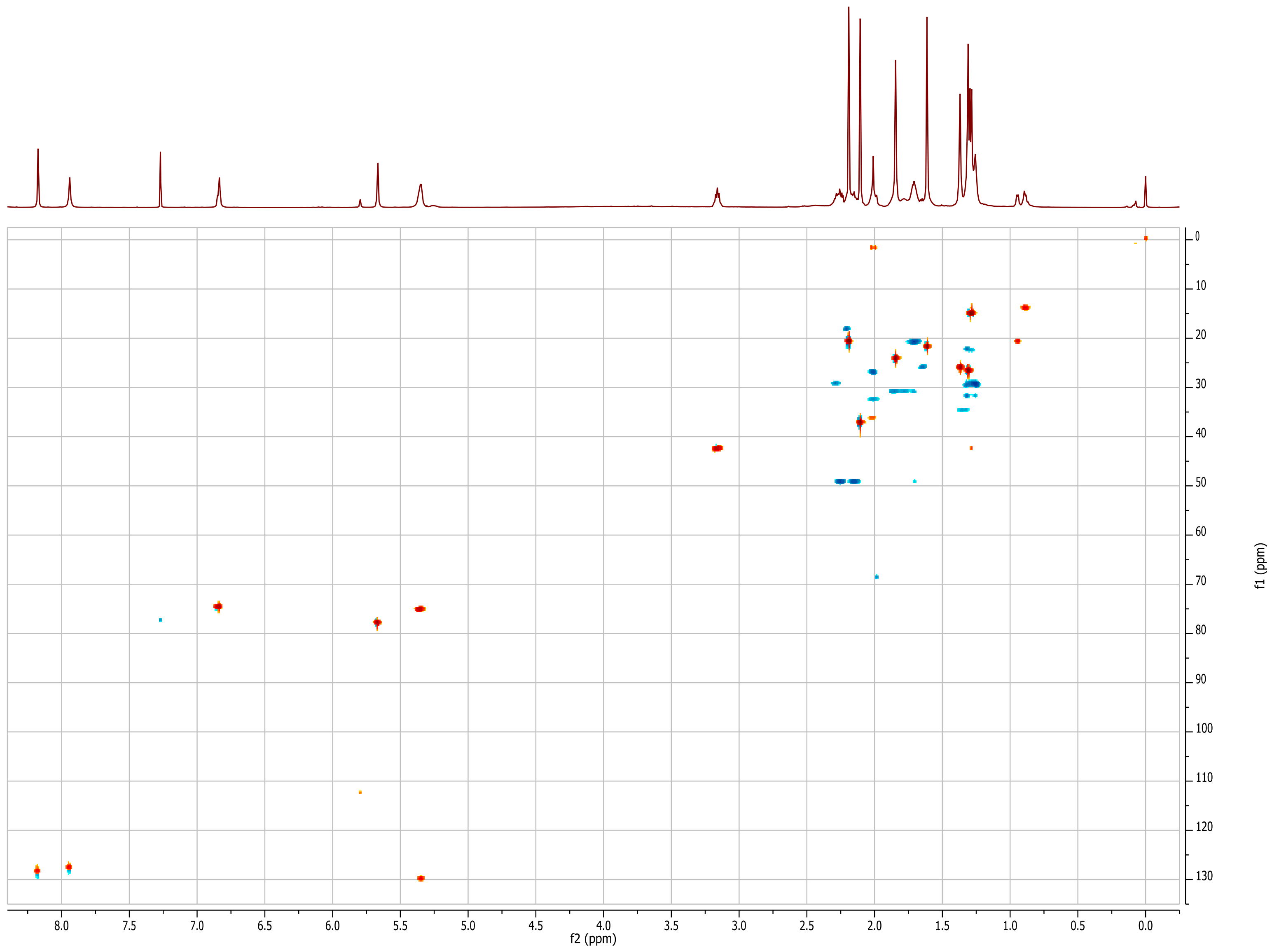

Figure N. HMBC-NMR (600 MHz, $CDCl_3$) Spectrum of Hectochlorin (1).

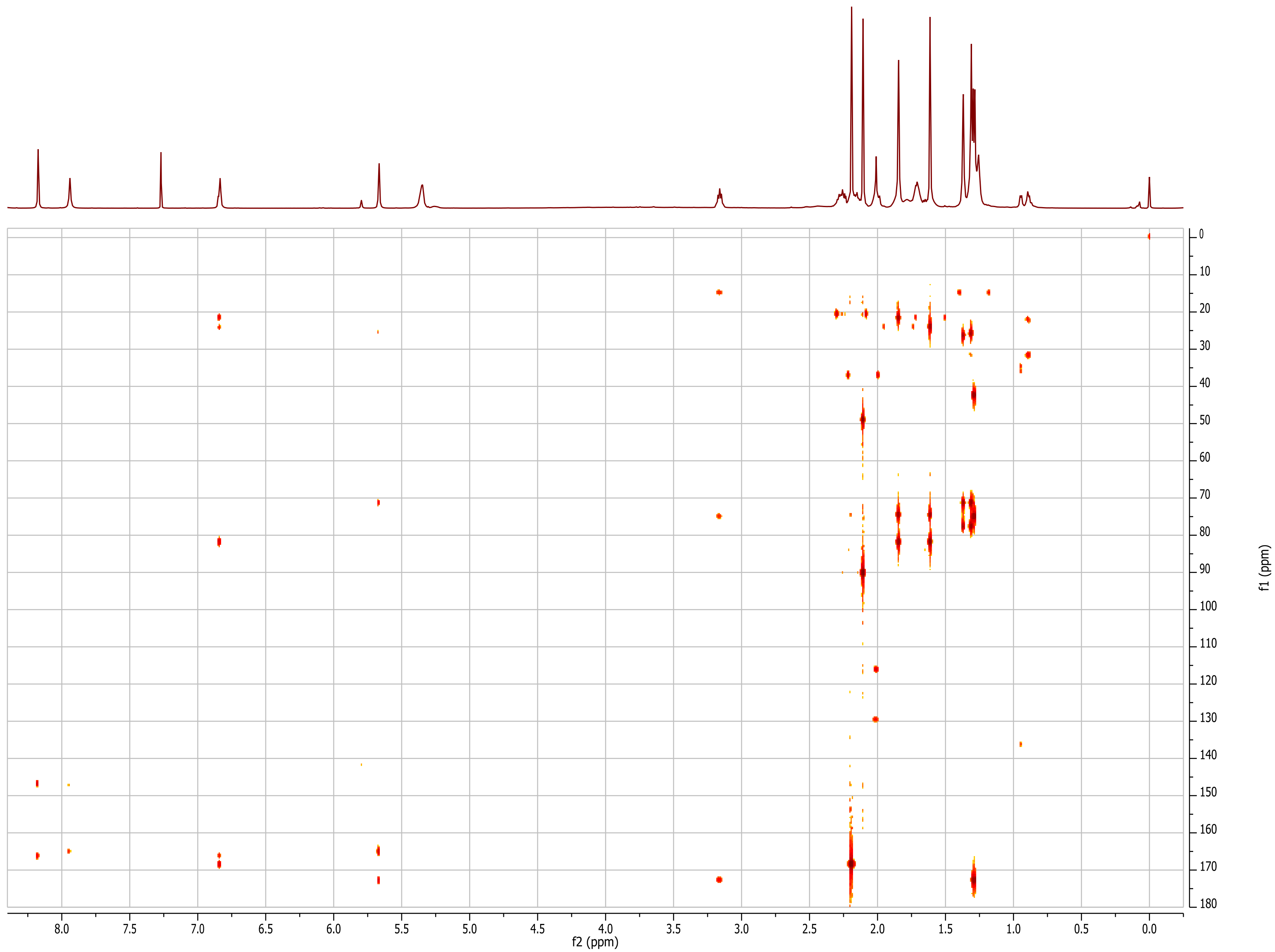

Figure O. TOCSY-NMR (600 MHz, $CDCl_3$) Spectrum of Hectochlorin (1).

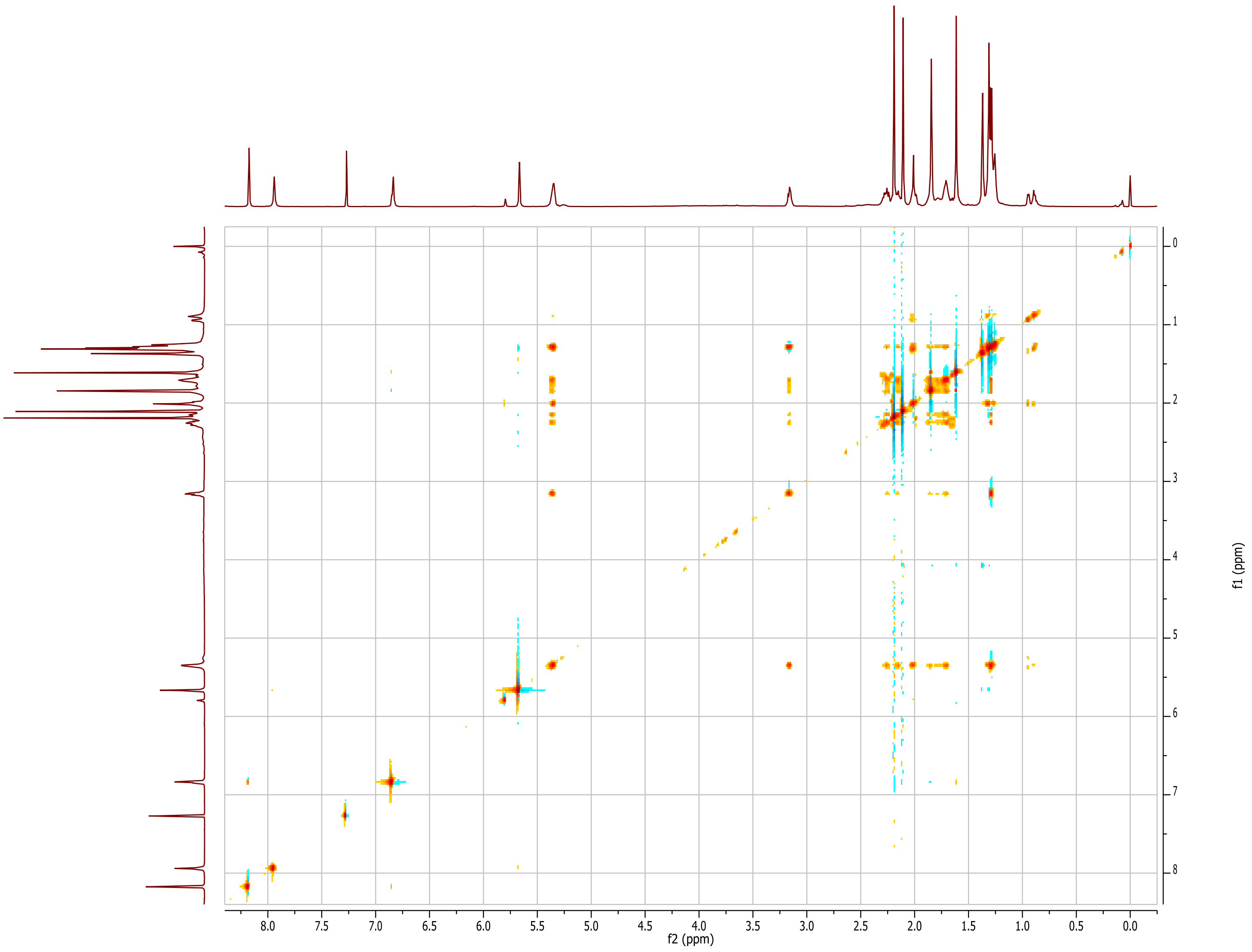

Figure P. FTMS^2 Fragment Spectrum of Hectochlorin (1).

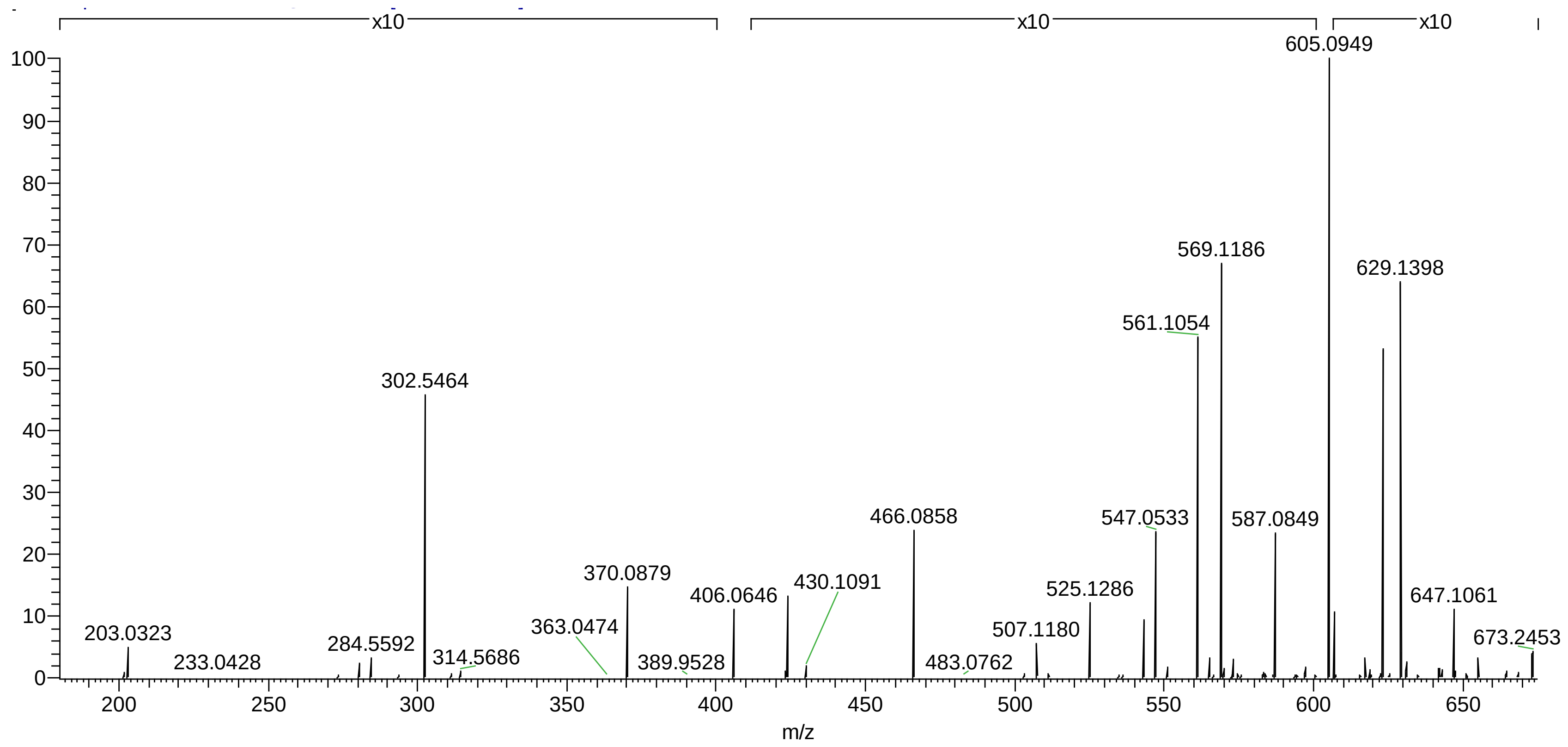

Hectochlorin (**1**) $[M + H]^+$, Parent Mass 665 *m/z*

Figure Q. FT-MS[2] Fragment Summary for Hectochlorin (1).

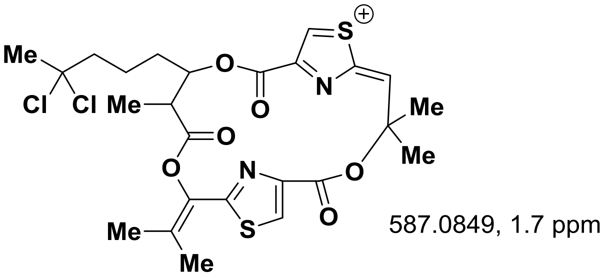

587.0849, 1.7 ppm

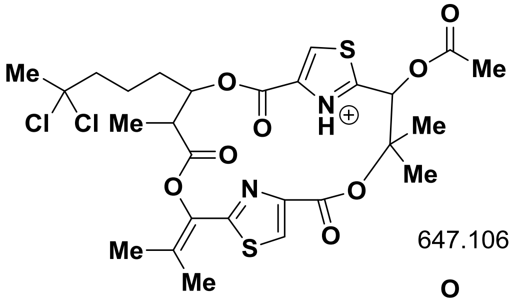

647.1061, 1.7 ppm

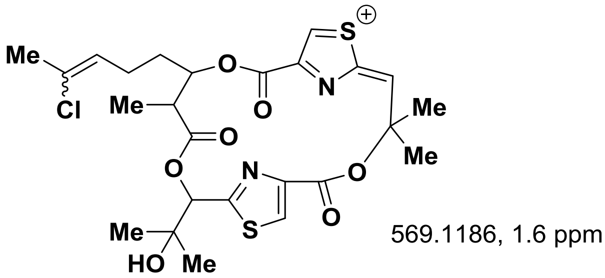

569.1186, 1.6 ppm

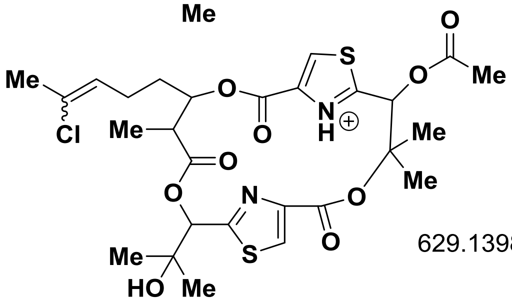

629.1398, 1.4 ppm

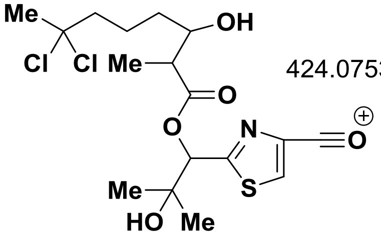

424.0753, 1.4 ppm

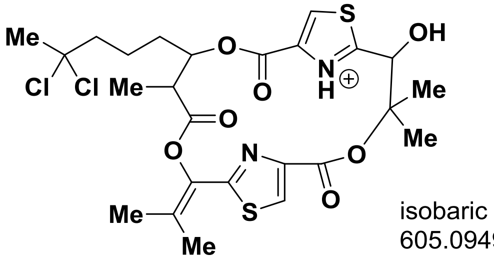

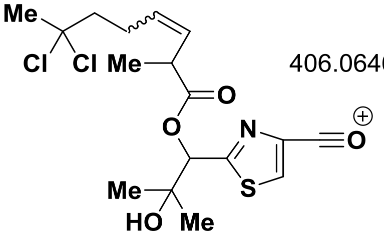

406.0646, 1.2 ppm

isobaric at:
605.0949, 0.8 ppm

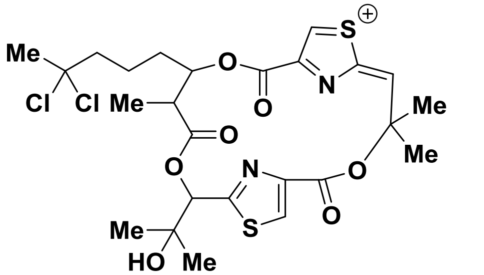

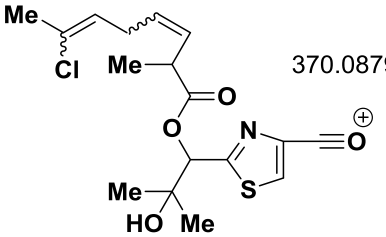

370.0879, 1.4 ppm

Table R. NMR Data Summary for Hectochlorin B (5).

Position	δ_C, type[a]	δ_H (*J* in Hz)[b]	HMBC[b]	TOCSY[b]
1	173.2, C	--		
2	42.4, CH	3.14, quintet (7.6)	1, 3, *4 weak*, 9	3, 9
3	74.2, CH_2	5.32, m	*2,4 weak*	2, 4-6, 9
4a	30.8, CH_2	1.74, m		3
4b	30.8, CH_2	1.83, m		3
5	20.6, CH_2	1.73, m		3, 6
6a	49.2, CH_2	2.12, m	5, 7, *4, 8 weak*	3, 5, 6b
6b	49.2, CH_2	2.25, m	5, 7, *4, 8 weak*	3, 5, 6a
7	90.3, C	--		
8	37.2, CH_3	2.09, s	6, 7, self	
9	14.9, CH_3	1.28, d (7.2)[c]	1-3, self	2, 3
10	160.6, C			
11	146.3, C			
12	128.3, CH	8.11, s	10, 11, 13	
13	171.4, C			
14	76.3, CH	5.09, bs		
15	85.5, C			
16	26.7, CH_3	1.84, s	14, 15, 17	
17	22.2, CH_3	1.77, s	14-16	
18	162.3, C			
19	147.0, C			
20	128.5, CH	8.04, s	18, 19, 21	
21	164.9, C			
22	77.2, CH	5.58, s	1, 21, 23, 24	
23	71.6, C			
24	25.7, CH_3	1.35, s	22, 23, 25	
25	26.9, CH_3	1.28, s[c]	22-24	

All experiments in $CDCl_3$ with TMS standard, 1% v/v for 13C experiment, and 0.3% v/v for the rest. [a]Carbon chemical shifts from 2D-NMR experiment extraction. [b]Spectra collected on a Bruker 600 MHz (600 MHz and 150 MHz for the ^1H and ^{13}C nuclei, respectively) with 1.7 mm inverse cryo-probe. [c]Proton species overlapped, shift and coupling extracted from the HSQC.

Figure S. ^{1}H-NMR (600 MHz, $CDCl_3$) Spectrum of Hectochlorin B (5).

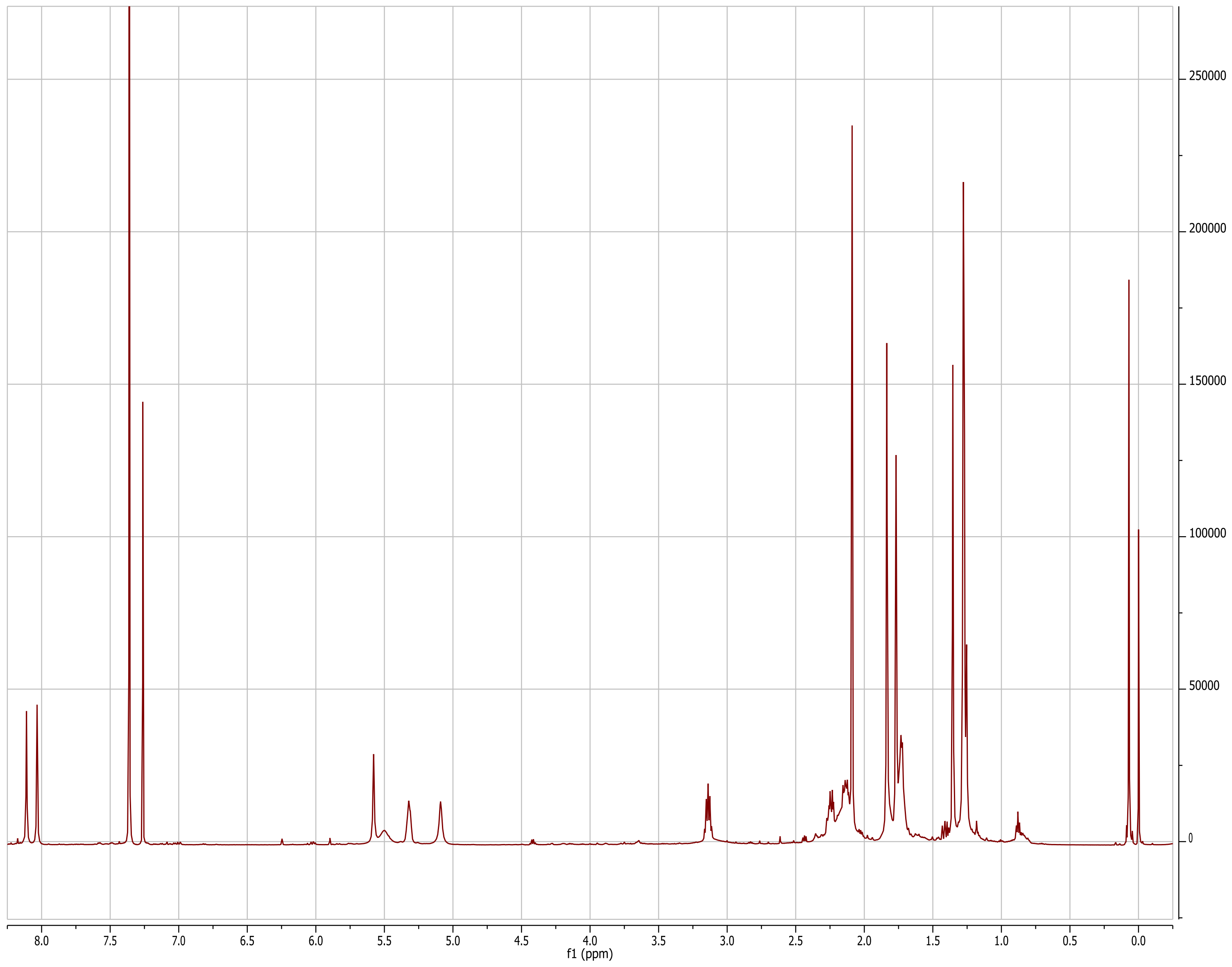

Figure T. ^{13}C-NMR (125 MHz, $CDCl_3$) Spectrum of Hectochlorin B (5).

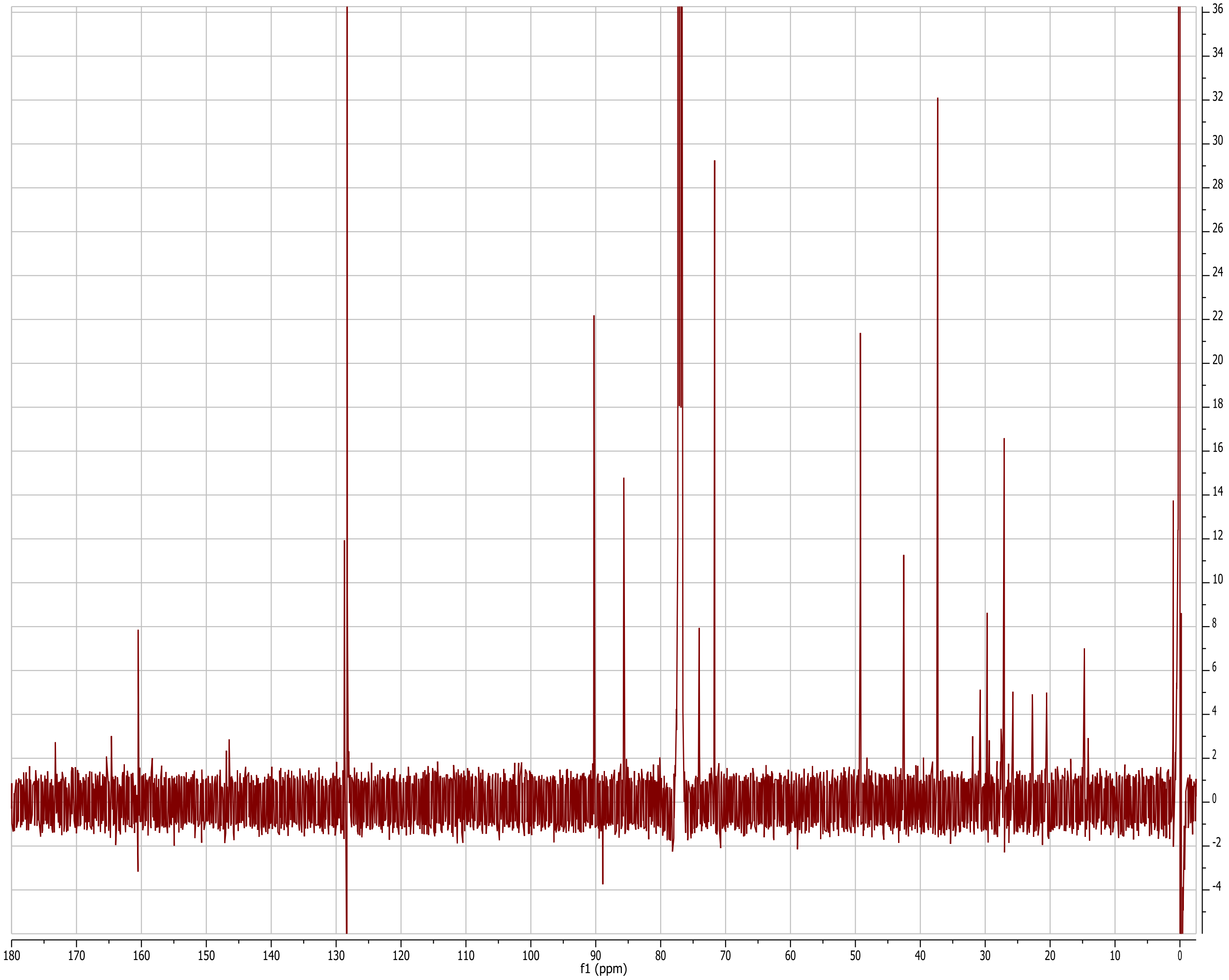

Benzene contamination at 128.3 ppm.

Figure U. **HSQC-NMR (600 MHz, $CDCl_3$) Spectrum of Hectochlorin B (5).**

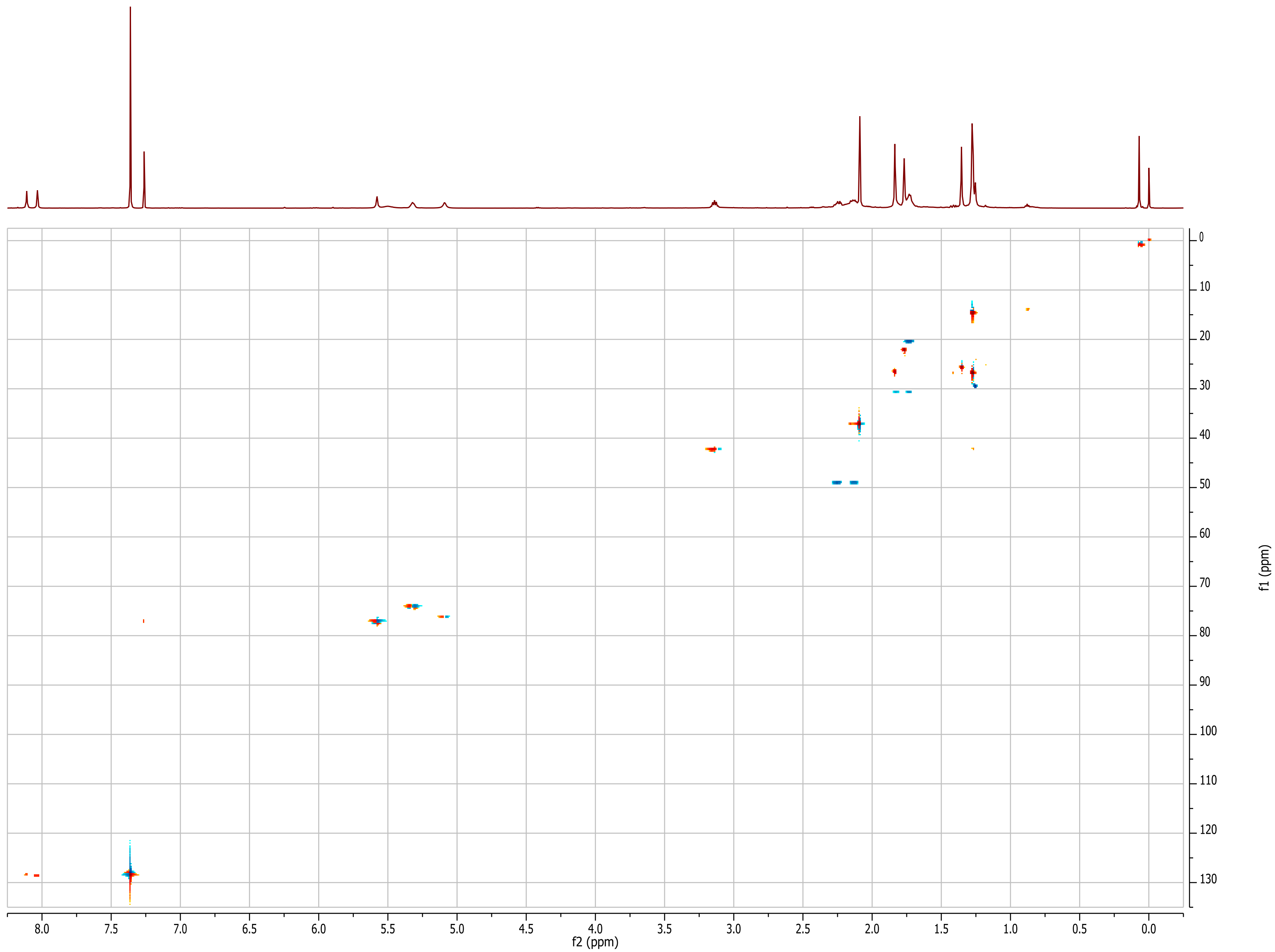

Figure V. HMBC-NMR (600 MHz, $CDCl_3$) Spectrum of Hectochlorin B (5).

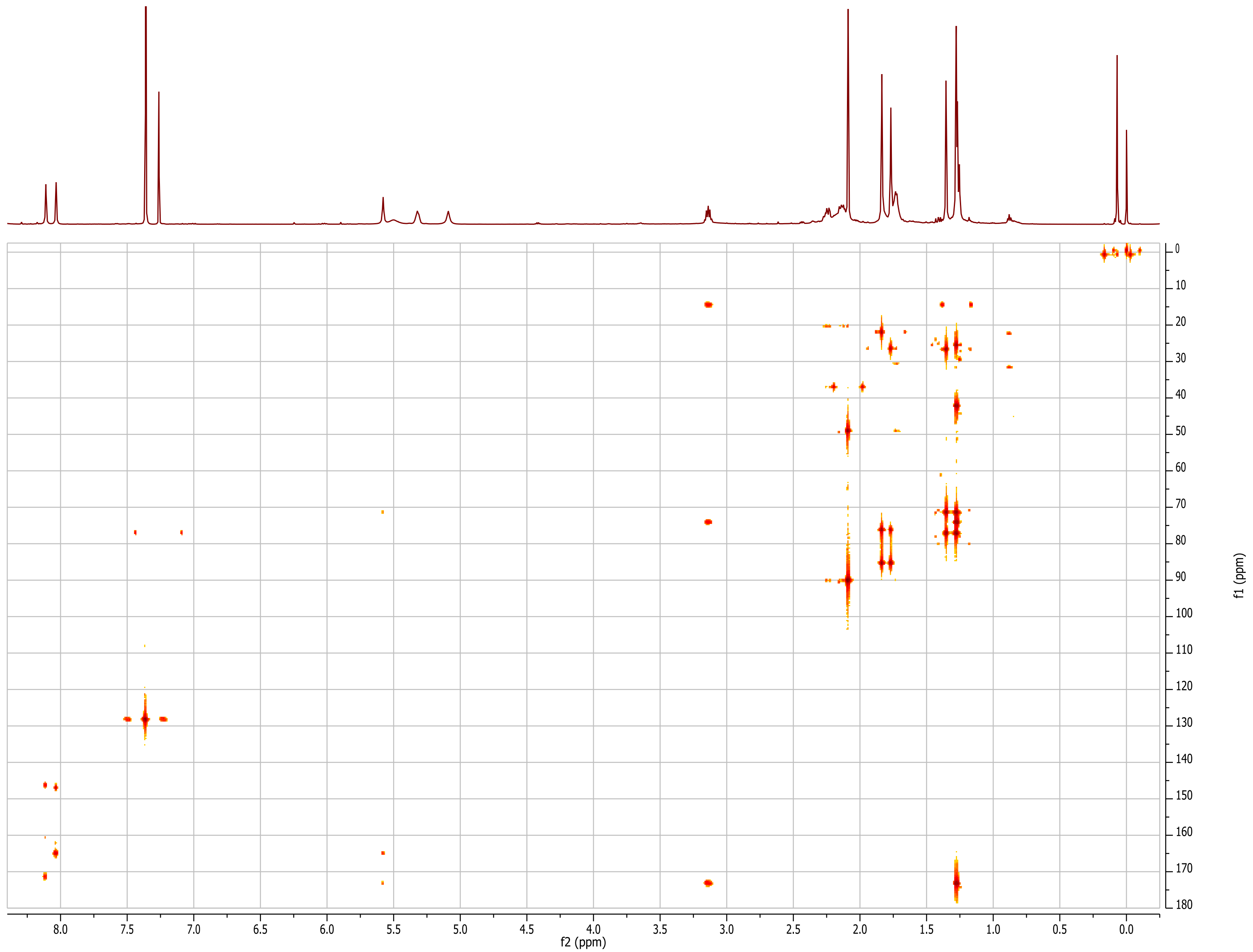

Figure W. TOCSY-NMR (600 MHz, $CDCl_3$) Spectrum of Hectochlorin B (5).

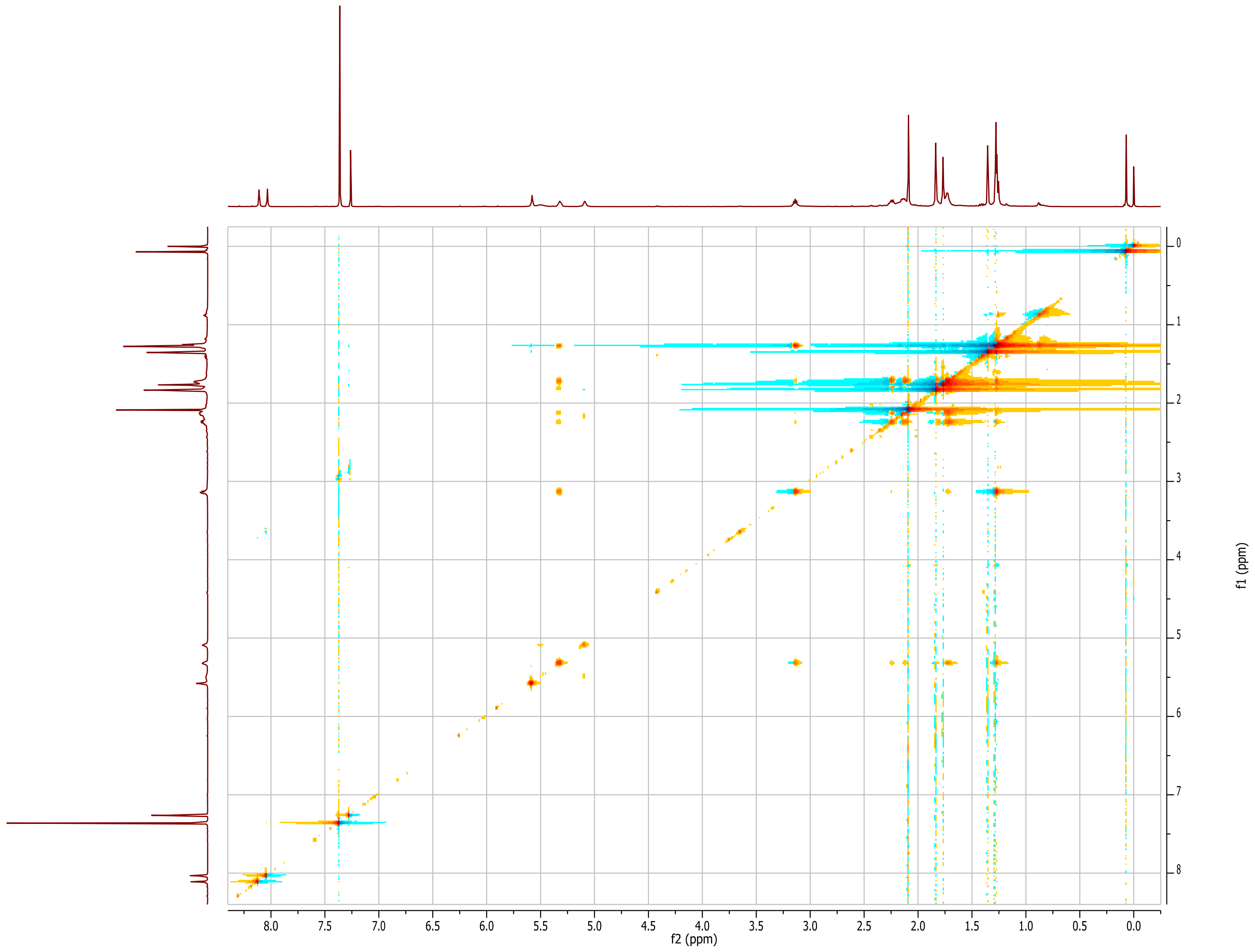

Figure X. FTMS^2 Fragment Spectrum of Hectochlorin B (5).

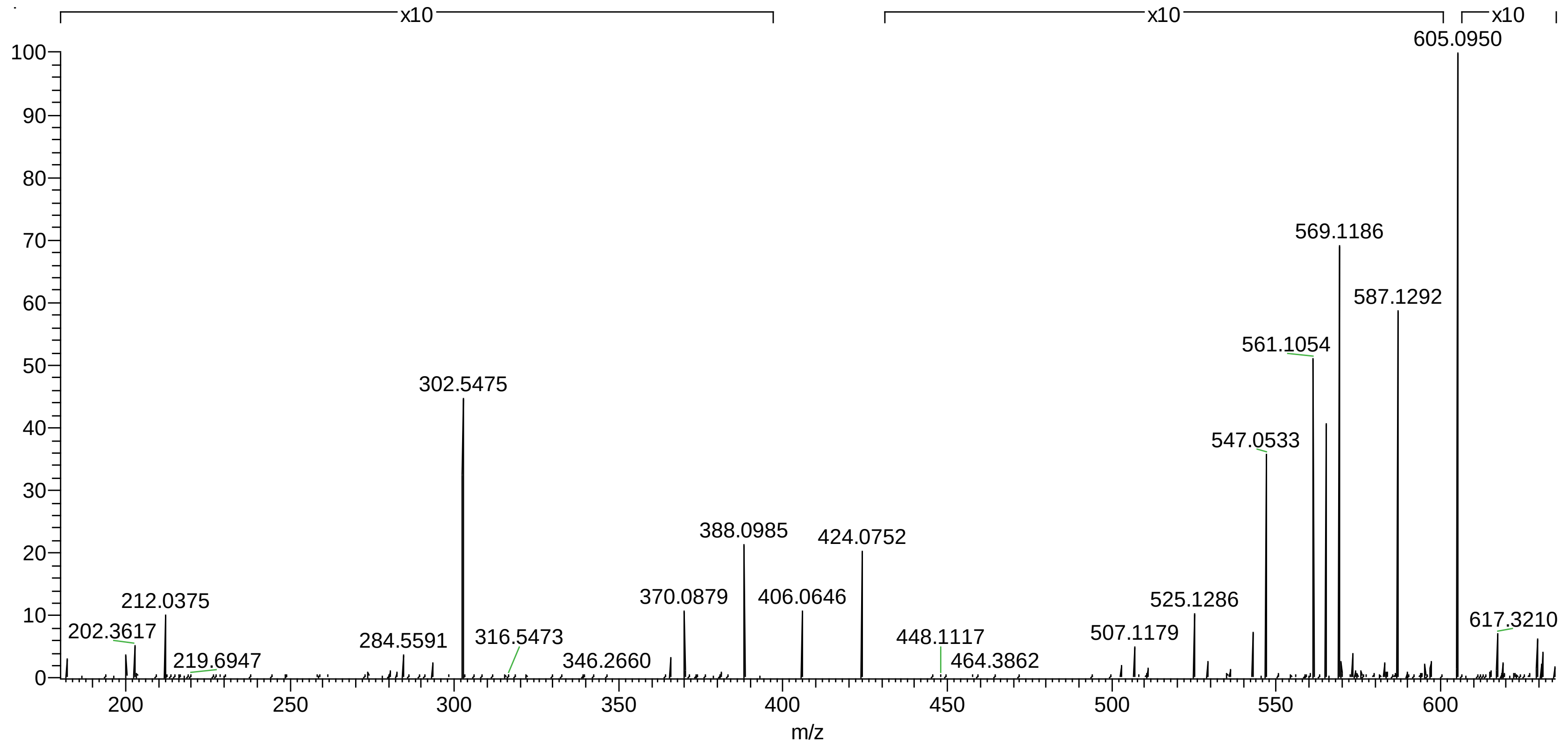

Hectochlorin B (**5**) $[M + H]^+$, Parent Mass 623 *m/z*

Figure Y. FT-MS^2 Fragment Summary for Hectochlorin B (5).

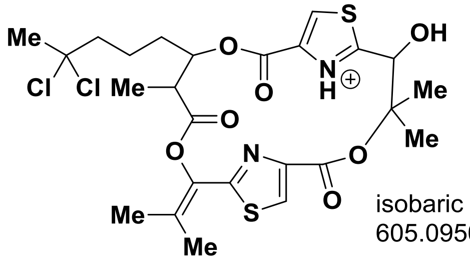

isobaric at:
605.0950, 1.0 ppm

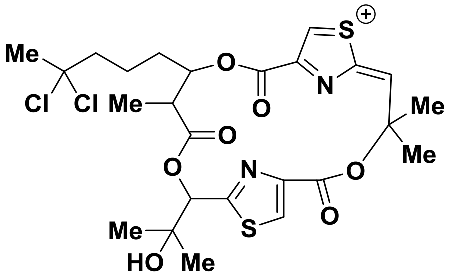

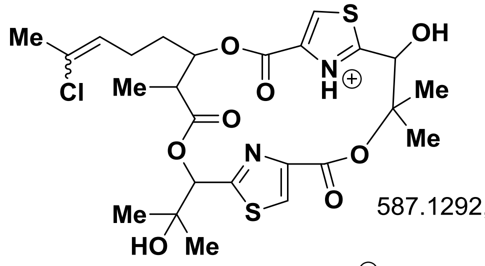

587.1292, 1.5 ppm

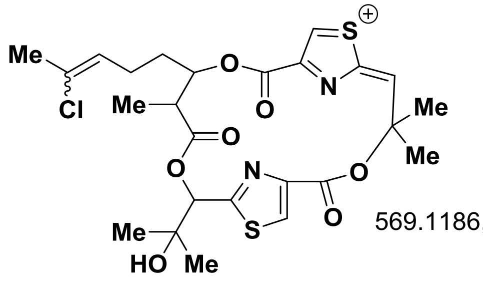

569.1186, 1.6 ppm

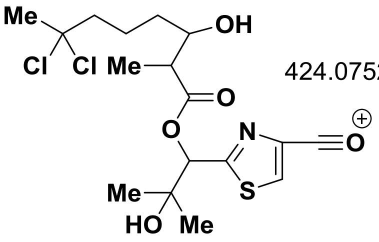

424.0752, 1.2 ppm

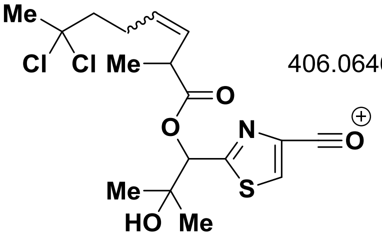

406.0646, 1.2 ppm

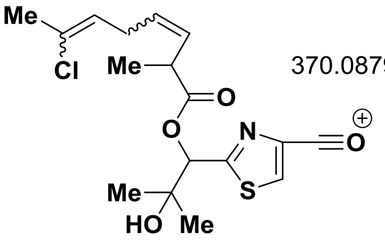

370.0879, 1.4 ppm

Figure Z. FTMS^2 Fragment Spectrum for Hectochlorin C (6).

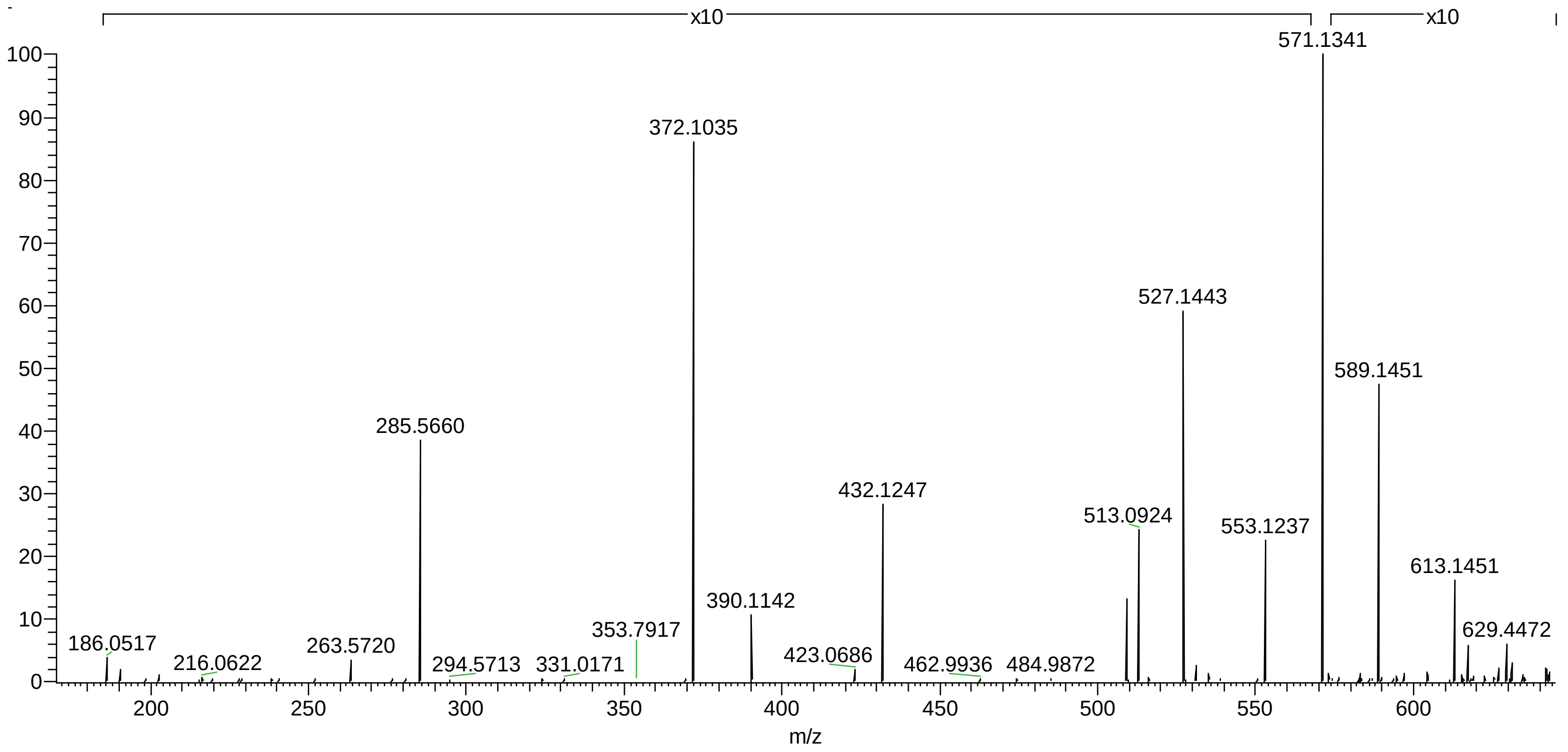

Hectochlorin C (**6**) $[M + H]^+$, Parent Mass 631 *m/z*

Figure AA. FTMS^2 Fragment Summary for Hectochlorin C (6).

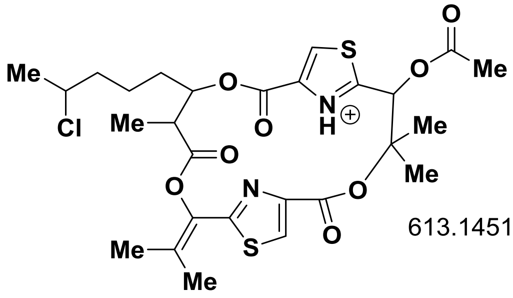

613.1451, 1.8 ppm

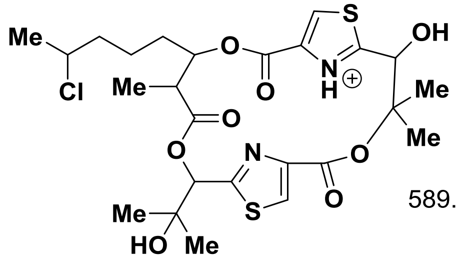

589.1451, 1.9 ppm

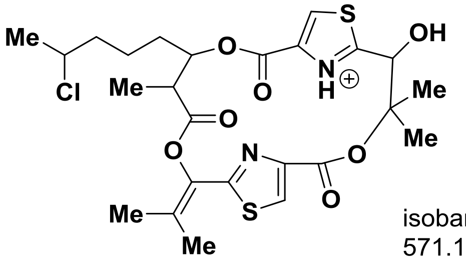

isobaric at:
571.1341, 1.2 ppm

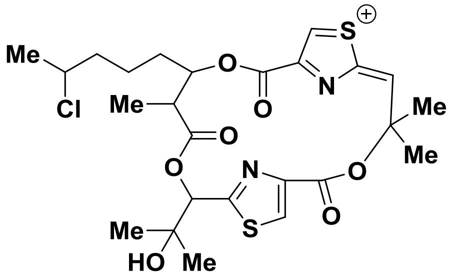

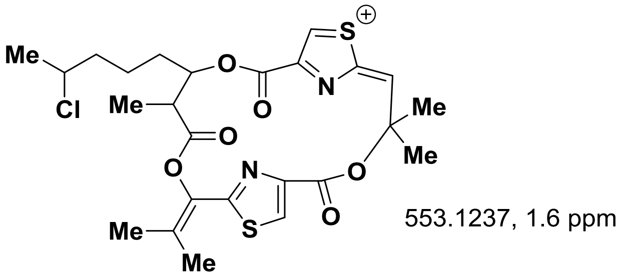

553.1237, 1.6 ppm

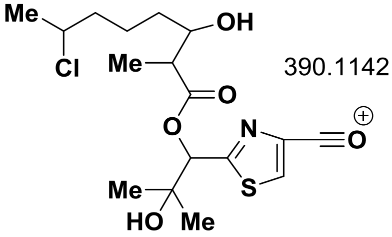

390.1142, 1.5 ppm

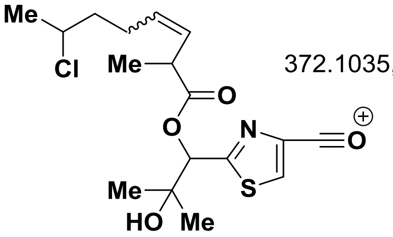

372.1035, 1.2 ppm

Figure AB. FTMS^2 Fragment Spectrum for Hectochlorin D (7).

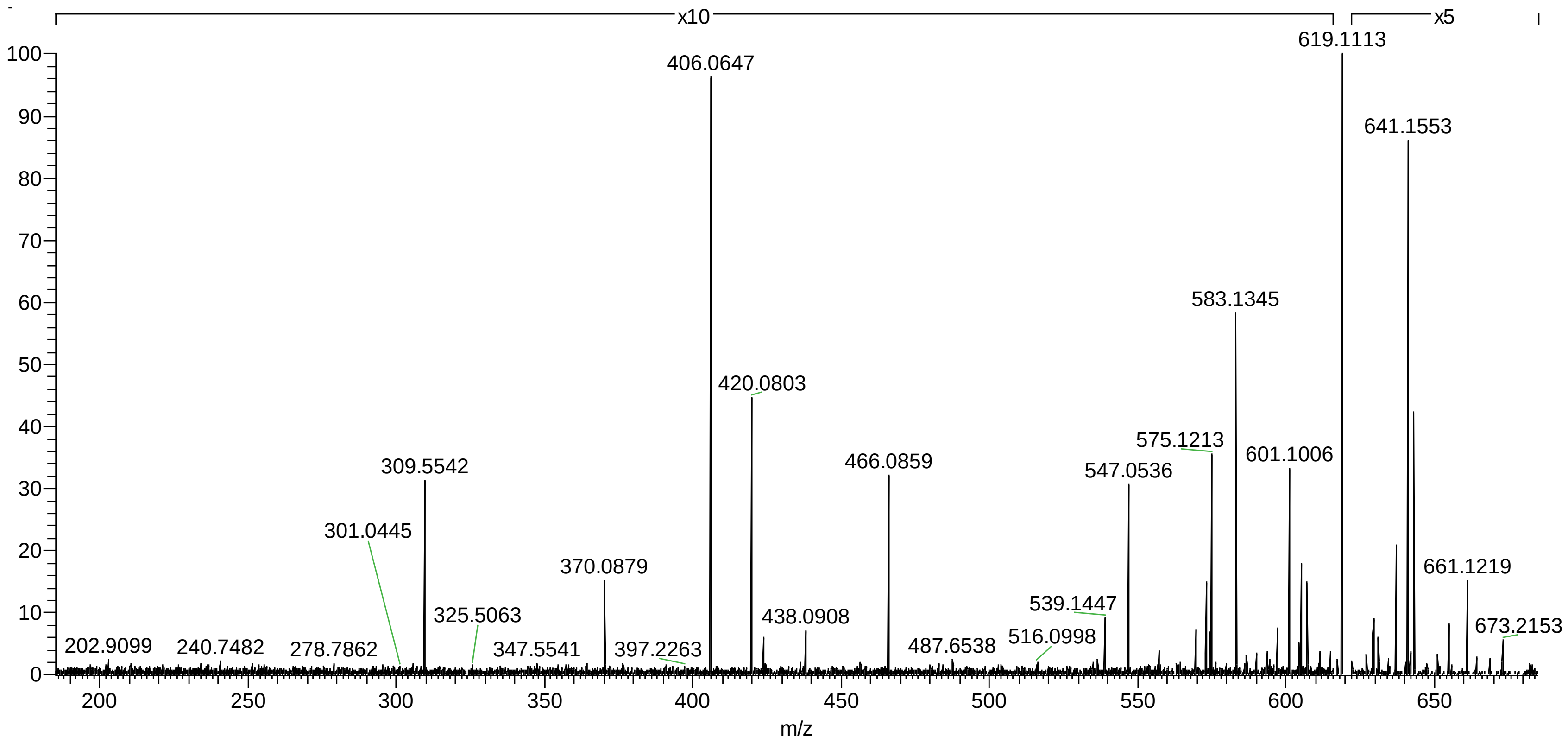

Hectochlorin D (**7**) $[M + H]^+$, Parent Mass 679 *m/z*

Figuve AC. FTMS^2 Fragment Summary for Hectochlorin D (7).

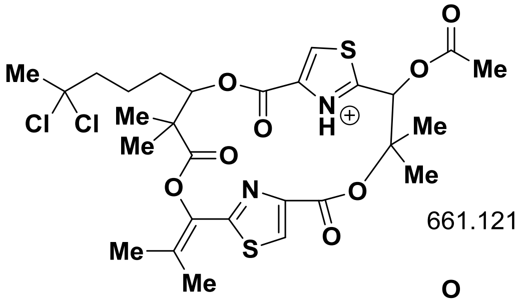

661.1219, 2.0 ppm

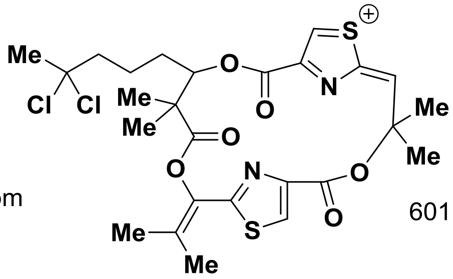

601.1006, 1.8 ppm

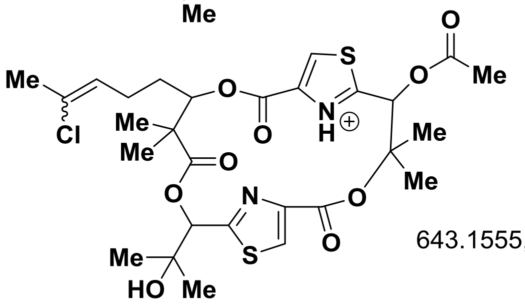

643.1555, 1.6 ppm

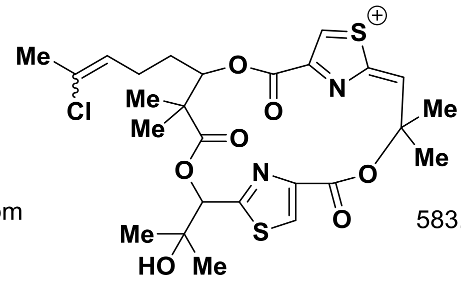

583.1345, 1.9 ppm

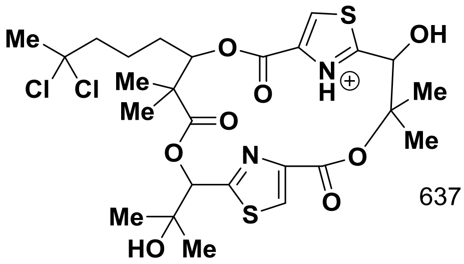

637.1219, 2.0 ppm

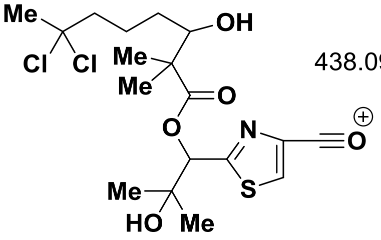

438.0908, 1.1 ppm

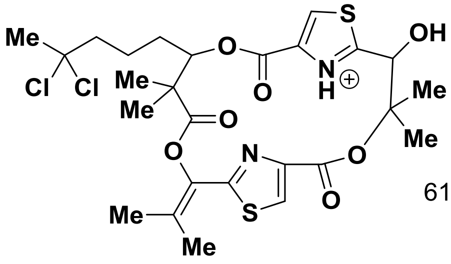

619.1101, 1.9 ppm

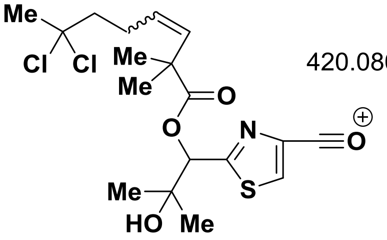

420.0803, 1.2 ppm

Figure AD. Proposed Biosynthetic Pathway Divergence for Jamaicamide A and D (2 and 8).

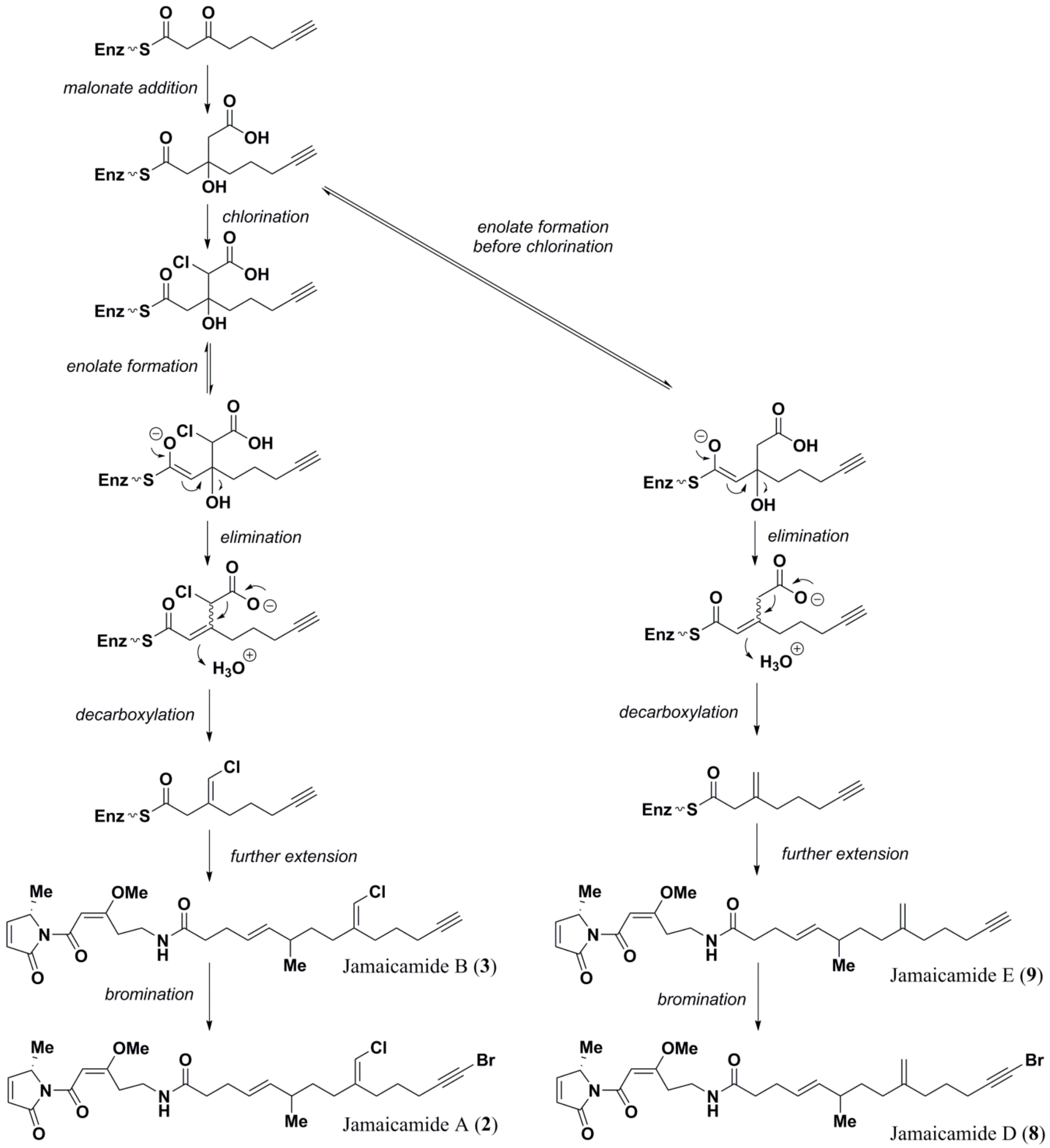

Table AE. NMR Data Summary for Jamaicamide D (8).

Position	δ_C[a]	δ_H (*J* in Hz)	HMBC	TOCSY
1		1.50[b]		3
2		--		
3		2.20, t (7.1)		1, 4, 5
4		1.60-1.66, m		3, 5
5		2.06-2.10, m		3, 4
6		--		
7		2.01-2.05, m		8, 26
8	34.9	1.29-1.40, m		7, 9, 26
9	36.6	1.91-1.95, m		8, 26
10	137.1	5.26-5.39, m[c]		12, 13, 26
11		5.26-5.39, m[c]		12, 13, 26
12		2.26-2.30, m		10, 11, 13a
13a		2.17-2.20, m		10, 11, 12, 13b
13b		2.26-2.30, m		10, 11, 13a
14		--		
NH		6.69, bs		15
15		3.47-3.54, m		16, NH
16a		2.81-2.86, m		15, 16b
16b		2.98-3.51, m		15, 16a
17	175.6	--		
18		6.73, s		
19		--		
20		--		
21		6.08, dd (1.4, 6.0)		22
22	153.1	7.23, dd (2.0, 5.9)		21, 24
23	58.2	4.86, q (6.7)		24
24		1.46, d (6.7)	22, 23	22, 23
25		3.75, s	17	
26		0.95, d (6.7)	8-10	7-11
27		4.70, m		

All experiments in $CDCl_3$ with 0.3% v/v TMS standard, on a Varian Unity 500 MHz (500 MHz and 125 MHz for the ^1H and ^{13}C nuclei respectively). [a]Carbon chemical shifts by projection from HMBC experiment. [b]Overlapping with water peak, shift determined by TOCY correlation. [c]Proton species overlapped.

Figure AF. [1]**H-NMR (500 MHz, $CDCl_3$) Spectrum of Jamaicamide D (8).**

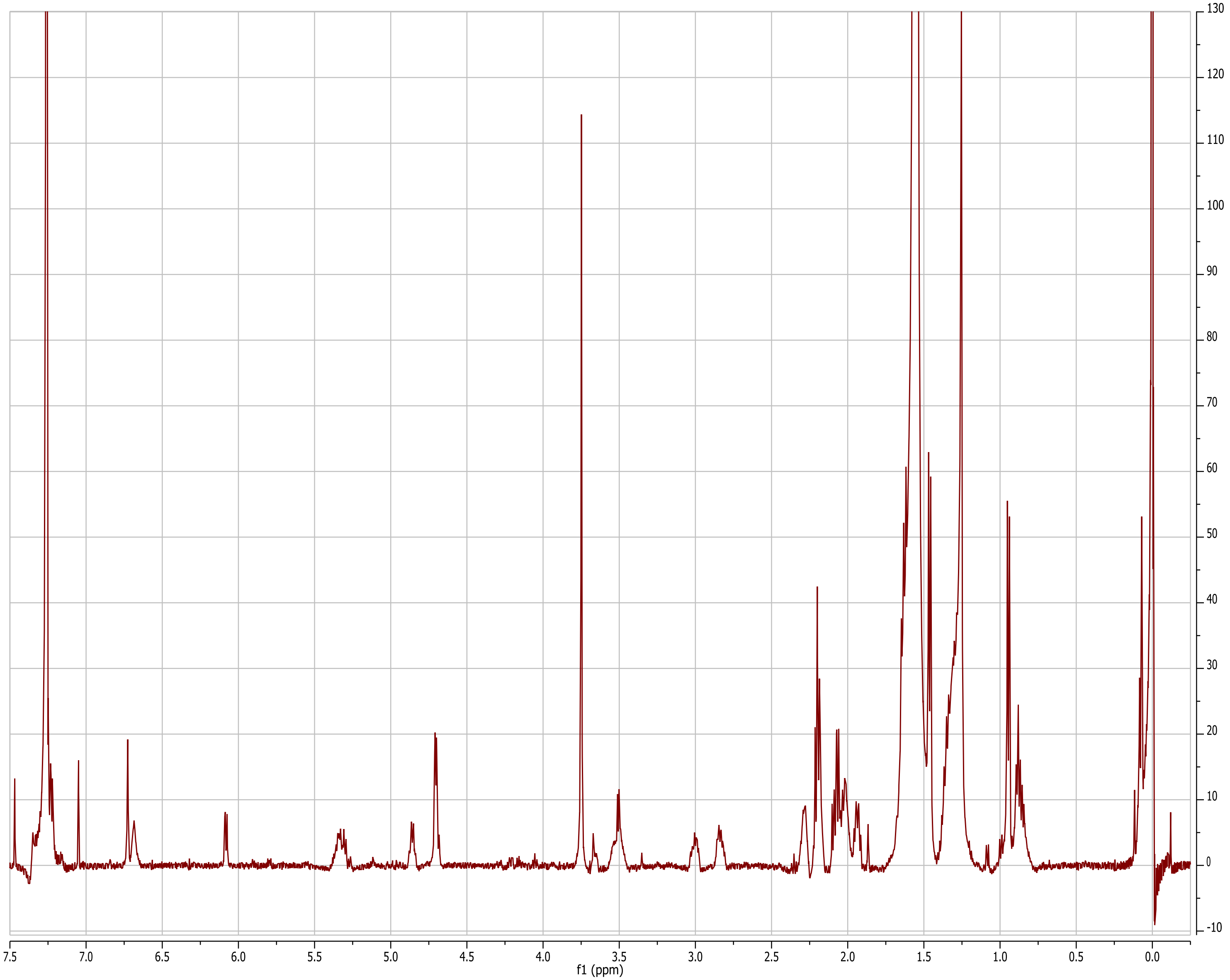

Figure AG. HMBC-NMR (500 MHz, $CDCl_3$) Spectrum of Jamaicamide D (8).

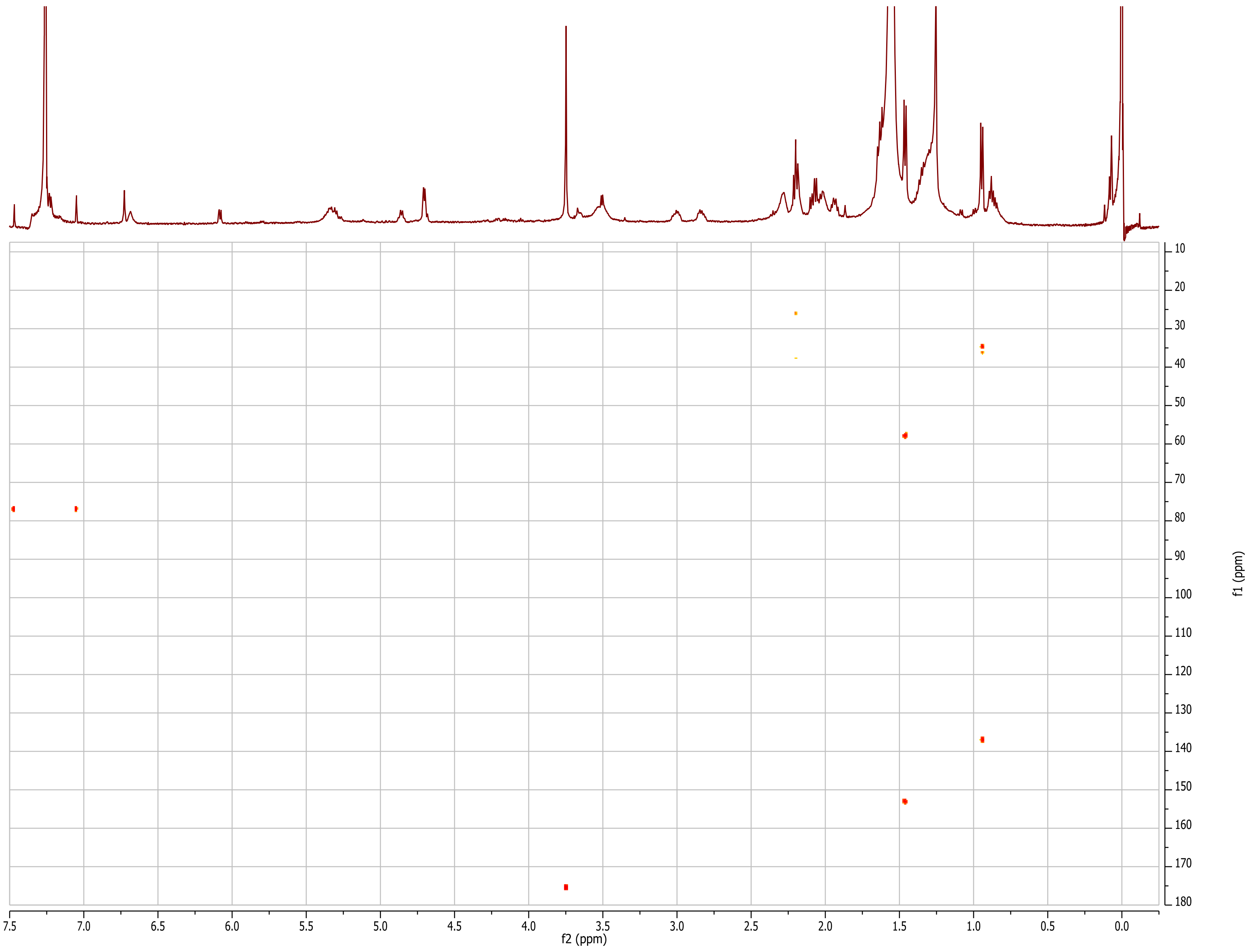

Figure AH. TOCSY-NMR (500 MHz, $CDCl_3$) Spectrum of Jamaicamide D (8).

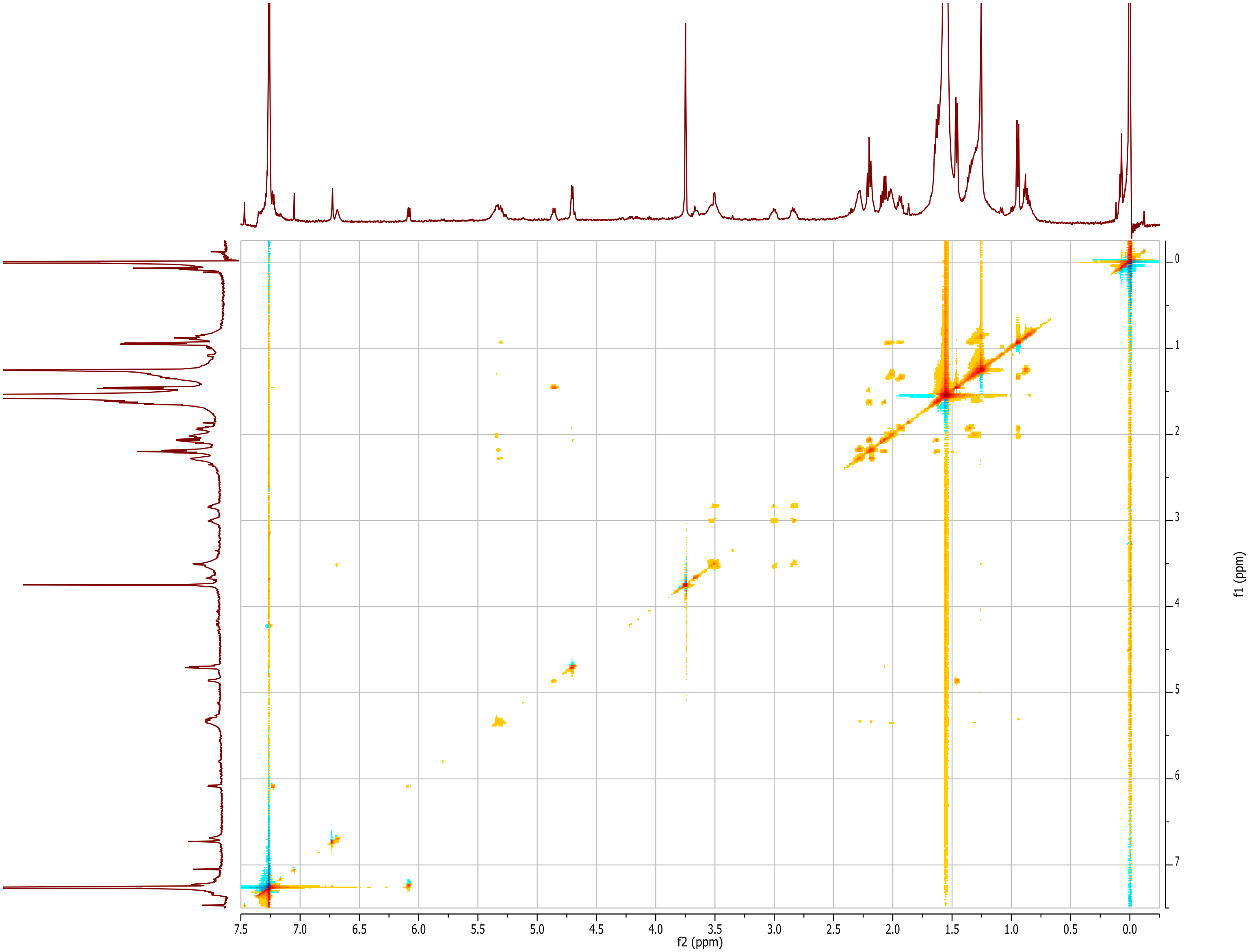

Table AI. NMR Data Summary for Jamaicamide F (12).

Position	δ_C, type[a]	δ_H (*J* in Hz)[b]	HMBC[b]	TOCSY[b]
1	-6.6, C	--		
2	94.2, C	--		
3	20.7, CH_2	2.36, t (7.2)	1, 2, 4, 5, self	4, 5
4	26.1, CH_2	1.64, quin (7.1)	2, 3, 5, 6, self	3, 5
5	29.2, CH_2	2.25,[ac] m	3, 4, 6, 7, 27	3, 4
6	141.8, C	--		
7	32.6, CH_2	2.00,[bd] m	5, 6, 8, 9, 27	8, 26
8	34.7, CH_2	1.34,[ce] m	6, 10, 9, 7, 26	7, 9, 26
9	36.3, CH	2.02,[bd] m	7, 10, 11, 26	8, 26
10	136.6, CH	5.27, dd (7.7, 15.4)	8, 9, 11, 12, 26, self	9, 11, 12, 13a, 26
11	127.5, CH	5.36, td (6.5, 15.2)	9, 10, 12	9, 10, 12, 13a, 26
12	28.6, CH_2	2.29,[ac] m		10, 11, 13a
13a	36.8, CH_2	2.18, t (7.6)	11, 12, 14	10, 11, 12, 13b
13b	36.8, CH_2	2.29,[ac] m	10, 11, 14, self	13a
14	172.4, C	--		
NH	--	6.69, bs	14, 15	15, 16
15a	38.3, CH_2	3.29,[ce] m	17, self	NH, 15b, 16
15b	38.3, CH_2	3.54,[ce] m	17, self	NH, 15a, 16
16a	32.3, CH_2	2.84,[ce] m	15, 17, 18	NH, 15, 16b
16b	32.3, CH_2	3.00,[ce] m	15, 17, 18	NH, 15, 16a
17	175.4, C	--		
18	95.0, CH	6.73, s	16, 17, self	
19	166.1, C	--		
20	170.1, C	--		
21	125.9, CH	6.08, dd (6.0, 1.6)	20, 22, 23, 24, self	22-24
22	153.2, CH	7.23, dd (2.0, 6.1)	20, 21, 23, 24, self	21, 23, 24
23	58.1, CH	4.86, tq (1.4, 7.0)	*19, 20 weak*, 21, 22, 24	21, 22, 24
24	17.9, CH_3	1.46, d (6.7)	22, 23, self	21-23
25	56.2, CH_3	3.75, s	17, self	
26	20.9, CH_3	0.95, d (6.7)	8-10, self	8-11
27	112.8, CH	5.79, s	5-7, self	

All experiments in $CDCl_3$ with TMS standard, 1% v/v for 13C experiment, and 0.3% v/v for the rest. [a]Specturm collected on a Varian VX 500 MHz with ^{13}C-optimized cryoprobe. [b]Spectra collected on a Bruker 600 MHz (600 MHz and 150 MHz for the ^1H and ^{13}C nuclei, respectively) with 1.7 mm inverse cryo-probe. [c,d]Proton species overlapped, shift assigned by HSQC. [e]Multiplet shift assigned by HSQC

Figure AJ. ^{1}H-NMR (600 MHz, $CDCl_3$) Spectrum of Jamaicamide F (12).

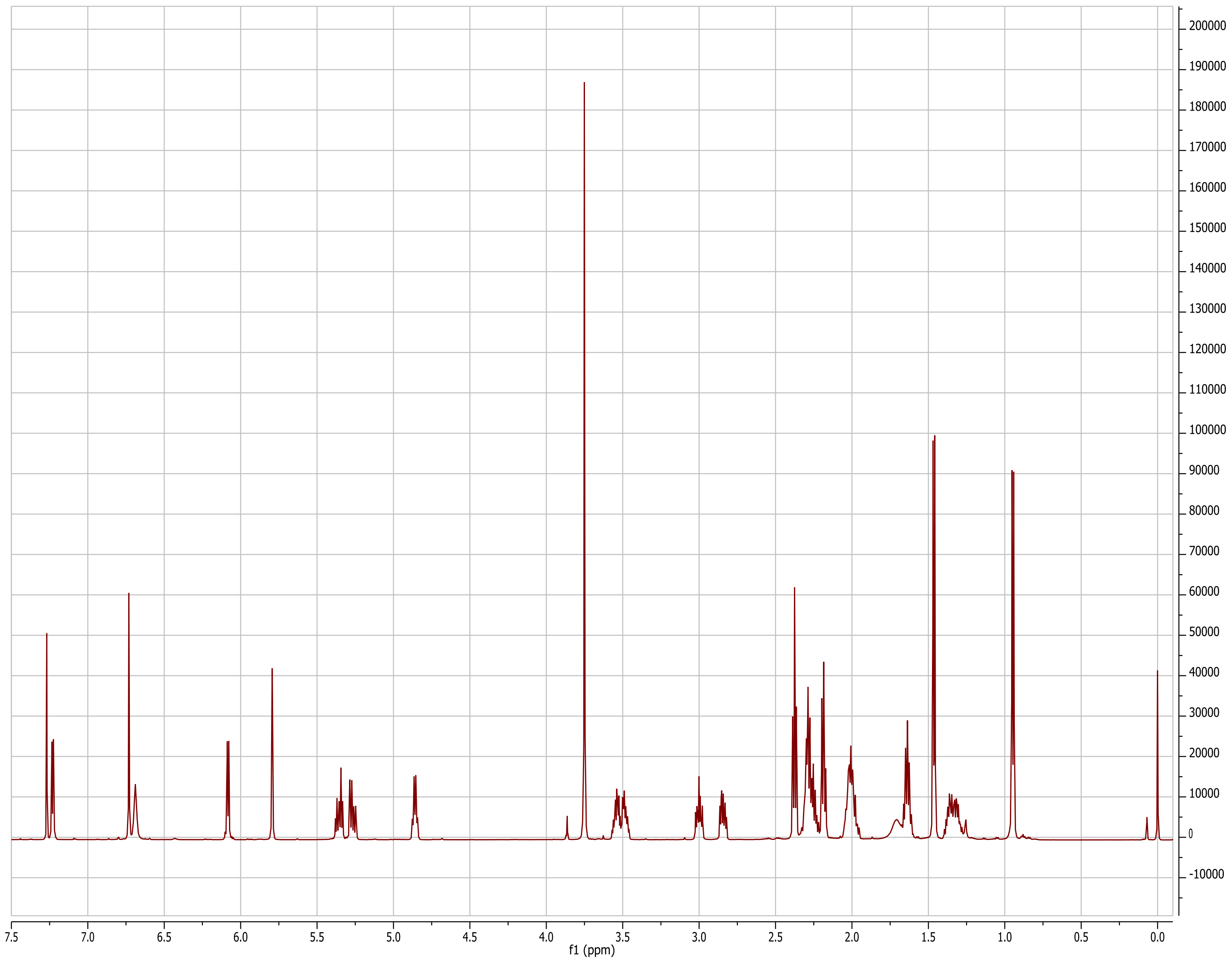

Figure AK. ^{13}C-NMR (125 MHz, $CDCl_3$) Spectrum of Jamaicamide F(12).

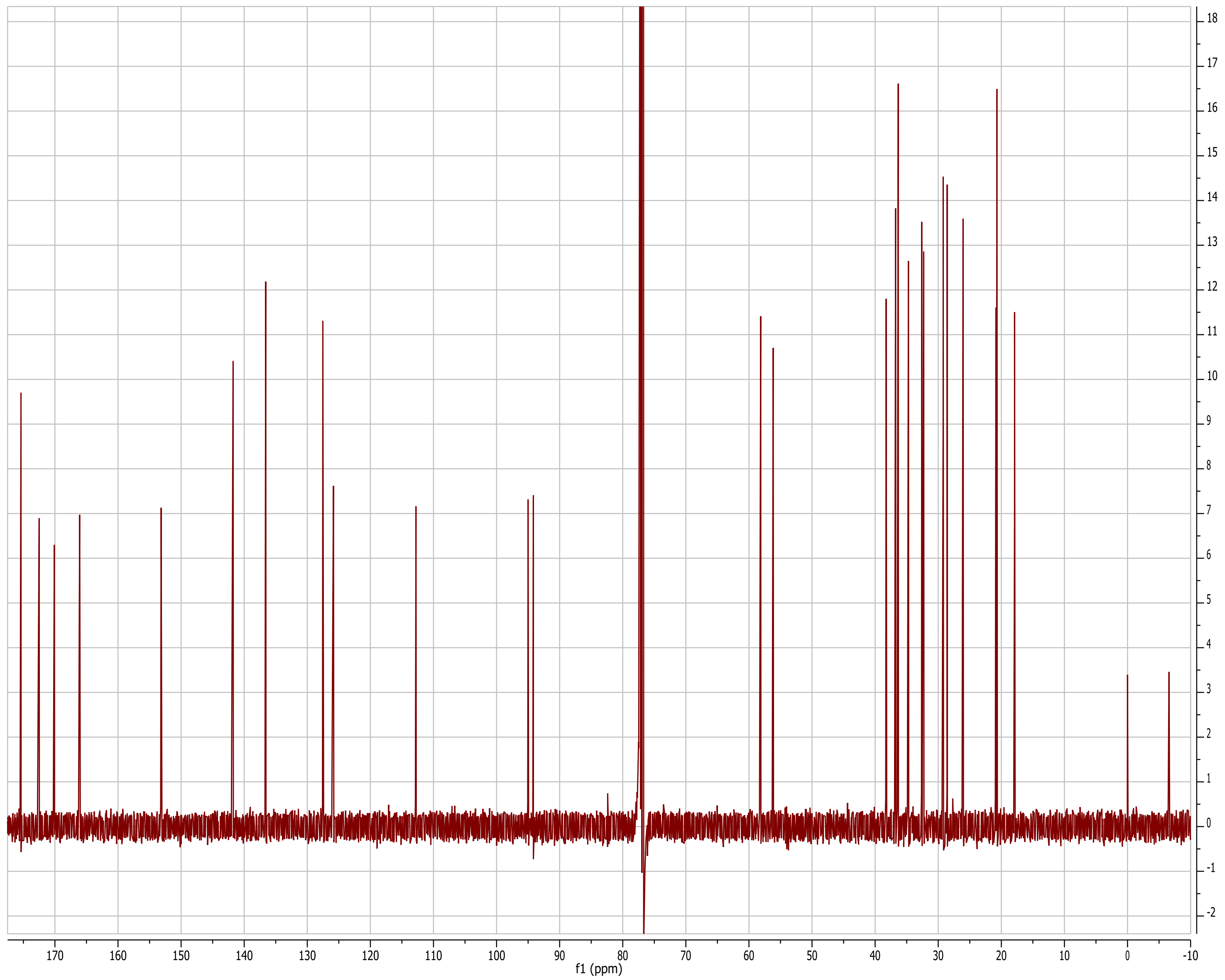

Figure AL. HSQC-NMR (600 MHz, $CDCl_3$) Spectrum of Jamaicamide F (12).

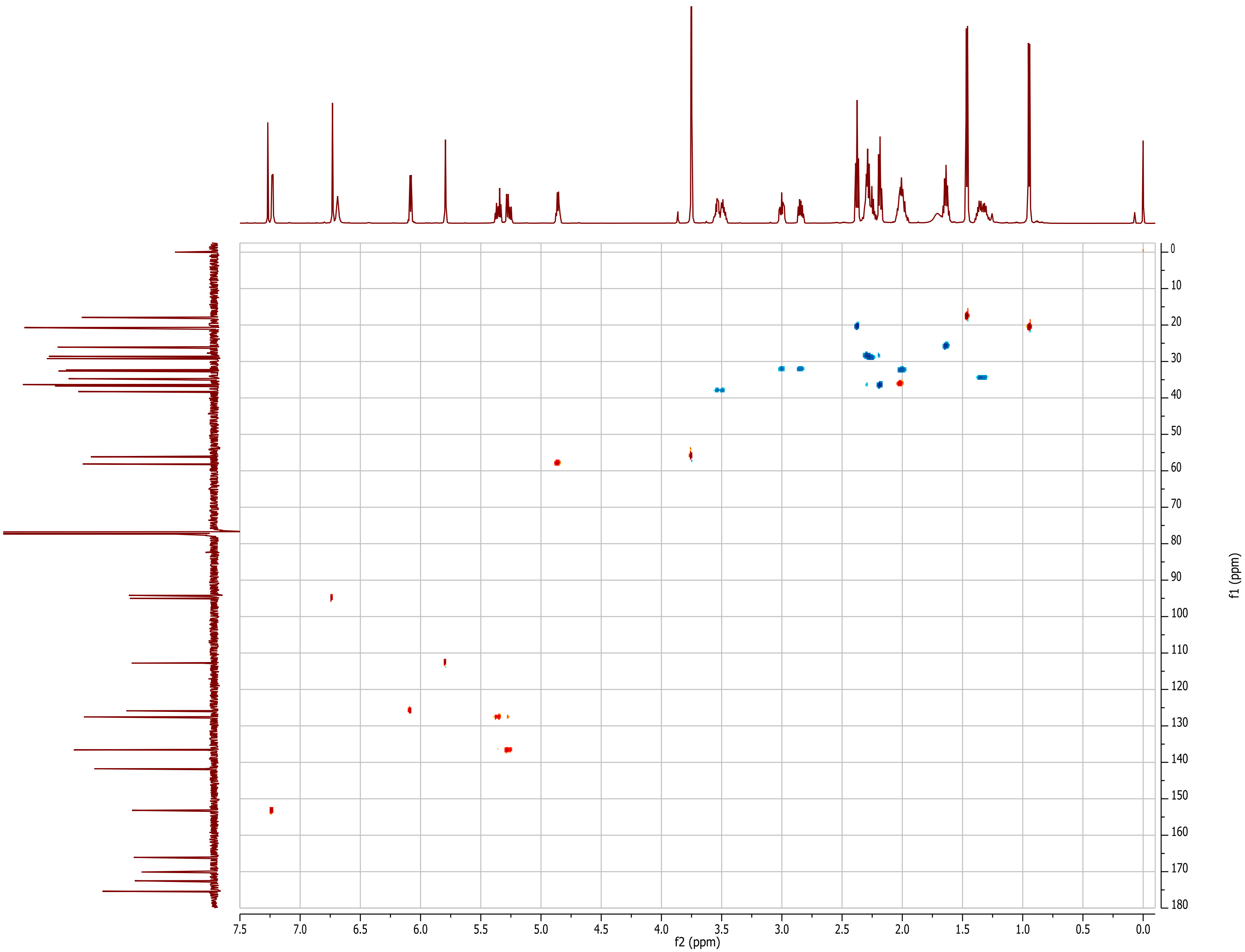

Figure AM. HMBC-NMR (600 MHz, $CDCl_3$) Spectrum of Jamaicamide F (12).

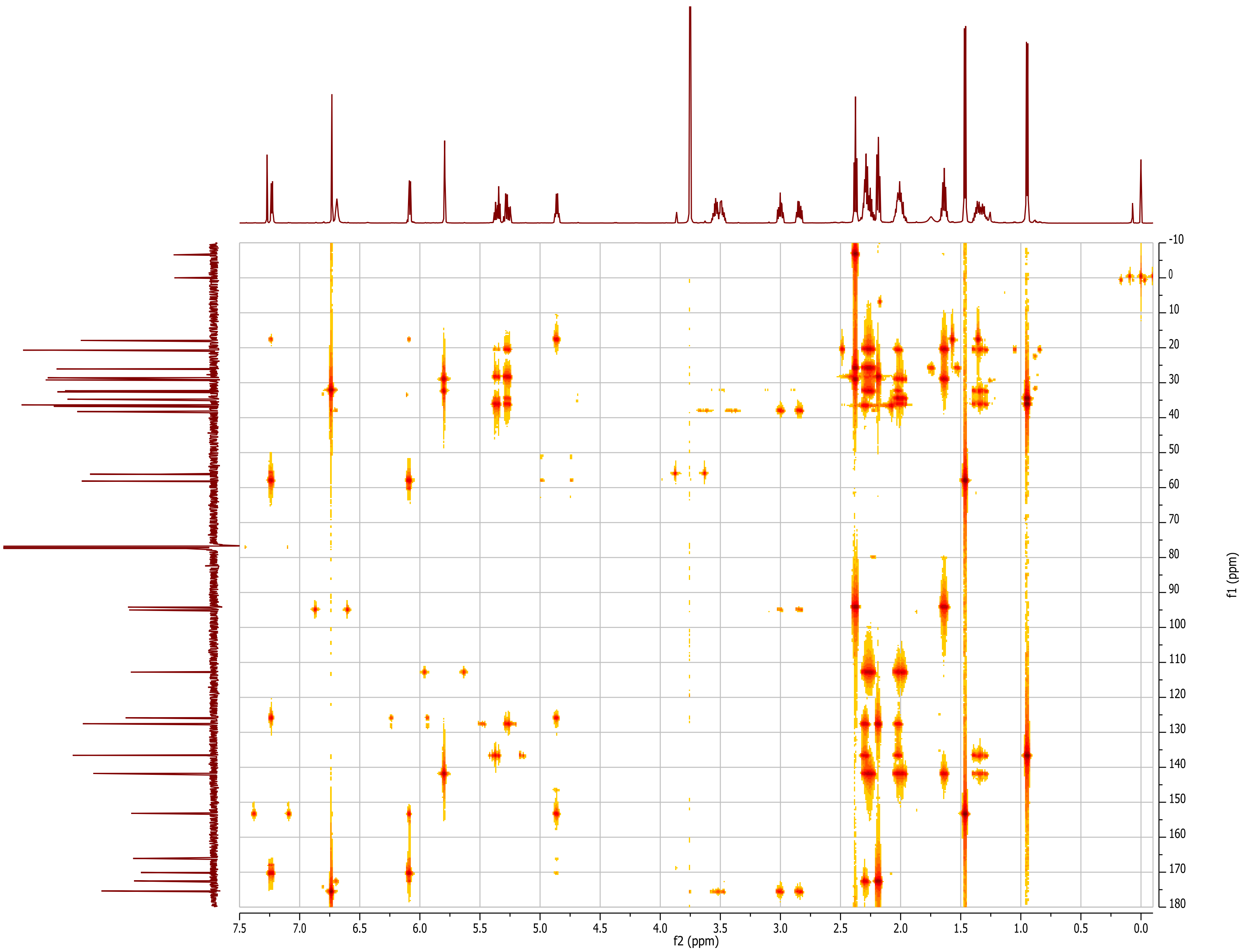

Figure AN. TOCSY-NMR (600 MHz, $CDCl_3$) Spectrum of Jamaicamide F (12).

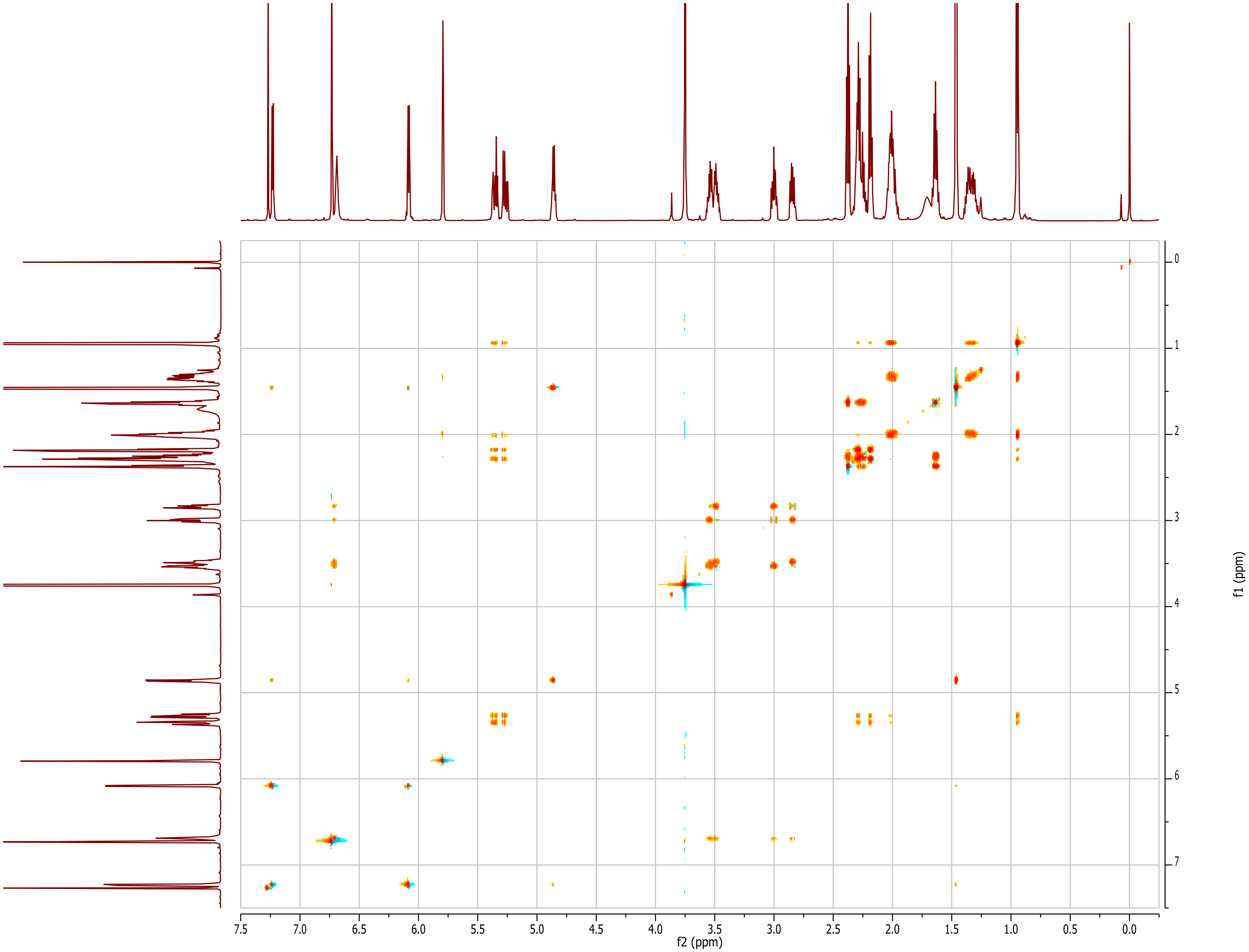

Figure AO. MS^2 Fragment Spectra of Jamaicamide F (12).

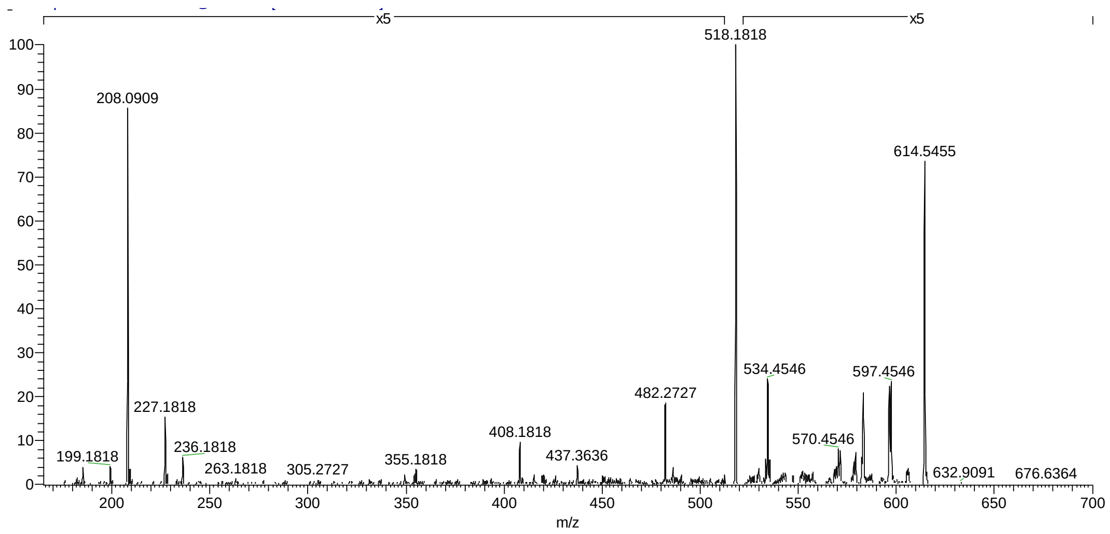

$ITMS^2$ spectrum, parent mass of 615, $[M+H]^+$ of jamaicamide F (**12**).

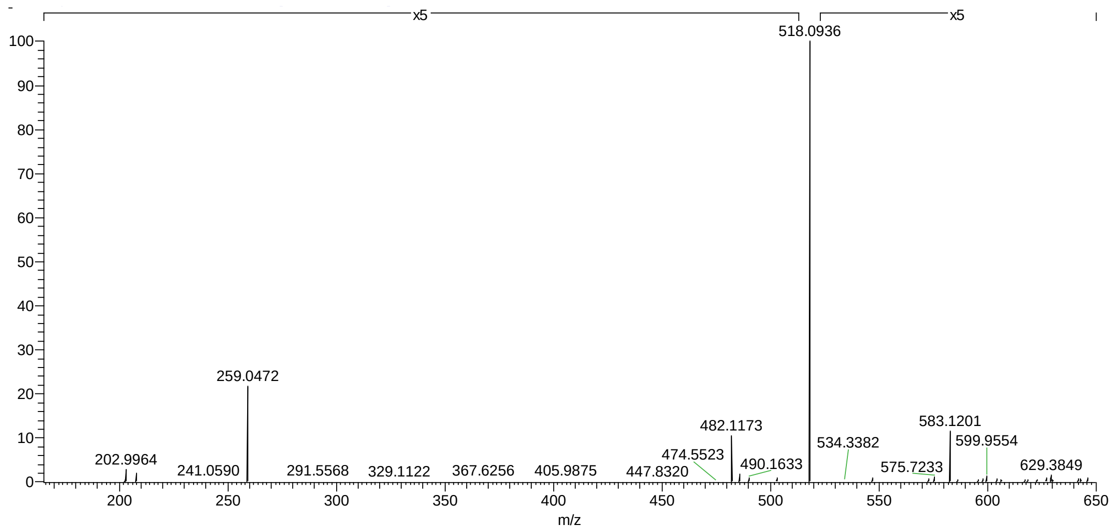

$FTMS^2$ spectrum, parent mass of 615, $[M+H]^+$ of jamaicamide F (**12**).

Figure AP. Effect of the jamaicamides on the veratridine-induced Ca^{2+} influx in murine neocortical neurons.

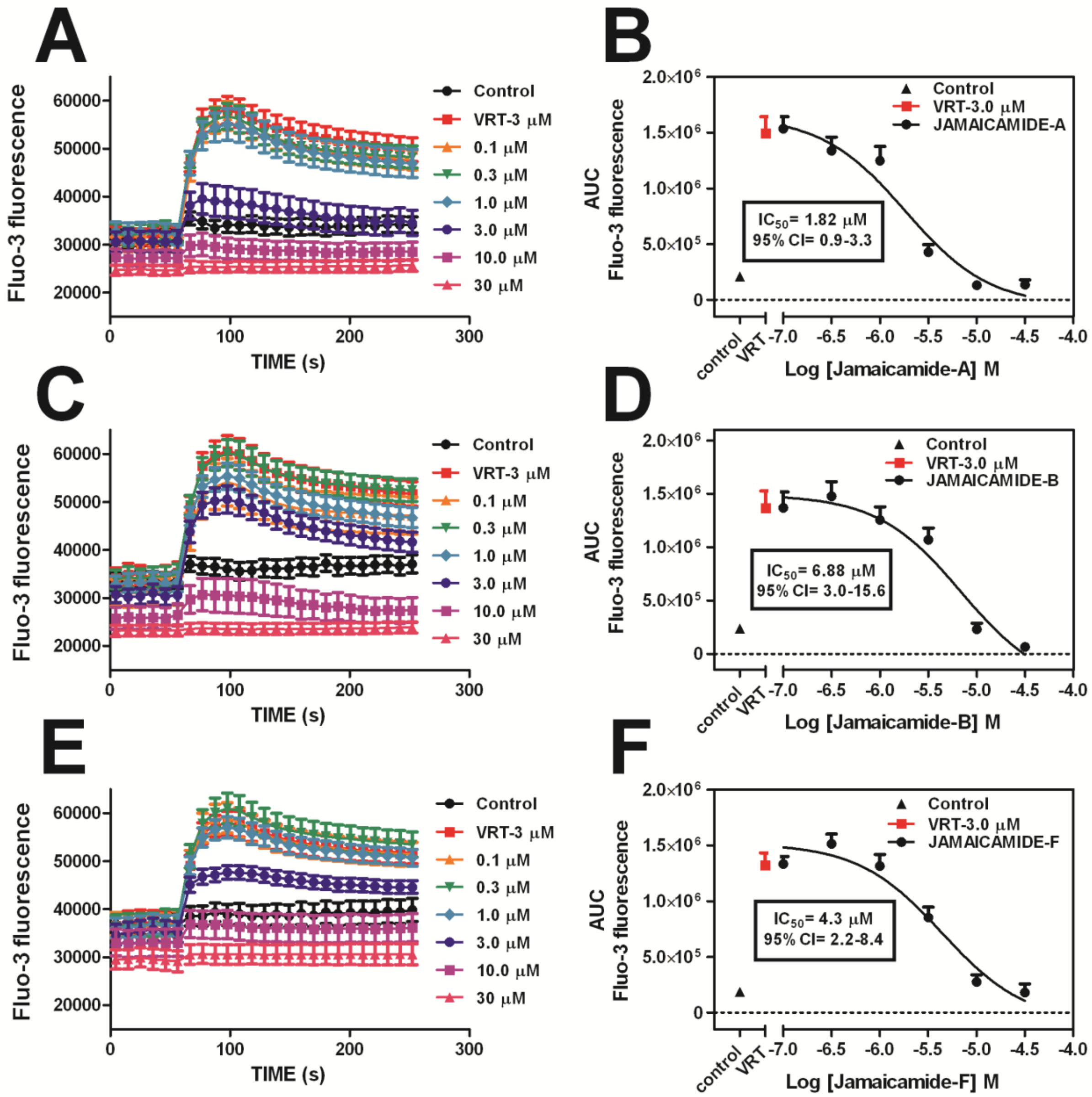

Panel A, C, E represents time response relationships for the decrease in veratridine-induced Ca^{2+} influx by jamaicamide A, B and F respectively. **Panel B, D, F** represents concentration-response relationships for the decrease of veratridine-induced Ca^{2+} influx by jamaicamide A, B and F (**2**, **3**, and **12**). This figure represents combined data from 5-6 experiments performed with 2-3 replicates each.

Figure AQ. Effect of the jamaicamides on the veratridine-induced Na^+ influx in murine neocortical neurons.

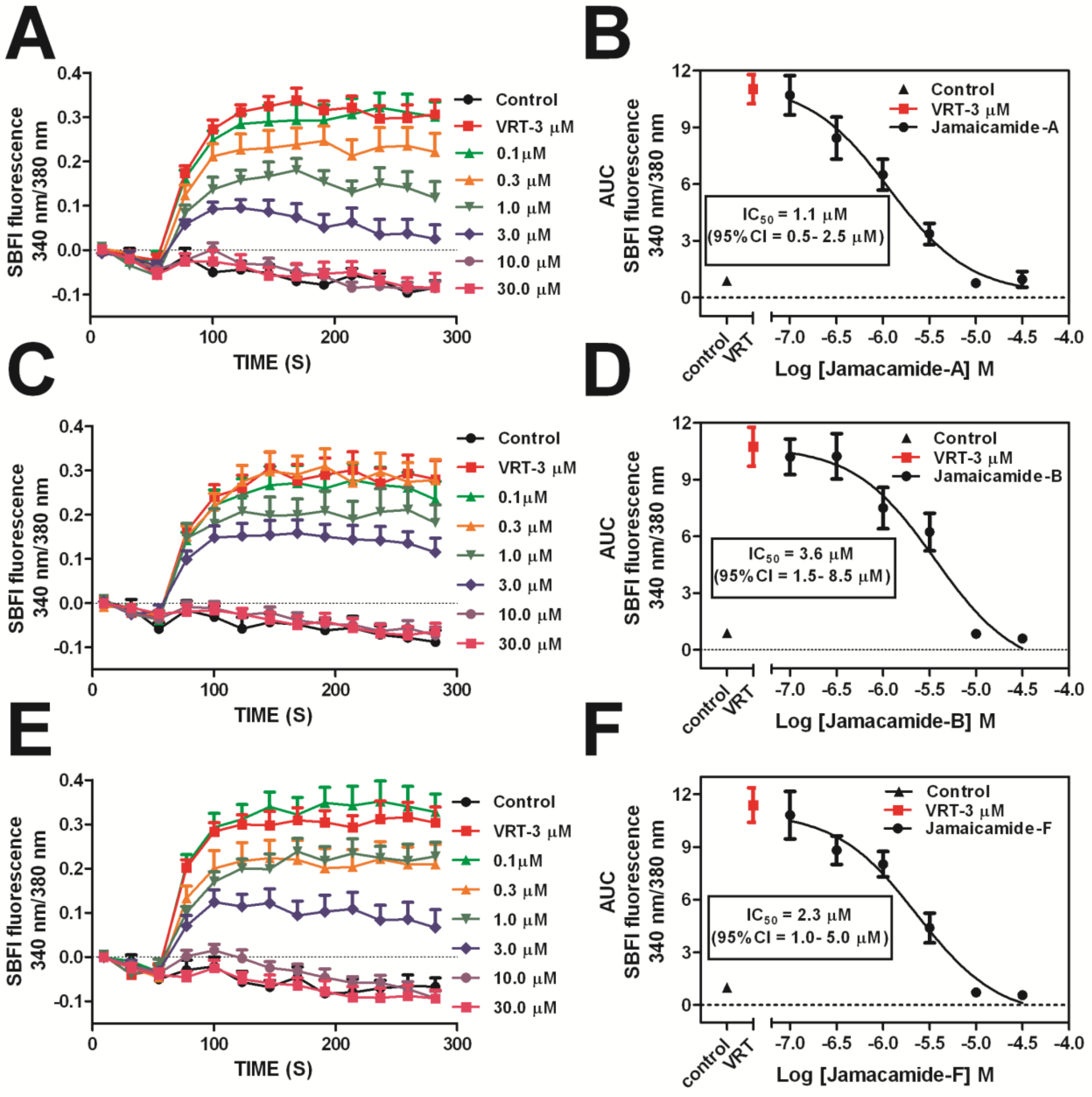

Panel A, C, E represents time response relationships for the reduction in the veratridine-induced Na^+ influx by jamaicamide A, B and F respectively. **Panel B, D, F** represents nonlinear regression analysis of the SBFI (340/380) responses to veratridine in the absence and presence of jamaicamide (A, B and F; **2**, **3**, and **12**). This figure represents combined data from 4 experiments performed with 3 replicates each.

Figure AR. ^{1}H-NMR (600 MHz, $CDCl_3$) Spectrum of Hectoramide (4).

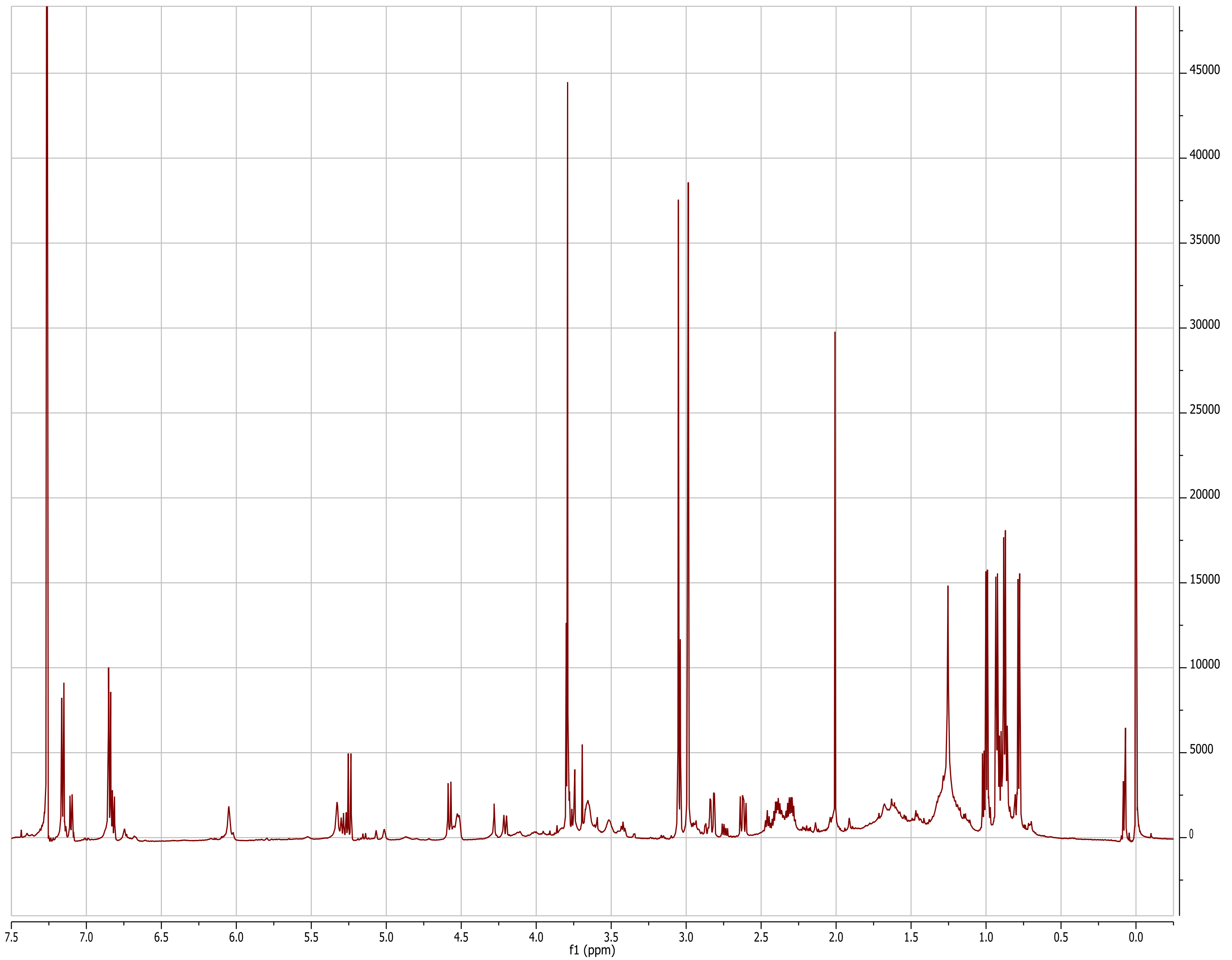

Figure AS. ^{13}C-NMR (125 MHz, $CDCl_3$) Spectrum of Hectoramide (4).

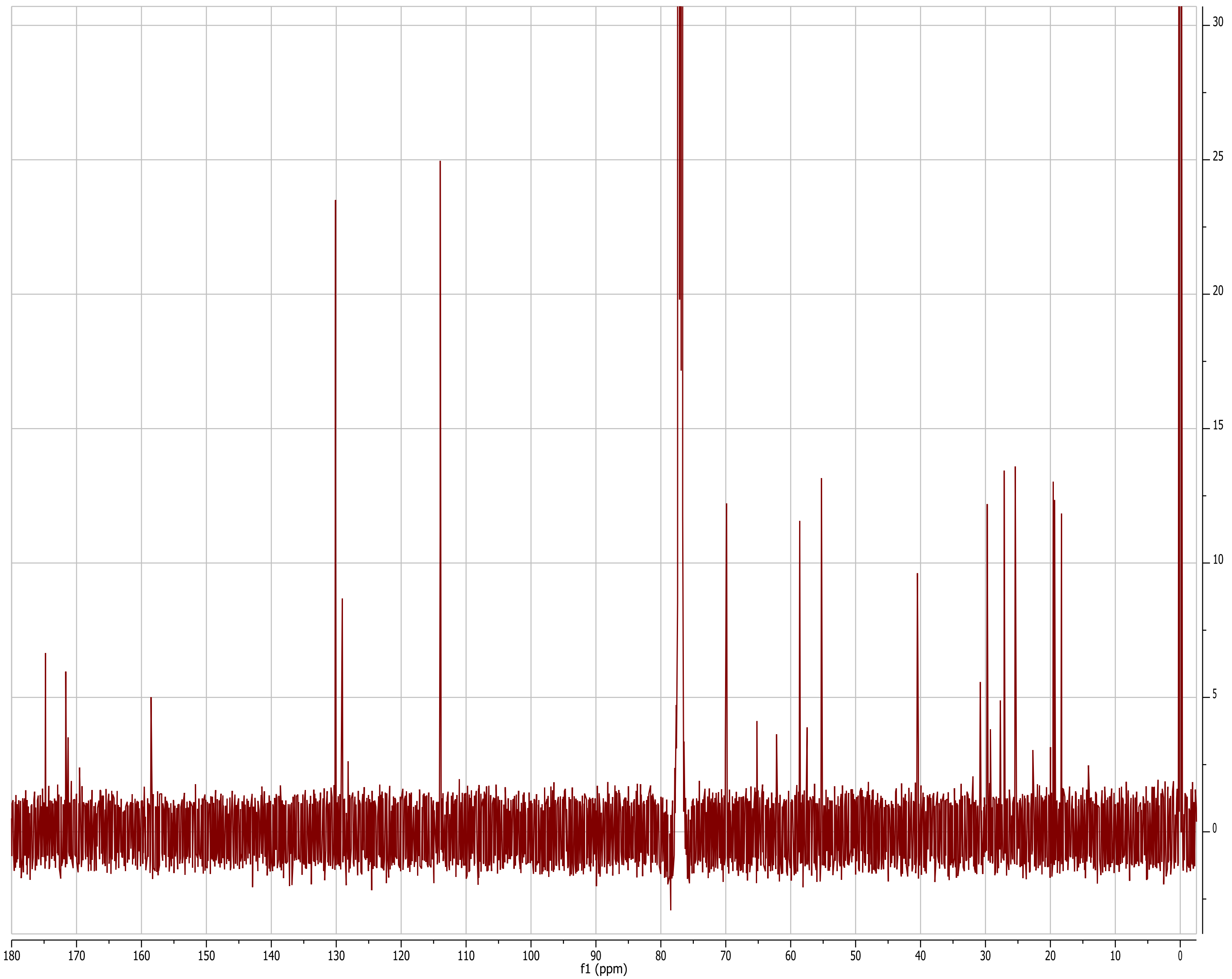

Figure AT. Predicted ^{13}C-NMR for Potential Structures of Hectoramide (4).

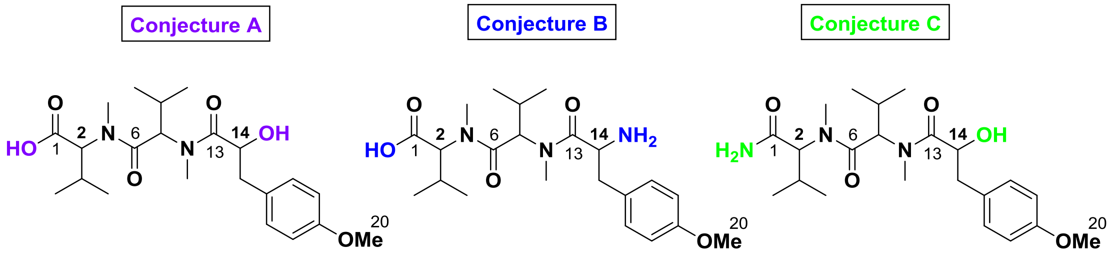

Postion, δ_C	Conjecture A ChemDraw δ_C Prediction, Difference	Conjecture B ChemDraw δ_C Prediction, Difference	Conjecture C ChemDraw δ_C Prediction, Difference
1, 171.3	175.7, -4.4	175.7, -4.4	176.7, -5.4
**2, 62.2**	**80.4, -18.2**	**80.4, -18.2**	**72.5, -10.3**
3, 25.4	27.7, -2.3	27.7, -2.3	26.7, -1.3
4, 18.2(8)	19.2, -0.9	19.2, -0.9	18.8, -0.5
5, 19.6	19.2, 0.4	19.2, 0.4	18.8, 0.8
6, 30.8	32.5, -1.7	32.5, -1.7	32.1, -1.3
7, 171.7	172.7, -1.0	172.7, -1.0	172.7, -1.0
8, 58.6	68.1, -9.5	67.8, -9.2	67.7, -9.1
9, 27.1	27.3, -0.2	27.3, -0.2	27.3, -0.2
10, 18.3(4)	18.8, -0.5	18.8, -0.5	18.8, -0.5
11, 19.4	18.8, 0.6	18.8, 0.6	18.8, 0.6
12, 29.7	32.4, -2.7	32.1, -2.4	32.4, -2.7
13, 174.8	168.9, 5.9	170.0, 4.8	168.9, 5.9
**14, 69.9**	**70.4, -0.5**	**51.2, 18.7**	**70.4, -0.5**
15, 40.5	41.5, -1.0	39, 1.5	41.5, -1.0
16, 129.1	128.3, 0.8	128.9, 0.2	128.3, 0.8
17, 130.1	129.8, 0.3	129.8, 0.3	129.8, 0.3
18, 114.0	114.2, -0.2	114.2, -0.2	114.2, -0.2
19, 158.5	157.8, 0.7	157.8, 0.7	157.8, 0.7
20, 55.3	55.8, -0.5	55.8, -0.5	55.8, -0.5

Figure AU. HSQC (600 MHz, $CDCl_3$) Spectrum of Hectoramide (4).

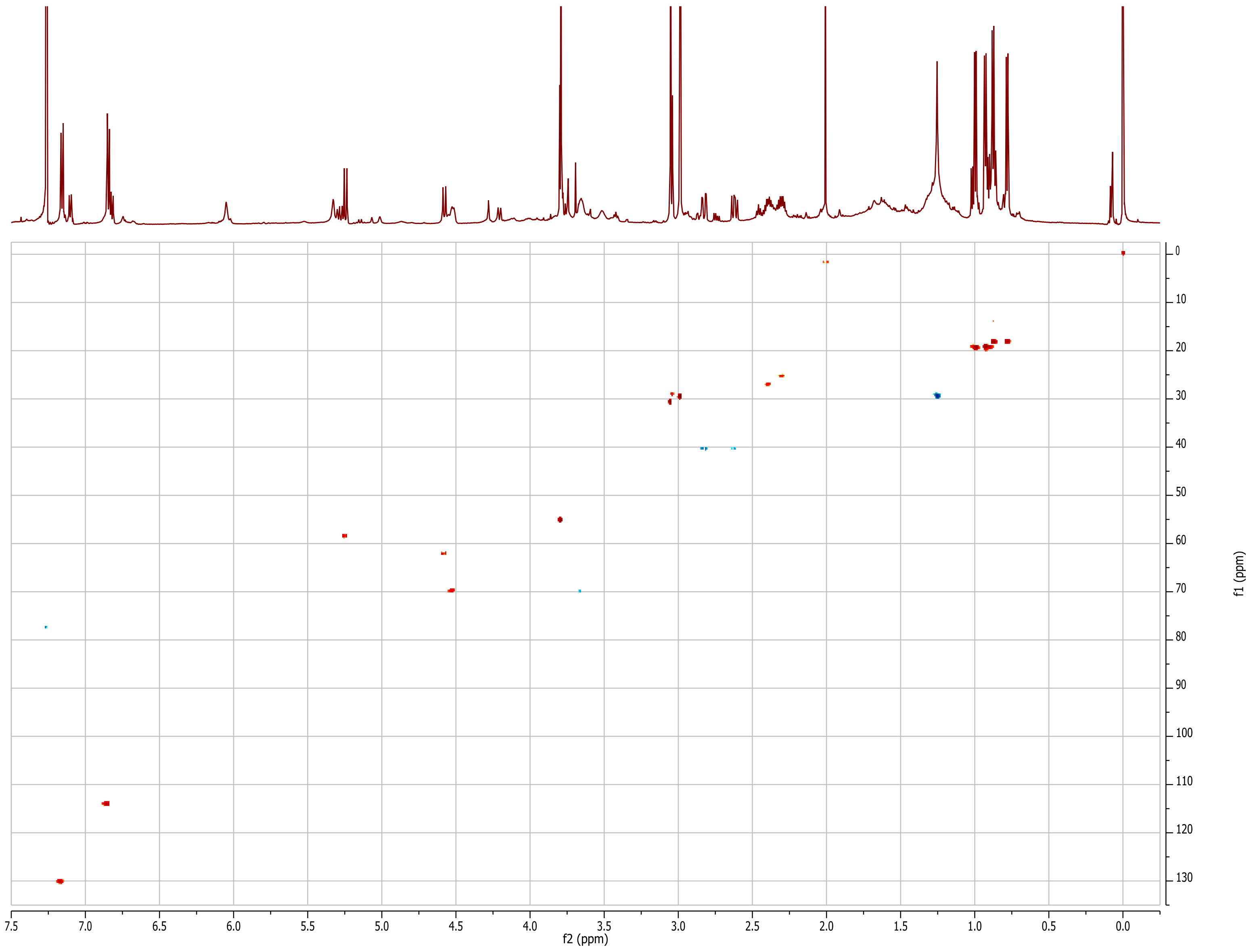

Figure AV. HMBC (600 MHz, $CDCl_3$) Spectrum of Hectoramide (4), Long Duration.

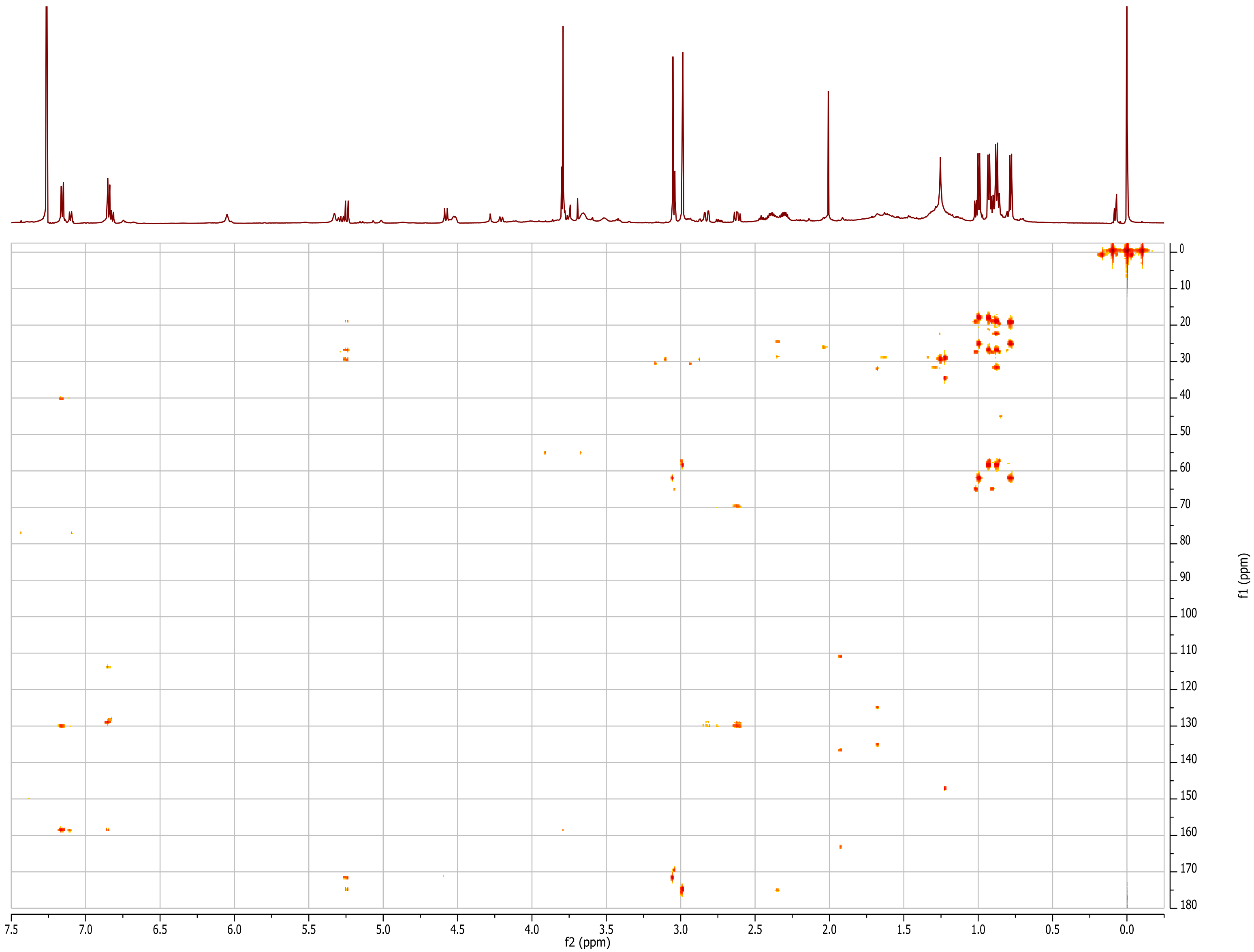

Figure AW. HMBC (600 MHz, $CDCl_3$) Spectrum of Hectoramide (4), Short Duration.

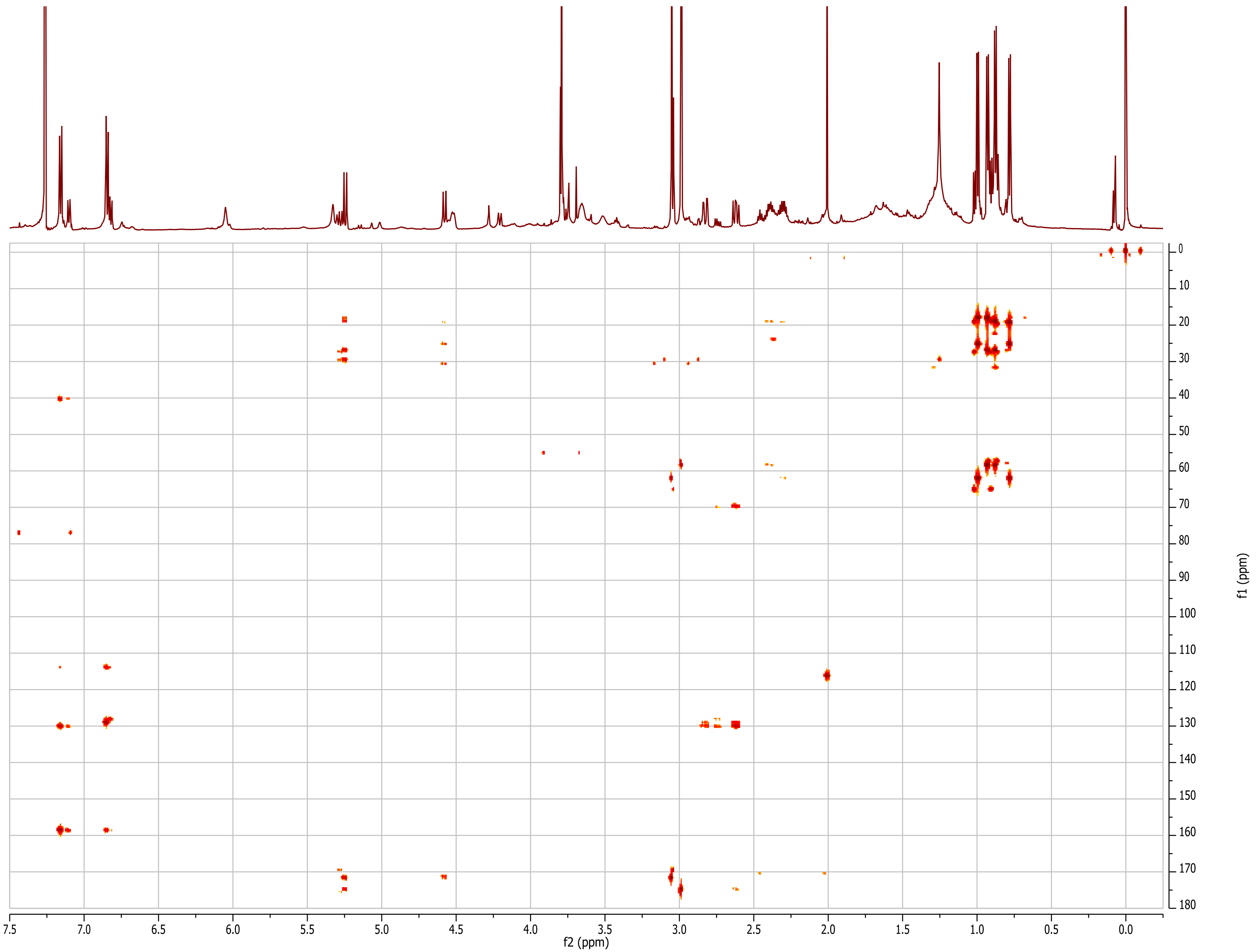

Figure AX. TOCSY (600 MHz, $CDCl_3$) Spectrum of Hectoramide (4).

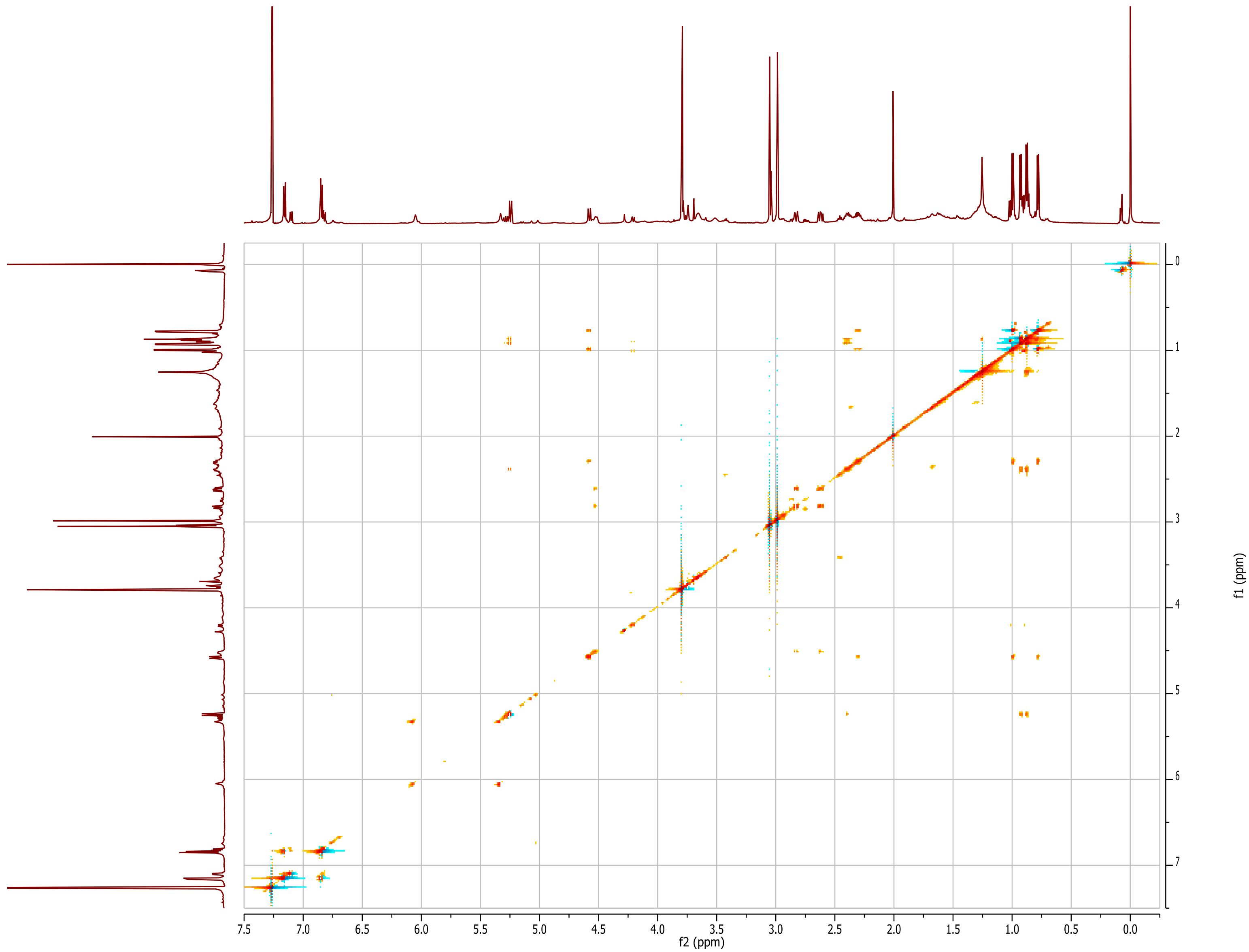

Figure AY. GC-MS Analysis of 2-S-Octonol Ester Standards.

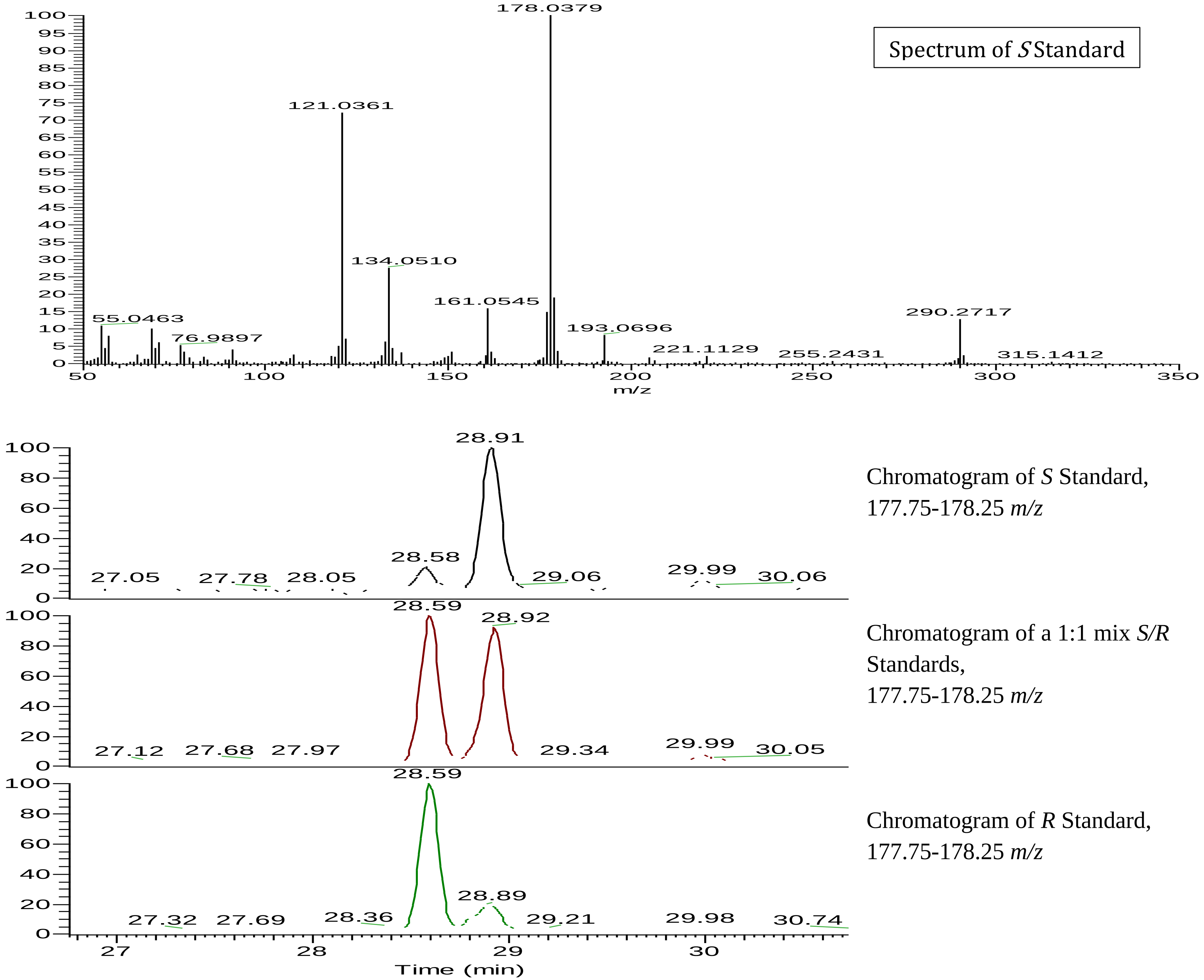

Chromatogram of *S* Standard, 177.75-178.25 *m/z*

Chromatogram of a 1:1 mix *S/R* Standards, 177.75-178.25 *m/z*

Chromatogram of *R* Standard, 177.75-178.25 *m/z*

Figure AZ. GC-MS Analysis of 2-S-Octonol Ester Derivatized Hydrolysate of Hectoramide (4).

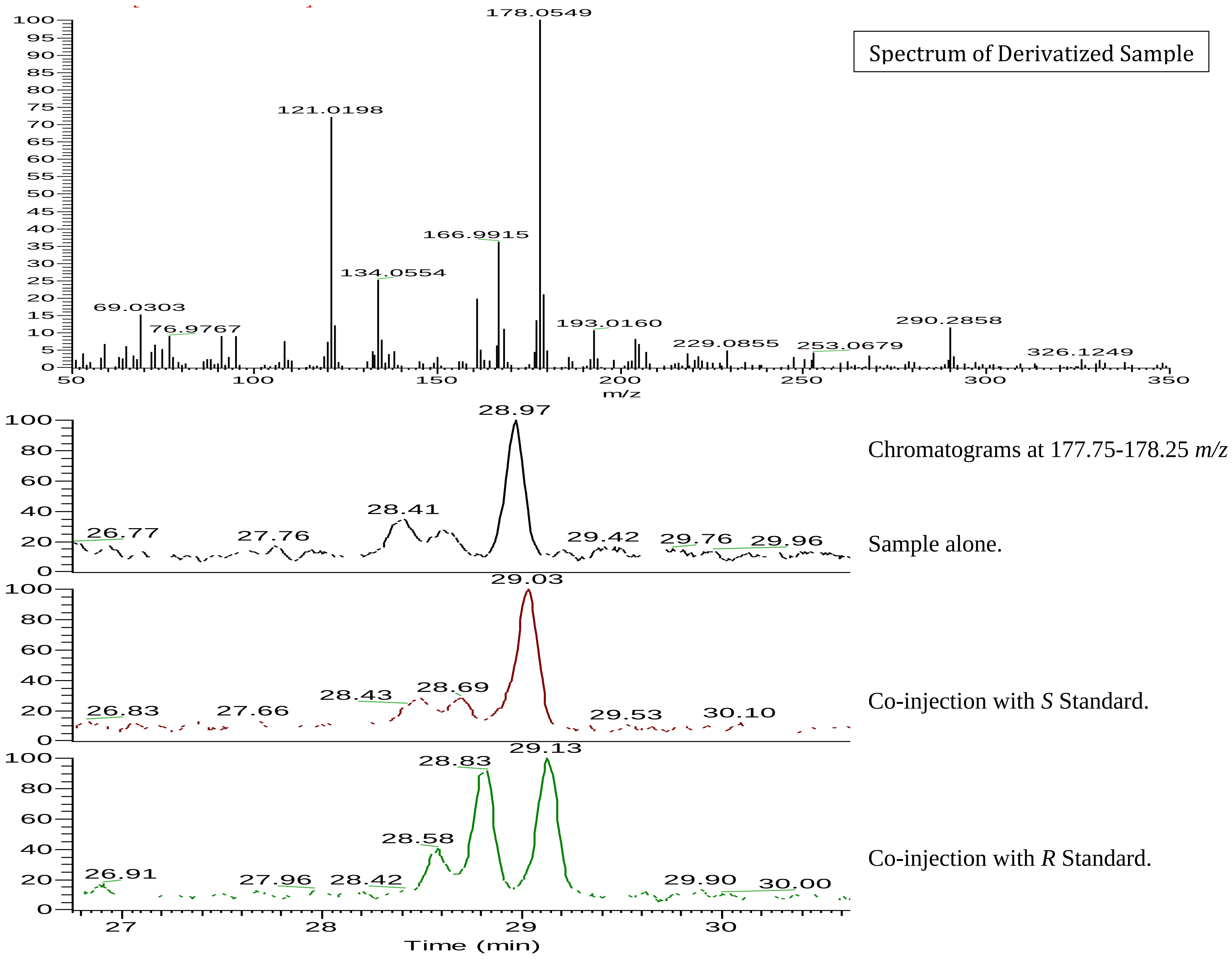

Figure AAA. Marfey's Analysis of D-FDAA Derivatized Hydrolysate of Hectoramide (4).

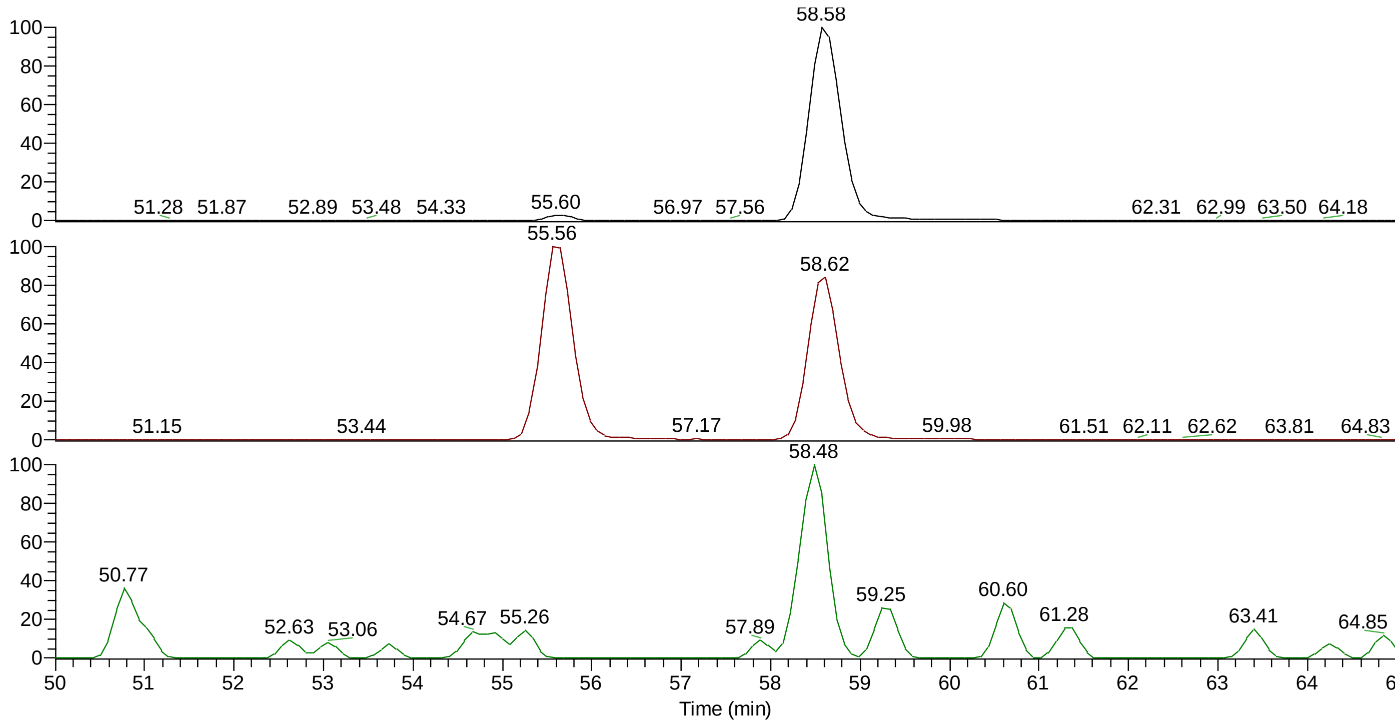

Chromatograms of 383.70-384.10 *m/z*

D-FDAA derivatized L-*N*-Me-Val standard.

D-FDAA derivatized D/L-*N*-Me-Val standard.

D-FDAA derivatized hydrolyaste.
